# About a Trillion Times More Acidic than Expected? On the Difference Between the Hammett *H*_0_ and the Unified pH Acidity of Sulfuric Acid

**DOI:** 10.1002/anie.202506429

**Published:** 2025-11-17

**Authors:** Valentin Radtke, Monika Bäuerle, Regina Stroh, Timo Kienzle, Daniel Himmel, Agnes Heering, Jaak Nerut, Enn Lust, Ivo Leito, Ingo Krossing

**Affiliations:** ^1^ Institut für Anorganische und Analytische Chemie and Freiburger Materialforschungszentrum (FMF) Universität Freiburg Albertstr. 21 79104 Freiburg Germany; ^2^ Institute of Chemistry University of Tartu Ravila 14a Tartu 50411 Estonia

**Keywords:** Computational chemistry, Hammett acidity function, Ionic liquid salt bridge, Sulfuric acid, Unified acidity

## Abstract

We report on the considerable difference between the distinct pH and Hammett's *H*_0_ acidity function in the entire H_2_O‐H_2_SO_4_ mixing range. The Hammett acidity for 100 wt% H_2_SO_4_ is accepted with *H*_0_ = −11.9. We measured the pH acidity using two different electrochemical cells, operating with at least one hydrogen electrode, giving for 100% sulfuric acid ${\mathrm{pH}}_{\mathrm{abs}}^{H_{2}O}$ as −24 ± 2. This value was confirmed by high‐level compound calculations using the DLPNO‐CCSD(T) level at the basis set limit and COSMO‐RS solvation. Thus, the difference between both scales amounts to ${\mathrm{pH}}_{\mathrm{abs}}^{H_{2}O}$ − *H*_0_ = −12 or 12 orders of magnitude. It results from the Gibbs transfer energies of the (protonated) Hammett‐indicator bases B (BH^+^) used to define and determine *H*_0_. High‐level computations of these Gibbs transfer energies confirm our experimental as well as our computational findings. A general method to convert *H*_0_ values of acids other than sulfuric acid into ${\mathrm{pH}}_{\mathrm{abs}}^{H_{2}O}$ values is given. Hence, this huge (pH − *H*_0_)‐difference clearly delineates that the often used but erroneous assumption of the *H*_0_ values constituting an extension of pH acidity to the realm of negative pH values is wrong. The distinctive protochemical potential curve progression is discussed as a function of sulfuric acid concentration and physical properties, together with the assignment of the potential‐determining species in the respective mixtures. Finally, superacidity is defined in a universal manner, i.e., applicable to all media, i.e., liquid, solid, or gaseous.

## Graphical abstract

**Take off the Glasses!** Indicator bases are tools for determining the widely used Hammett *H*_0_ acidity, which was used to look at pure sulfuric acid. Yet, this is like looking through sunglasses: despite their usefulness, they work as a filter. We take off the glasses and see unfiltered the protochemical pH acidity measured with the H_2_ electrode: the acidity of pure sulfuric acid is about 12 orders of magnitude higher than the indicator glasses show.

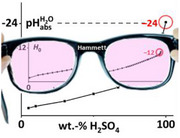

## Introduction

With an annual production of several hundred million tons worldwide, the “mother of all acids,” as sulfuric acid is sometimes called, represents one of the strongest commercially used Brønsted acids. It is not only extremely important from an economic point of view and as a starting material for many industrial and agricultural products, but its high acidity is also of academic relevance: R. J. Gillespie defined the acidity level of pure sulfuric acid as the threshold to Brønsted superacidity,^[^ ^1^ ^]^ a field of active research ever since.^[^ ^2^, ^3^, ^4^ ^]^ Therefore, it is most remarkable that the true acidity level, i.e., the protochemical potential of sulfuric acid, remains unknown so far. As a workaround, Gillespie used Hammett's acidity function *H*_0_ for his definition of superacidity. And although the *H*_0_ function is correlated with the proton's chemical potential, we show below that it is not the only major factor that determines its acidity level. Hence, what is the definite entry criterion for superacidity that can also be used in the manifold of other highly acidic diverse media like pure acids, acids in organic and inorganic solvents, and also the solid zeolites used industrially?

### Brønsted's Protochemical pH Acidity Versus Hammett's *H*_0_ Acidity Function

In his seminal work on acidity from 1923, Brønsted considered the chemical potential of the proton to be the only logically consistent measure of acidity, a notion that we adhere to.^[^ ^5^ ^]^ Sørensen had already anticipated that a few years earlier, when he defined the pH value as:^[^ ^6^ ^]^

(1) $$\mathrm{pH}\;=-\mathrm{lg}a\left(H^{+}\right)$$

The (dimensionless) relative activity *a* of any chemical entity is defined by its chemical potential *μ*.^[^ ^7^ ^]^ “Relative” means that the activity in Equation (1) is actually the ratio of the solvated H^+^ activity to the activity of H^+^ in the standard state in the respective medium. However, this implies that the pH value is valid only for one medium, i.e., Sørensen defined pH for aqueous solutions only. To remove the restriction to just one medium and to enable the acidities in all media to be compared, the medium‐specific standard state has to be eliminated. Therefore, the ideal proton gas at standard conditions^[^ ^8^ ^]^ was selected as solvent‐/medium‐free standard state. Its chemical potential was arbitrarily set to zero. From there, we defined the solvent‐independent unified pH_abs_ acidity scale^[^ ^9^, ^10^, ^11^, ^12^ ^]^ using the absolute activity based on the protochemical potential and in agreement with a new IUPAC technical paper:^[^ ^13^ ^]^

(2) $${\mathrm{pH}}_{\mathrm{abs}}=-\mathrm{lg}a_{\mathrm{abs}}\left(H^{+}\right)=-\frac{\mu_{\mathrm{abs}}\left(H^{+}\right)}{\mathrm{RT}\ln10}$$

Consequently, the pH acidity level of any medium is exclusively governed by the protochemical potential in this medium, i.e., the activity of its protons in comparison to gaseous protons. However, the determination of the protochemical potential in non‐aqueous media relative to the gaseous proton (or to the aqueous proton as well) is anything but easy, especially when conditions are as harsh as in sulfuric acid. Therefore, as workaround, Hammett and Deyrup defined and determined the *H*_0_ acidity as “*acidity in terms of a basic indicator*” of concentrated and pure (super)acids, inter alia sulfuric acid:^[^ ^14^ ^]^

(3) $$H_{0}=-\mathrm{lg}\left(a\left(H^{+}\right)\frac{f_{B}}{f_{BH^{+}}}\right)$$

However, this contrasts with the pH acidity, because the *H*_0_ acidity function depends not exclusively on the protochemical potential but also on the activity coefficients *f*_B_ and *f*_BH_^+^ of an indicator base B and its protonated form BH^+^. Although pH and *H*_0_ acidity are two different scales, we compare the values with each other. The reasons for this are that the *H*_0_ scale is the most widely used for sulfuric acid and other superacids, and that the two are sometimes used interchangeably, but clearly, the *H*_0_ scale is not a continuation of the pH scale from the aqueous range into the highly acidic range, as is often assumed or propagated in articles and in textbooks.^[^ ^15^, ^16^ ^]^ And even if someone clearly differentiates between the two scales, it can easily happen, in the absence of data on protochemical potential, that this person is of the opinion that “the difference should not be too big.” Interested in how large this difference might be (and what the boundary to superacidity is in terms of the protochemical potential), in 2010 we realized for the first time through computations that the difference could be huge and be larger than 10 orders of magnitude,^[^ ^10^, ^11^ ^]^ which prompted us to investigate this in more detail.

Hammett himself did **not** hypothesize that the ratio *f*_B_/*f*_BH_^+^ is unity (or approximately unity) at high acidity levels, but that “*in a given solution is the same for all bases*” (which they used).^[^ ^14^ ^]^ Indeed, for **dilute** aqueous acid solutions, *f*_B_/*f*_BH_^+^ is in good approximation unity, and *H*_0_ is therefore equal to the pH value. However, in more concentrated acid solutions, this ratio deviates more and more from unity, and at high acidity levels, *H*_0_ and pH values are increasingly separated. In the limiting case of pure and strong acids, this separation can be enormous, as we show with this work.

To quantify the extent of this separation for sulfuric acid as a model acid and to assign the true protochemical potential level in pure sulfuric acid as well as its mixtures with water, we developed the methods described hereafter. Since this includes rather challenging experimental and theoretical work with the highly protonating, dehydrating, and oxidizing pure sulfuric acid, we split the investigation into distinct Results and Discussion sections.

## Results

Prior to describing the methods and their results, we need to clarify the formal notations used in this work. Equations (1) and (3) above are given in the usual notation, i.e., as in the original literature or taken from textbooks. However, it is necessary to include additional notation in order to assign distinct states and quantities. The index at the bottom right indicates which reference state is used for the quantity, e.g., ${\mathrm{pH}}_{H_{2}O}$ values refer to the (hypothetical) state of the protons in water at a standard concentration of 1 mol L^−1^ and that behave like the infinitely dilute solution with no interactions between the protons, as defined by IUPAC.^[^ ^7^ ^]^ The particle involved and the medium in which it is present are placed in parentheses. For example, since Sørensen had defined the pH value for aqueous solutions, Equation (1) is now written as ${\mathrm{pH}}_{H_{2}O}$ = −lg *a* 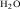
(H^+^, H_2_O). This can be generalized for all solvents or their mixtures, denoted as S. Hence, Equation (1) transforms to pH_S_ = −lg *a*_S_(H^+^, S), which results in pH_S_ values incomparable between different solvents S due to the need for S‐specific reference states. By contrast, the subscript “abs” refers to the state of a particle in the gas phase at 298.15 K and 10^5^ Pa. The resulting pH_abs_, as in Equation (2), is independent of the medium, and all values within this scale are comparable.^[^ ^10^, ^11^, ^12^, ^17^, ^18^ ^]^

Further, all values, including the activity coefficient *f*, are given in the molar scale. According to Popovych and Bates,^[^ ^19^ ^]^ it is termed salt effect _s_ *f* if given with the same reference state as the activity, e.g., *a* 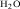 = _s_ *f* · *c* 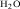. It is termed medium effect _m_ *f* if the activity of a chemical entity in non‐aqueous S refers to the aqueous reference state, i.e., *a* 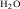 = _m_ *f* · *c*_S_ or *a* 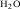 = _m_ *f*° · *a*_S_, respectively. The standard medium effect _m_ *f*° = _m_ *f*/_s_ *f* describes the standard Gibbs transfer energy of a particle i from water into S:^[^ ^20^ ^]^

(4) $$\Delta_{\mathrm{tr}}G^{\circ }\left(i,\;H_{2}O\to S\right)=\mathrm{RT}\ln_{m}f^{\circ }\left(i,\;H_{2}O\to S\right)$$

However, the above medium effect must **not** to be confused with the "*medium effect*” used by Hammett and Deyrup to account for a possible shift in spectral absorption by the indicator associated with changes in the test solution and that are **not** related to its acidity. Since Hammett and Deyrup referred all activities to the infinitely diluted aqueous solution, the complete and exact Equation (3) reads as follows:

(3b)

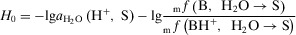




If a superscript is indicated at the top right, this means that the zero point (**not** the reference state) of this scale is used; the reference state still appears at the bottom right. In the solvent‐independent unified ${\mathrm{pH}}_{\mathrm{abs}}^{H_{2}O}$ scale^[^ ^13^ ^]^ ($E_{\mathrm{abs}}^{H_{2}O}$ scale) this implies that the reference state is the proton (electron) in the gas phase at 298.15 K and 10^5^ Pa, but that this state is **not** the zero point,^[^ ^21^, ^22^ ^]^ but rather that of the ${\mathrm{pH}}_{H_{2}O}$ ($E_{H_{2}O}$) scale. This was done to facilitate comparison of acidity levels of as many as possible entries in relation to the prototypical solvent for acid–base and redox reactions: water. Hence, the ${\mathrm{pH}}_{\mathrm{abs}}^{H_{2}O}$ scale can be defined by one of the several possible and equivalent descriptions given as Equation (5a–f):

(5a) $${\mathrm{pH}}_{\mathrm{abs}}^{H_{2}O}=pH_{\mathrm{abs}}+\frac{\Delta_{\mathrm{solv}}G^{\circ }\left(H^{+},\;H_{2}O\right)}{RT\ln10}$$

(5b) $$=pH_{S}-\frac{\Delta_{\mathrm{tr}}G^{\circ }\left(H^{+},\;H_{2}O\to S\right)}{RT\ln10}$$

(5c)

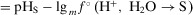

(5d) $$=-\mathrm{lg}a_{H_{2}O}\left(H^{+},\;S\right)$$

(5e) $$=-\frac{E_{\mathrm{abs}}^{H_{2}O}\left(H^{+}/H_{2},\;S\right)}{\left(\frac{RT\ln10}{F}\right)}$$

(5f)

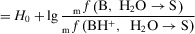




An absence of indexing merely implies that the reference point is not relevant to the discussion, e.g., bare pH indicates acidity exclusively in terms of the protochemical potential.

At constant pressure and temperature, the Gibbs energy of solvation of a particle i in the solvent S, Δ_solv_ *G*(i, S), corresponds to its chemical potential, i.e., *μ*_abs_(H^+^, S) = Δ_solv_ *G*(H^+^, S). We use Δ_solv_ *G*°(H^+^, H_2_O) = −1104.5 kJ mol^−1^ as the anchor magnitude to tie the scale to the proton gas standard state.^[^ ^23^ ^]^ The difference of the Gibbs solvation energies then results in the Gibbs transfer energy, i.e.,

$$\Delta_{\mathrm{solv}}G\left(H^{+},S\right)-\Delta_{\mathrm{solv}}G\left(H^{+},H_{2}O\right)=\Delta_{\mathrm{tr}}G\left(H^{+},H_{2}O\to S\right)$$

In the following, we denote sulfuric acid and its mixtures with water simply as S. Hence, each mixture, or also pure sulfuric acid is independent S_i_ or S_j_, with each i, j representing a mixing ratio between 1 and 100 wt% H_2_SO_4_.

### Overview to the Methods Used

Pure sulfuric acid is very challenging to work with. Therefore, we developed and evaluated four independent approaches to determine the protochemical potential in pure sulfuric acid—three of them can also be used for aqueous sulfuric acid—together with an assessment of their reliability (Methods section). Two are strictly experimental, the electrochemical SSE and ILSB methods; the QMB method is semi‐empirical and uses rigorously determined experimental $pK_{a,H_{2}O}$ values (converted into $pK_{a,H_{2}{\mathrm{SO}}_{4}}$ values by Equation (M3) given in the Method Section) of protonated reference bases B, in our case those that were used by Hammett and others, while the QME method is purely computational. Both QMB and QME employ highly accurate quantum chemical computations using double‐hybrid DFT structures of the investigated particles and the single‐point coupled cluster calculations DLPNO‐CCSD(T)^[^ ^24^, ^25^ ^]^ at the complete basis set limit (CBS) approximate the “gold standard” in the gas phase. Solvation Gibbs energies were computed with the empirically adjusted quantum‐chemical method COSMO‐RS, described as being “best‐in‐class.”^[^ ^26^, ^27^, ^28^ ^]^ The QMB and QME methods are distinguished by the chemical entities that were computed and used in the thermodynamic cycles.

### The QMB‐Method to Assess the Chemical Potential of the Proton in Sulfuric Acid

**Figure 1:**
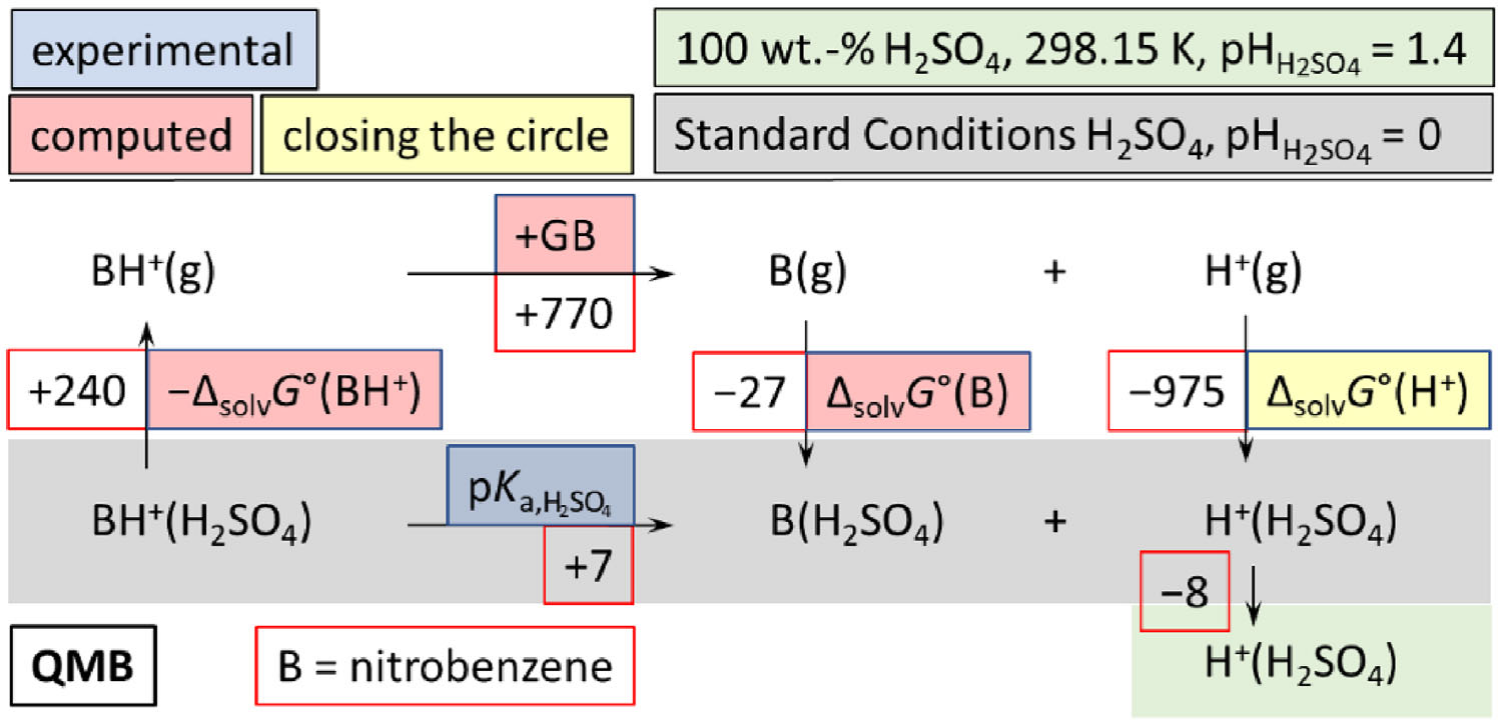
BFHC used to assess the Gibbs energy of solvation of the proton in 100 wt% sulfuric acid with the QMB method. The exemplary worked example (protonated) nitrobenzene is included. The values are given in kJ mol^−1^. From the autoprotolysis constant of sulfuric $pK_{\mathrm{ap},H_{2}{\mathrm{SO}}_{4}}$ = 3.10 and its self‐dehydration constant $pK_{\mathrm{ip},H_{2}{\mathrm{SO}}_{4}}$ = 3.85 the ${\mathrm{pH}}_{H_{2}{\mathrm{SO}}_{4}}$(H_2_SO_4_) = 1.4 is obtained (see below).

The *quantum chemically computed energetics of reference bases* (QMB) calculates the chemical potential of the proton in pure H_2_SO_4_ via the computed Gibbs solvation energies of the indicator bases B (pragmatically those used for the *H*_0_ determination) and their protonated forms BH^+^ via the Born–Fajans–Haber Cycle (BFHC) in Figure 1. The experimental acidity constants of the protonated bases referring to the sulfuric acid reference state $pK_{a,H_{2}{\mathrm{SO}}_{4}}$ were obtained according to Equation (M3) in the Method section. This implies that—as for both experimental methods—it is irrelevant to have any information on the distinct nature of the solvation of the proton in the medium, e.g., how the entities from pure sulfuric acid coordinate to (H^+^, H_2_SO_4_).

All data to close the BFHC in Figure 1 for eight typically employed reference bases B are collected in Table 1. The QMB method yields eight individual ${\mathrm{pH}}_{\mathrm{abs}}^{H_{2}O}$ values for pure sulfuric acid that range from −21.0 to −25.7 and yield an average ${\mathrm{pH}}_{\mathrm{abs}}^{H_{2}O}$ value of −22.1. This agrees within the error bars to our initial value computed in 2010 with the rCCC model for pure H_2_SO_4_ (${\mathrm{pH}}_{\mathrm{abs}}^{H_{2}O}$ = −22.8)—the starting point for this work.^[^ ^10^, ^11^ ^]^ For comparison and to demonstrate that this method is less influenced by explicit solvation, the ${\mathrm{pH}}_{\mathrm{abs}}^{H_{2}O}$ values calculated with Gibbs solvation energies from the simpler solvation model CPCM^[^ ^29^, ^30^, ^31^ ^]^ obtained at the DLPNO‐CCSD(T)/CBS level of theory are given in parentheses in Table 1; they range from −22.0 to −24.9 and yield on average a ${\mathrm{pH}}_{\mathrm{abs}}^{H_{2}O}$ value of −22.5. Hence, the QMB method, in principle designed to extract the chemical potential of the proton in sulfuric acid from the published *H*_0_ value of −11.9, suggests that the contribution of the Hammett bases influencing the measured *H*_0_ acidity function may be very large. Independently, the computed values can be used to assess the experimental ${\mathrm{pH}}_{\mathrm{abs}}^{H_{2}O}$‐ or *H*_0_‐values (see below “Quantification of the Differences between ${\mathrm{pH}}_{\mathrm{abs}}^{H_{2}O}$ and *H*_0_ in Pure H_2_SO_4_”).

**Table 1:** Thermodynamically consistent $pK_{a,H_{2}{\mathrm{SO}}_{4}}$‐values of the Hammett indicator bases in the sulfuric acid reference state, $pK_{a,H_{2}{\mathrm{SO}}_{4}}$(BH^+^, H_2_SO_4_), (Equation M3), along with all the calculated energies required to complete the thermodynamic cycle, such as the solvation energy of species BH^+^, Δ_solv_ *G*°(BH^+^), the solvation energy of B, Δ_solv_ *G*°(B), the gas phase basicity, GB (Δ_g_ *G*°), at the DLPNO‐CCSD(T)/CBS level of theory, and the resulting proton solvation Gibbs energy Δ_solv_ *G*°(H^+^, H_2_SO_4_) (at ${\mathrm{pH}}_{H_{2}{\mathrm{SO}}_{4}}$ = 0) in kJ mol^−1^, QMB‐BFHC (Figure 1), and the resulting ${\mathrm{pH}}_{\mathrm{abs}}^{H_{2}O}$ values in 100 wt% H_2_SO_4_ (at ${\mathrm{pH}}_{H_{2}{\mathrm{SO}}_{4}}$ = 1.4^[^ ^32^ ^]^) (Equations 2 and 5) with its average. The solvation energies were calculated in pure sulfuric acid as modeled with COSMO‐RS^[^ ^26^, ^27^, ^28^ ^]^ and as a cross‐check also with the CPCM model in parenthesis.^[^ ^29^, ^30^, ^31^ ^]^

Base B	$pK_{a,H_{2}O}$ ^[^ ^33^ ^]^ (BH^+^, H_2_SO_4_)	$pK_{a,H_{2}{\mathrm{SO}}_{4}}$ (BH^+^, H_2_SO_4_)	GB (kJ mol^−1^)	Δ_solv_ *G°*(BH^+^) (kJ mol^−1^)	Δ_solv_ *G°*(B) (kJ mol^−1^)	Δ_solv_ *G*° (H^+^, H_2_SO_4_) (kJ mol^−1^)^a^ ^)^	${\mathrm{pH}}_{\mathrm{abs}}^{H_{2}O}$ ^b)^
2,4,6‐trinitroaniline	−10.10	3.23	742.4	−287.1	−61.5	−949.6	−25.7
				(−269.5)	(−39.1)	(−954.4)	(−24.9)
p‐nitrotoluene	−11.35	1.98	785.2	−232.9	−31.2	−975.6	−21.2
				(−214.4)	(−17.5)	(−970.8)	(−22.0)
m‐nitrotoluene	−11.99	1.34	778.5	−234.5	−28.7	−976.7	−21.0
				(−216.3)	(−16.9)	(−970.2)	(−22.1)
nitrobenzene	−12.14	1.19	769.6	−239.7	−27.1	−975.4	−21.2
				(−224.8)	(−16.4)	(−971.2)	(−22.0)
p‐nitrofluorobenzene	−12.44	0.89	763.3	−241.3	−25.6	−974.0	−21.5
				(−229.9)	(−17.4)	(−970.7)	(−22.0)
p‐nitrochlorobenzene	−12.70	0.63	764.0	−239.5	−26.9	−972.9	−21.6
				(−226.4)	(−15.9)	(−970.9)	(−22.0)
m‐nitrochlorobenzene	−13.20	0.13	753.7	−244.5	−24.4	−973.1	−21.6
				(−230.5)	(−15.2)	(−968.3)	(−22.5)
2,4‐dinitrotoluene	−13.74	−0.41	745.3	−260.1	−44.4	−963.4	−23.3
				(−250.0)	(−29.2)	(−968.4)	(−22.4)
**Average**							−22.1 (−22.5)

^a)^ 8 kJ mol^−1^ (1.4) have to be added to get the state of pure sulfuric acid ($pH_{H_{2}SO_{4}}$ = 1.4).

^b)^ 1.4 (8 kJ mol^−1^) have to be subtracted to get standard conditions of an ideal solution of 1 mol L^−1^ ($pH_{H_{2}SO_{4}}$ = 0).

### SSE‐ and ILSB‐Methods to Measure the Chemical Potential of the Proton in Sulfuric Acid

#### Experimental Measurement Setup

**Figure 2:**
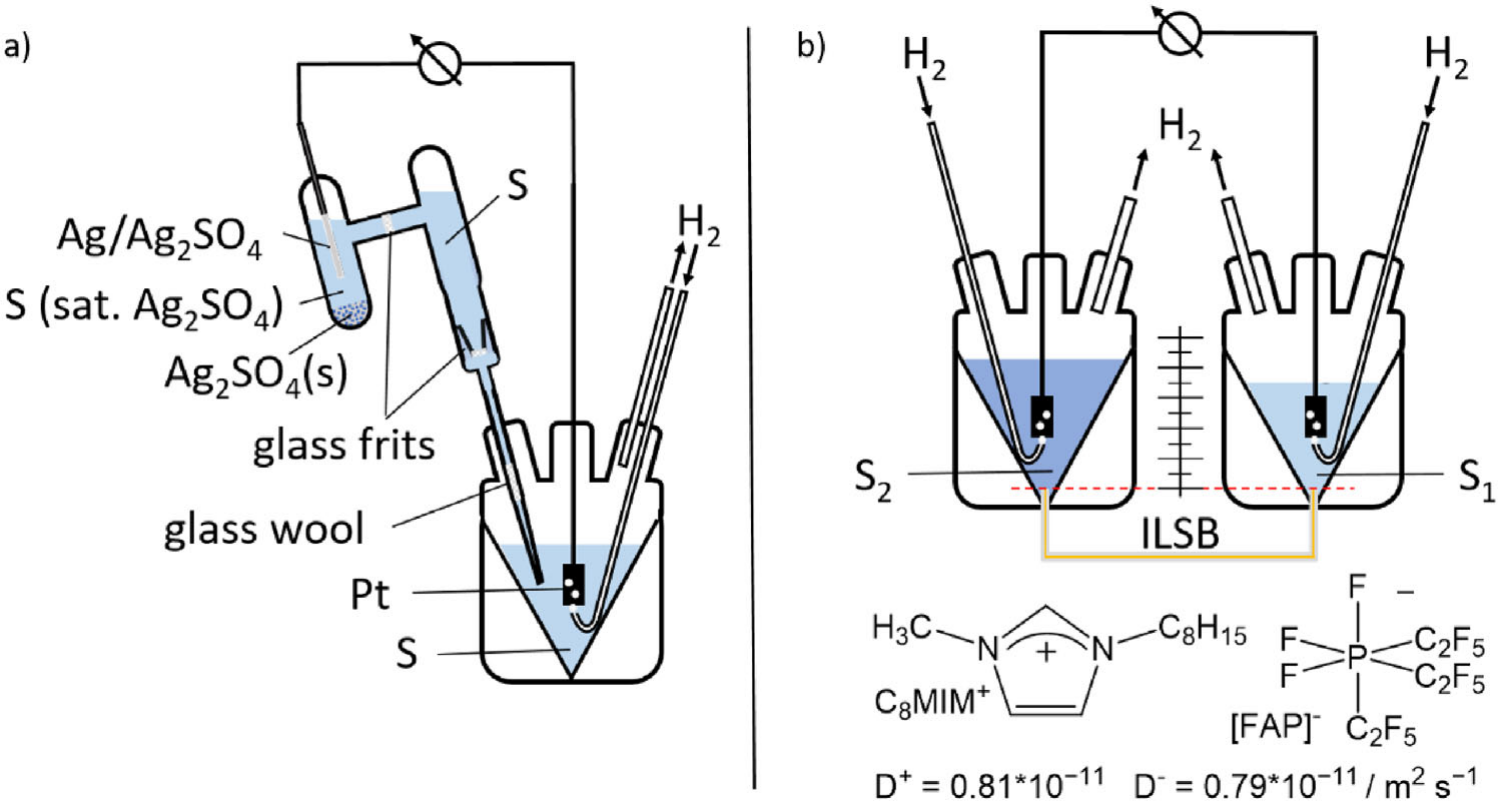
Measurement setups for the SSE and ILSB methods. a) The SSE uses a saturated silver/SSE as the reference electrode for a hydrogen electrode and operates with cell I. b) The ILSB‐method measures with cell II the electric potential difference *E*_II_ of two hydrogen electrodes in different H_2_O‐H_2_SO_4_ mixtures S_1_ and S_2_, connected by a SB with a pure and “ideal” IL. The structure of this “ideal” C_8_MIM[FAP] IL, compatible even with 100 wt% H_2_SO_4_, and the diffusion constants *D* ^+/−^ of the constituent ions as measured by pulsed field gradient spin echo NMR‐spectroscopy are included as inset.

The principles of both electrochemical methods are shown in Figure 2. The SSE setup (Figure 2a) contains a saturated silver/silver sulfate electrode^[^ ^34^ ^]^ (SSE) as the reference electrode for a hydrogen electrode and operates with cell I:

(**cell** **I**) $$\mathrm{Ag}|{\mathrm{Ag}}_{2}{\mathrm{SO}}_{4}|{\mathrm{Ag}}_{2}{\mathrm{SO}}_{4}\left(\mathrm{sat}.,\;S\right)\;\vdots \;S\;\vdots \;S\left|H_{2}\right|\mathrm{Pt}.$$

Typically, the potential of the SSE depends on the activity of the SO_4_^2−^ ion but is independent of the activity of the proton. Thus, with its help the change of the protochemical potential can be sensed as a function of the H_2_SO_4_ content in S.

For the ILSB‐method,^[^ ^18^, ^35^ ^]^ the measured quantity is the electric potential difference *E*_II_ of two electrochemical half‐cells, operated with hydrogen electrodes, and connected by a salt‐bridge (SB) with a pure and “ideal” ionic liquid (IL).

(**cell** **II**) $$\mathrm{Pt}\left|H_{2}\right|H_{2}SO_{4}\left(S_{j}\right)\underset{Ej_{2}}{|}C_{8}\mathrm{MIm}\left[\mathrm{FAP}\right]\underset{Ej1}{|}H_{2}SO_{4}\left(S_{i}\right)\left|H_{2}\right|\mathrm{Pt}\;$$

The “ideal” IL used in this work (Figure 2b) was experimentally shown to be compatible with all acidity and reducity levels up to the stage of 100 wt% sulfuric acid; it is C_8_MIm[FAP]—1‐methyl‐3‐octyl‐imidazolium tris(perfluoroethyl)trifluorophosphate with a ratio of its ion diffusion constants *D* ^+^/*D^−^* of 50.9/49.1% in the neat IL. Hence, the magnitude of the difference of the diffusion potentials at the junction *E*_j1_ and *E*_j2_ is minimized, supported by a series of further experiments described in Section S1.3.^[^ ^18^, ^21^, ^35^, ^36^ ^]^ Since cell II consists of two hydrogen electrodes in different H_2_O‐H_2_SO_4_ mixtures, the difference of two protochemical potentials is measured. This is as close as we can approach Brønsted's ideal of the degree of acidity.

Care was taken in both setups that the concentration of the sulfuric acid was correct. This was done by including a conductivity measurement cell into the compartments and calibrating the H_2_SO_4_‐concentration on its known conductivities.^[^ ^37^ ^]^ To obtain 100 wt% H_2_SO_4_ we added fuming sulfuric acid (a mixture of H_2_SO_4_ with SO_3_) dropwise to 98 wt% H_2_SO_4_ and waited for the system to respond until the minimum conductivity of 10.4 mS·cm^−1^ was reached, since the conductivity of aqueous and fuming sulfuric acid is higher than that of pure sulfuric acid. With this procedure, any water present within the cell was scavenged, and we were sure to investigate the correct concentration. For more details on reliability and uncertainty evaluation, we refer to the Methods section and the Supporting Information.

#### Chemical Potentials of the Proton in H_2_O‐H_2_SO_4_ Mixtures

**Figure 3:**
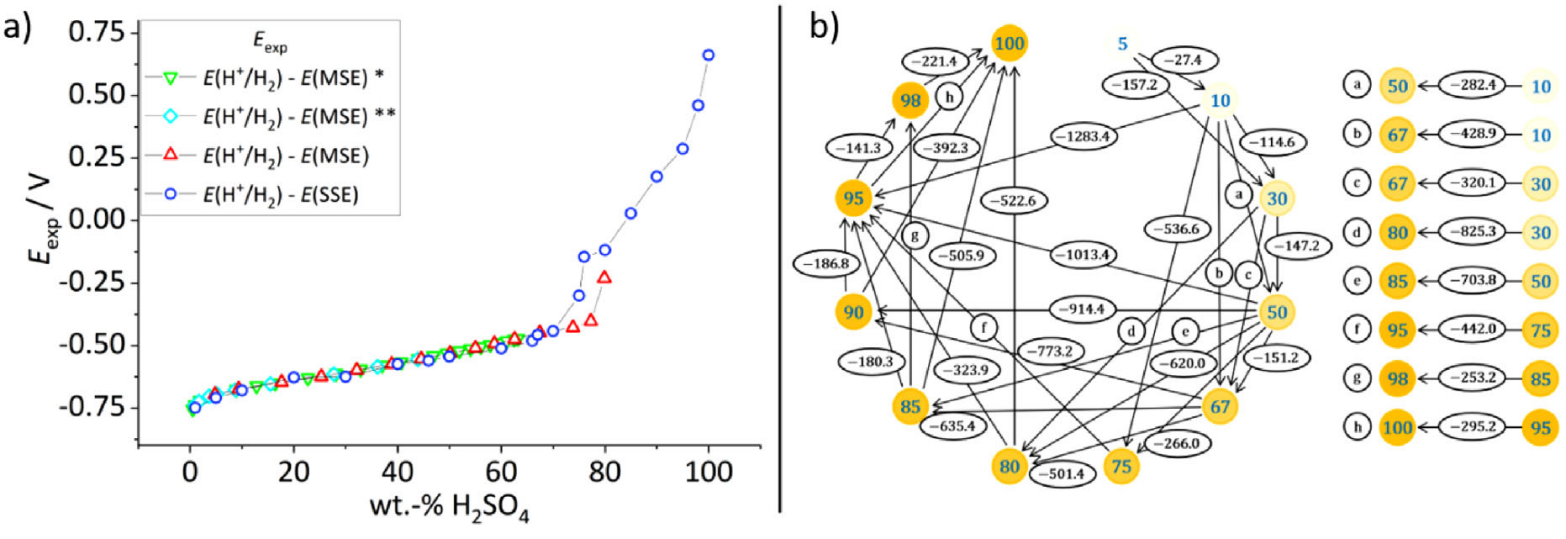
Results of the experimental SSE a) and ILSB methods b) to determine the ${\mathrm{pH}}_{\mathrm{abs}}^{H_{2}O}$ values in sulfuric acid‐water mixtures. a) Measured potential values *E*_I_ of cell I with the saturated silver/SSE compared to measurements with a similar cell operating with a saturated mercury/MSE. The difference of *E*°
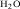
(SSE, H_2_O) and *E*°
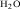
(MSE, H_2_O) is given as 42 mV.^[^ ^38^ ^]^ Published data between approximately 0.5 and 63 wt% H_2_SO_4_ were included (*Harned and Hamer^[^ ^40^ ^]^; **Beck et al.^[^ ^39^ ^]^). b) Visual representation of the network of half‐cells of cells II obtained with the ILSB method. A circle represents a half‐cell, and the blue digit indicates the wt% content of H_2_SO_4_ in water. Two half‐cells connected by an arrow represent one implementation of cell II. The direction of the arrow indicates the cell reaction of cell II (Equation M2), i.e., H^+^(solv, S_1_) → H^+^(solv, S_2_), i.e., the transfer of H^+^ from the right half‐cell to the left half‐cell. The numbers in the ellipses superimposed on the arrows are the measured potential differences of the respective implementation of cell II, *E*_II_, in mV. The optimized *E*_II,opt_ values, obtained from the least squares method, as well as the resulting ${\mathrm{pH}}_{\mathrm{abs}}^{H_{2}O}$ values are listed in Table S5; they are given with respect to S5 = 5% H_2_SO_4_ in water (${\mathrm{pH}}_{H_{2}O}$ = ${\mathrm{pH}}_{\mathrm{abs}}^{H_{2}O}$ = 0.3) as the reference solvent. The *σ*‐value (measurement uncertainty) for the entire network of 42 measurements over 12 half‐cells was found to be about 60 mV or 1 pH unit.

Measurements between 1 and 100 wt% sulfuric acid were done, in which the 1 wt% value undisputedly corresponds to ${\mathrm{pH}}_{H_{2}O}$ = ${\mathrm{pH}}_{\mathrm{abs}}^{H_{2}O}$ = 1. The SSE‐values *E*_I_ measured with cell I are given in Table S4 and are plotted in Figure 3a together with orienting values measured with a mercurous sulfate electrode (MSE) instead of an SSE. The two cell voltages run in parallel, increase with acid concentration, and should be separated theoretically by 42 mV, but the difference is slightly less.^[^ ^38^ ^]^ In addition, the measurements by Beck et al.^[^ ^39^ ^]^ and Harned and Hamer^[^ ^40^ ^]^ are also included and agree very well with ours in the concentration range assessed (below 70 wt%). At acid contents higher than 70 wt%, the SSE‐ (and MSE‐)curve clearly shows the steep increase of the measured potential. Note that around 73 to 80 wt%, the sulfuric acid also has anomalies of physical properties like viscosity and conductivity (see below); in this range the measured potentials were also the least stable, and we estimate an uncertainty around ±2 pH units (see discussion).

In addition, the ILSB‐measured potential difference values *E*_II_ of thirty implementations of cell II shown in Figure 3b were measured within one network and then analyzed with the least square method; the *σ*‐value for the entire network of 42 measurements over 12 half‐cells was found to be about 60 mV or 1 pH unit. This resulted in optimized *E*_II,opt_ values for each mixture that are included in Table S5.

#### Results for Pure Sulfuric Acid

In 100 wt% sulfuric acid, undisputedly the most challenging measurement series, the difference Δ*E* in the measured potentials between 5 and 100 wt% H_2_SO_4_ shown in Figure 3 as well as Tables S4 and S5 are proposed to originate from changes in the protochemical potential. The measured Δ*E* values of 1372 mV (SSE) and 1552 mV (ILSB) correspond to a chemical potential change of 23 or 26 orders of magnitude, with 59 mV representing an order of magnitude. Given that we start at about ${\mathrm{pH}}_{\mathrm{abs}}^{H_{2}O}$ ≈ 0 and that the potential changes are caused by the proton, for pure sulfuric acid the simple approximation of the Hammett value *H*_0_ approximating negative aqueous pH values appears erroneous. The SSE‐value *E*_I_ measured in 100% H_2_SO_4_ with cell I was converted with Equations (5) and (M1) (see Table S4) into the ${\mathrm{pH}}_{\mathrm{abs}}^{H_{2}O}$ value (−22.9 ± 2.0). From the potential difference values of 30 implementations of cell II and the ILSB‐method that were optimized with the least squares method within one network, we obtained the ${\mathrm{pH}}_{\mathrm{abs}}^{H_{2}O}$ value for 100% H_2_SO_4_ as −25.9 ± 2.0. Both agree within the uncertainties and, hence, in the discussion, we will use their average value without considering decimals, i.e., ${\mathrm{pH}}_{\mathrm{abs}}^{H_{2}O}$(100 wt% H_2_SO_4_) = −24.

### QME‐Results

**Figure 4:**
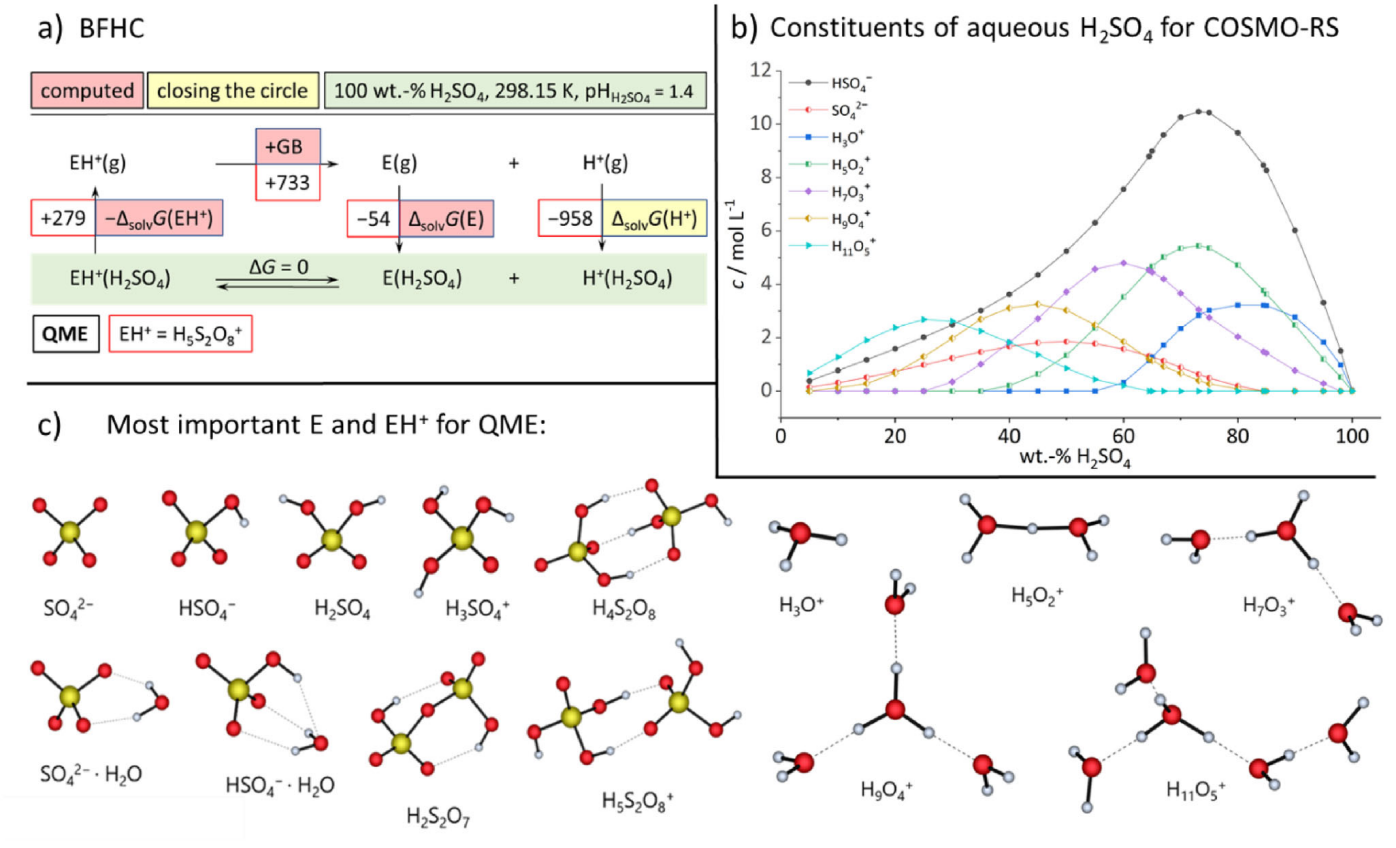
Principle behind the QME method. a) BFHC used to assess the Gibbs energy of solvation of the proton in sulfuric acid, including the exemplary worked example of H_5_S_2_O_8_^+^ as EH^+^. b) Distribution of the constituent ions of H_2_O‐H_2_SO_4_ mixtures between 5 and 100 wt% H_2_SO_4_ for calculating the Gibbs solvation energy of the proton with the COSMO RS model and **QME**. Their concentrations are based on experimental Raman data,^[^ ^41^ ^]^ but were slightly adjusted (Supporting Information). Note that at 73 wt% (*x*(H_2_O)/*x*(H_2_SO_4_) = 2) the concentrations of both the HSO_4_^−^ and the H_5_O_2_^+^ ions are maximal. c) Most important structures of the particles E and EH^+^ used within the **QME**‐method for the COSMO RS calculation.

Finally, we describe a method that enforced us to gain insights into the different species present in sulfuric acid and its aqueous mixtures. With this QME method, the *quantum chemically computed energetics of solvated entities*, we have computed the chemical potential of the proton by using the BFHC in Figure 4a and modeling the realistic composition of either aqueous or, separately, pure sulfuric acid from all chemical entities E and their protonated forms EH^+^ that constitute the acid as shown in Figure 4b,c. Hence, no additional reference substances were included.

**Figure 5:**
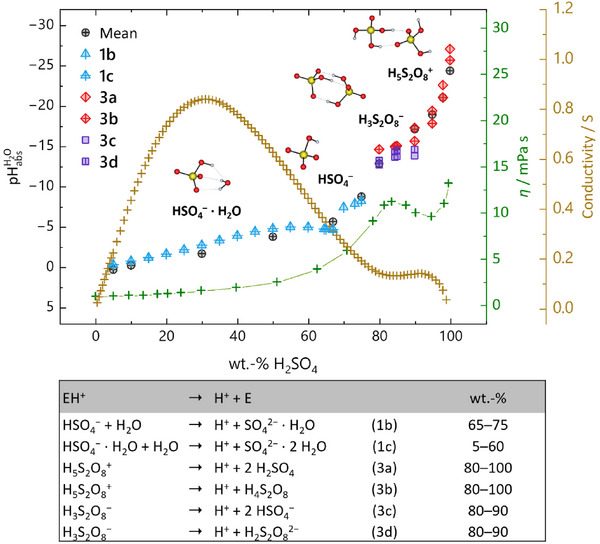
**Top)** Comparison of experimental and computed pH acidity (left axis) and superimposed to the experimental kinematic viscosities at 25 °C^[^ ^49^ ^]^ (green “+” and green axis, right) and conductivities at 26.7 °C (Note that the author gives the conductivity values in reciprocal Ohm, although he probably means S cm^−1^. We have nevertheless used the original entries. (brown “+” and brown axis, right). In addition, representations of the protonated entities EH^+^ and their underlying equilibria 1–3 relevant for the chemical potential of the proton are included in the table at the **bottom**.

The modeling of aqueous sulfuric acid S started from the experimentally known concentrations of HSO_4_^−^ and SO_4_^2−^ ions as determined by Raman spectroscopy.^[^ ^41^ ^]^ Then, the concentrations of the stoichiometrically necessary amounts of protonated water clusters were adjusted to obey electroneutrality of the mixtures. Thus, the protonated water clusters (H(H_2_O)_n_)^+^, H_2_O, H_2_SO_4_, HSO_4_^−^, SO_4_^2−^ exist in the calculated aqueous sulfuric mixtures S at finite concentration; all other entities E/EH^+^ perhaps present were assessed at infinite dilution (Figure 4b,c). The chemical potentials of the proton for each concentration are given in Table 2 and in Figure 5; for full details, see Methods and Section S2.3.

**Table 2:** Results of the QME method with different protonated entities EH^+^ in 100 wt% H_2_SO_4_. All calculated energies required to complete the thermodynamic cycle QME in Figure 4 are given. The solvation energy as modeled with COSMO‐RS of species EH^+^, Δ_solv_ *G*(EH^+^), the solvation energy of E, Δ_solv_ *G*(E), the GB (Δ_g_ *G*°) at the DLPNO‐CCSD(T)/CBS level of theory, the resulting proton solvation Gibbs energy Δ_solv_ *G*(H^+^, H_2_SO_4_) (in kJ mol^−1^), and ${\mathrm{pH}}_{\mathrm{abs}}^{H_{2}O}$ values (Equations 2 and 5) and their median.

EH^+^ ⇌ H^+^+ B	Δ_solv_ *G*(EH^+^) (kJ mol^−1^)	Δ_solv_ *G*(E) (kJ mol^−1^)	GB (kJ mol^−1^)	Δ_solv_ *G*(H^+^, H_2_SO_4_) (kJ mol^−1^)	${\mathrm{pH}}_{\mathrm{abs}}^{H_{2}O}$
HSO_4_^−^ ⇌ H^+^ + SO_4_^2−^	−310.3	−1216.0	1860.1	−954.5	−26.3
H_3_SO_4_^+^ ⇌ H^+^ + H_2_SO_4_	−296.1	−41.6	684.7	−939.2	−29.0
H_5_S_2_O_8_^+^ ⇌ H^+^ + H_4_S_2_O_8_	−279.3	−54.1	732.7	−957.9	−25.7
H_5_S_2_O_8_^+^ ⇌ H^+^ + 2 H_2_SO_4_	−279.3	−83.2	753.8	−949.9	−27.1
H_4_S_2_O_8_ ⇌ H^+^ + H_3_S_2_O_8_^−^	−54.1	−230.2	1154.5	−978.4	−22.1
H_2_S_2_O_7_ ⇌ H^+^ + HS_2_O_7_^−^	−36.6	−220.7	1182.9	−988.1	−20.4
H_3_O^+^ ⇌ H^+^ + H_2_O	−337.7	−32.1	656.9	−962.5	−24.9
	Average				−25.1

By contrast, the E/EH^+^ constituents considered for 100 wt% H_2_SO_4_ were those that form in agreement with the experimental constant of autoprotolysis $pK_{\mathrm{ap},H_{2}{\mathrm{SO}}_{4}}$ = 3.10 and of self‐dehydration $pK_{\mathrm{ip},H_{2}{\mathrm{SO}}_{4}}$ = 3.85;^[^ ^32^ ^]^ they are collected in Table 2. The latter constants lead to a ${\mathrm{pH}}_{H_{2}{\mathrm{SO}}_{4}}$ value of 1.4. All species besides the ions resulting from the two dissociation equilibria and H_2_SO_4_ exist only at infinite dilution in the mixture.

Using this approach and focusing on 100 wt% H_2_SO_4_, seven QME ${\mathrm{pH}}_{\mathrm{abs}}^{H_{2}O}$ values were computed for pure sulfuric acid that range from −20.4 to −29.0 and yield an average ${\mathrm{pH}}_{\mathrm{abs}}^{H_{2}O}$ value of −25.1. All necessary data to close the BFHC is collected in Table 2. Note that the amounts of the individual E/EH^+^ entities were adjusted in the COSMO‐RS mix to mirror the expected concentrations from the law of mass action. Hence, the Gibbs solvation energies given are non‐standard.

Although all four presented methods are based on completely different assumptions, the median of all—experimental, semi‐empirical, or computational—${\mathrm{pH}}_{\mathrm{abs}}^{H_{2}O}$ values of pure sulfuric acid of −24 agrees within the uncertainties of the individual methods of about ±2 pH values or the equivalent of ±11.4 kJ mol^−1^ in chemical potential.

## Discussion

Prior to discussing implications and the relation between pH and *H*_0_, we analyze and discuss if the electrochemical potential changes are caused by changes in the proton activity or if other effects need to be considered.

### Analysis of the Origin of the Observed Potential Changes

#### Consideration of Possible Side Reactions

In addition to the redox pair H^+^/H_2_, other redox pairs causing mixed potentials at the H_2_‐electrode could in principle be relevant in sulfuric acid, of which the redox couple SO_4_^2−^/SO_2_ (*E*°
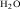
(H_2_O) = 0.158 V)^[^ ^42^ ^]^ has been indicated as dominant. Yet, slow electrode kinetics with an exchange current density of 10^−11^ A cm^−2^ has been reported for the couple SO_4_^2−^/SO_2_ in 44 wt% H_2_SO_4_ at 50 °C.^[^ ^43^ ^]^ The authors added that “*at acid concentrations higher than 50%, the rate falls dramatically*.” By contrast, the exchange current density of the H^+^/H_2_ redox couple at platinum reaches 10^−3^ A cm^−2^ in appr. 10 wt% H_2_SO_4_ already at 25 °C.^[^ ^44^ ^]^ Given that the rates of the individual electrode reactions are very different, and assuming that the hydrogen reaction is at least 6 orders of magnitude faster, then the mixed potential lies very close to the here relevant equilibrium potential of the faster H^+^/H_2_ redox couple. Further reduction reactions of SO_4_^2−^ are reported to occur only as subsequent reductions of SO_2_ or HSO_3_^−^, respectively, and are even more sluggish.^[^ ^45^, ^46^ ^]^ This also holds true for the oxidation of platinum instead of hydrogen.^[^ ^47^ ^]^ The reported anodic dissolution of platinum exclusively occurs after polarizing the platinum electrode cathodically in 14–18 mol L^−1^ H_2_SO_4_.^[^ ^48^ ^]^ Therefore, if used in the arrangement of cells I and II and operated in a currentless mode, platinum dissolution is not expected.

#### Experimental Validation

To prove this point and independently study potential reduction reactions, we used NMR and Raman spectroscopy to analyze samples of concentrated sulfuric acid as well as its suspensions with platinum black with and without a hydrogen atmosphere stored in NMR tubes safely sealed with a J. Young valve. After a measurement series of 8 days, the NMR and Raman spectra did not change with reference to the neat concentrated sulfuric acid, and we did not observe any evidence for any reduction products. Thus, with this evaluation (Section S1.4), the formation of any mixed potential that deviates significantly from the equilibrium potential of the H^+^/H_2_ system is unlikely, and we suggest the measured potential changes to be dominated by the proton.

### Acidity Progression and Speciation in H_2_O‐H_2_SO_4_ Mixtures between 5 and 100 wt% Sulfuric Acid

Figure 5 summarizes the mean of the SSE and ILSB measurements as well as the QME method (Mix 3; see Supporting Information), along with published viscosity^[^ ^49^ ^]^ and conductivity data.^[^ ^50^ ^]^ The QMB method cannot be applied to the H_2_O‐H_2_SO_4_ mixtures. None of the thermodynamic cycles describes the experimental results across the entire concentration range of aqueous sulfuric acid, thus, the approximate concentration range where each cycle applies is included in Figure 5. For acid concentrations below approximately 73 wt%, the QME calculations suggest the somewhat counterintuitive explanation that the protochemical potential is determined by the HSO_4_^−^ anion, which is the main carrier of the proton in such systems (cycles 1b and 1c of Figure 5) instead of the expected protonated water clusters H(H_2_O)*_n_* ^+^ (see Supporting Information S2.3). In the range between about 70 and 80 wt%, i.e., in the range that covers the ratio of two water molecules per sulfuric acid molecule, we were not able to identify any single EH^+^ species that reflects the acidity progression alone with an uncertainty of less than 2.7 pH units. Indeed, in this range also the measurements show an increased uncertainty. Yet, as evident from the published experimental viscosity^[^ ^49^ ^]^ and conductivity^[^ ^50^ ^]^ data also included with Figure 5a, one realizes that this must stem from substantial structural reorganization of the sulfuric acid components at those concentrations. Nevertheless, at concentrations that correspond to less than two water molecules per acid molecule, the effective shielding of charged species appears to be reduced, so that the protochemical potential rises drastically. Concomitantly, the potential‐determining species changes above 80 wt% from HSO_4_^−^ to its adduct with sulfuric acid H_3_S_2_O_8_^−^ (3c and 3d), which correlates with the viscosity minimum at 83 wt% because of its compact size in a framework where the intermolecular bonding situation is overall very similar. With a further increase in the acid concentration, the potential determining species transitions to H_5_S_2_O_8_^+^ (3a and 3b), which causes an increase in viscosity.

### Quantification of the Differences Between ${\text{pH}}_{\text{abs}}^{H_{2}O}$ and *H*_0_ in Pure H_2_SO_4_

**Figure 6:**
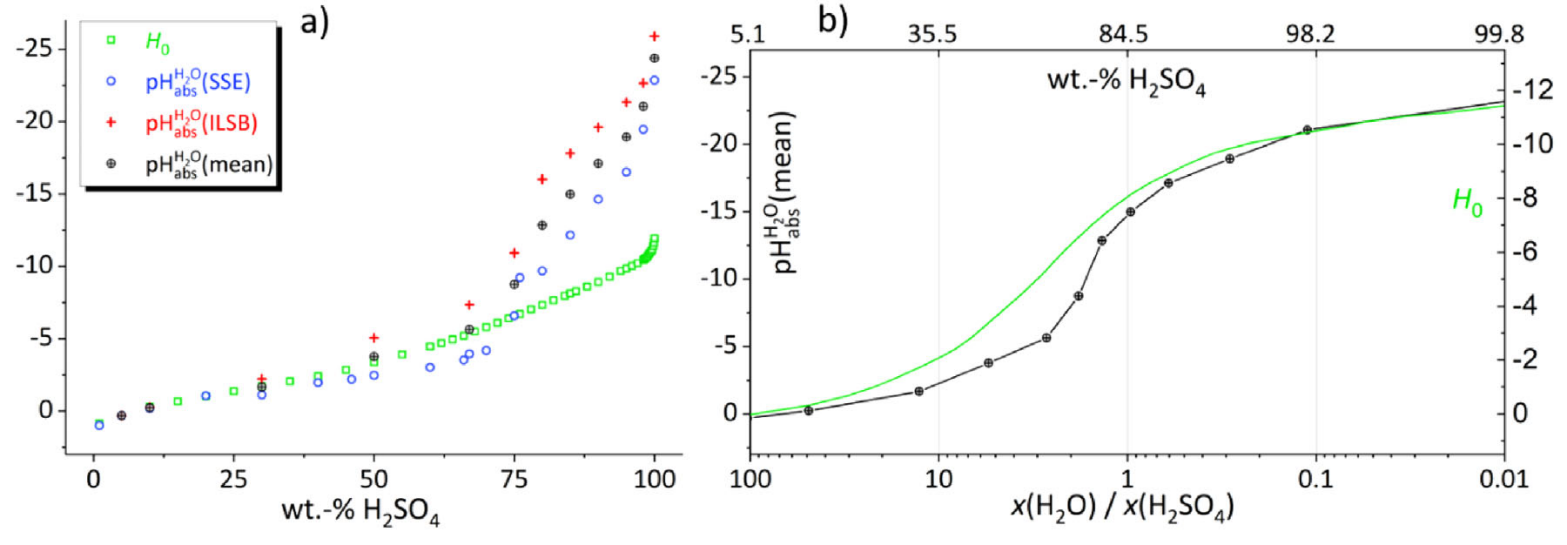
Development of *H*_0_ (green) and ${\mathrm{pH}}_{\mathrm{abs}}^{H_{2}O}$ as obtained with the SSE (blue), the ILSB (red), and their mean (black) as a function of the content of H_2_SO_4_ in H_2_O in two representations. The *H*_0_ values are combined data from different groups due to refinements of measurements, particularly in the highly acidic region: 1–55 wt% Paul and Long;^[^ ^51^ ^]^ 60–97 wt% Jorgenson and Hartter;^[^ ^52^ ^]^ 98–100 wt% Gillespie et al.^[^ ^33^ ^]^ a) Acidity versus wt% H_2_SO_4_. At low acid concentrations, pH and *H*_0_ acidity proceed—as expected—essentially congruently until they begin to diverge at about 30 wt% or 4 mol L^−1^. The subsequent ${\mathrm{pH}}_{\mathrm{abs}}^{H_{2}O}$(mean) values agree within our uncertainties to the *H*_0_ values. Yet, at about 75 wt%, both ${\mathrm{pH}}_{\mathrm{abs}}^{H_{2}O}$ values indicate an enormous increase in acidity, so pronounced that they clearly exceed the also increasing *H*_0_ acidity. Afterwards, the gradient of both decelerates slightly but remains significantly steeper than that of *H*_0_. Finally, all acidities increase significantly again above about 98 wt% to their final values at 100% H_2_SO_4_ that diverge for *H*_0_ = −11.9 and ${\mathrm{pH}}_{\mathrm{abs}}^{H_{2}O}$(mean) = −24. b) Acidity versus number of H_2_O molecules per H_2_SO_4_ molecule, *x*(H_2_O)/*x*(H_2_SO_4_), in logarithmic form with its own scale on the right for *H*_0_. The top *x*‐axis also shows the respective wt% H_2_SO_4_ for better comprehensibility (note that in this representation 100 wt% H_2_SO_4_ cannot be plotted). At *x*(H_2_O)/*x*(H_2_SO_4_) ≈ 2 (corresponding to 73 wt% H_2_SO_4_) the curves show an inflection point, i.e., the pH as well as the *H*_0_ acidity increases drastically.

Figure 6 shows, with two representations differentiating in the definition of their *x*‐axis, the results of our experimental ${\mathrm{pH}}_{\mathrm{abs}}^{H_{2}O}$ determinations together with *H*_0_ values in the entire H_2_O‐H_2_SO_4_ mixing range. We note from this figure, most evident from the position of the inflection point in Figure 6b, that the curves correspond qualitatively. This implies that the pH acidity as well as the *H*_0_ acidity show a similar, distinctive characteristic. Apparently, this common characteristic is induced by the protochemical potential, i.e., a constituent of all methods considered.

The distinction between the pH and *H*_0_ acidity is the inclusion of the medium effect of the indicator base and its protonated form into the former (cf. Equations 1 or 2 with Equations 3 or 3b). From Equations 4 and 5, and considering _s_ *f* as unity, since the indicator concentrations for the *H*_0_ determination were exceedingly small, we obtained from the ${\mathrm{pH}}_{\mathrm{abs}}^{H_{2}O}$ and *H*_0_ data the difference of the Gibbs transfer energies of the indicator base B and its protonated form BH^+^ (for the moment formulated for pure H_2_SO_4_, but this applies to all H_2_O‐H_2_SO_4_ mixtures S), ΔΔ_tr_ *G*°(B − BH^+^, H_2_O→ H_2_SO_4_), Equation (6a).

(6a)

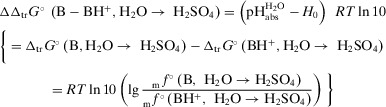




Since no base exists, which is in a sufficiently well measurable equilibrium with its protonated form in both water and sulfuric acid—the reason, why Hammett et al. had to use different bases B, C, D, etc. suitable for the respective mixtures—Equation (6a) has to be modified slightly. However, this does not interfere with the argumentation. For the purpose of simplicity, let us first assume that such a fictitious base B^fict^ exists. In 100 wt% H_2_SO_4_ the values of ${\mathrm{pH}}_{\mathrm{abs}}^{H_{2}O}$ = −24_mean_ and *H*_0_ = −11.9 lead to ΔΔ_tr_ *G*°(B^fict^ − B^fict^H^+^, H_2_O→ H_2_SO_4_) = −69.1 kJ mol^−1^. Yet, instead of analyzing the transfer of B^fict^ and B^fict^H^+^, i.e., Equations (7a) and (7b),

(7a) $$\begin{array}{cc} & \Delta_{\mathrm{tr}}G^{\circ }\left(B^{\mathrm{fict}},H_{2}O\to H_{2}{\text{SO}}_{4}\right)=\\ & \Delta_{\mathrm{solv}}G^{\circ }\left(B^{\mathrm{fict}},H_{2}{\text{SO}}_{4}\right)-\Delta_{\mathrm{solv}}G^{\circ }\left(B^{\mathrm{fict}},H_{2}O\right) \end{array}$$

(7b) $$\begin{array}{cc} & \Delta_{\mathrm{tr}}G^{\circ }\left(B^{\mathrm{fict}}H^{+},H_{2}O\to H_{2}{\text{SO}}_{4}\right)=\\ & \Delta_{\mathrm{solv}}G^{\circ }\left(B^{\mathrm{fict}}H^{+},H_{2}{\text{SO}}_{4}\right)-\Delta_{\mathrm{solv}}G^{\circ }\left(B^{\mathrm{fict}}H^{+},H_{2}O\right) \end{array}$$

the real bases for the *H*_0_ determination in water, B^W^, and for the *H*_0_ determination in sulfuric acid, B^SA^, have to be analyzed with the following processes, Equations (7c) and (7d):

(7c) $$\begin{array}{cc} & \Delta G^{\circ }\left(B^{W},H_{2}O\to B^{\mathrm{SA}},H_{2}{\text{SO}}_{4}\right)=\\ & \Delta_{\mathrm{solv}}G^{\circ }\left(B^{\mathrm{SA}},H_{2}{\text{SO}}_{4}\right)-\Delta_{\mathrm{solv}}G^{\circ }\left(B^{W},H_{2}O\right) \end{array}$$

(7d) $$\begin{array}{cc} & \Delta G^{\circ }\left(B^{W}H^{+},H_{2}O\to B^{\mathrm{SA}}H^{+},H_{2}{\text{SO}}_{4}\right)=\\ & \Delta_{\mathrm{solv}}G^{\circ }\left(B^{\mathrm{SA}}H^{+},H_{2}{\text{SO}}_{4}\right)-\Delta_{\mathrm{solv}}G^{\circ }\left(B^{W}H^{+},H_{2}O\right) \end{array}$$

The difference between these two magnitudes inserted into Equation (6a) provides the modified Equation (6b).

(6b)

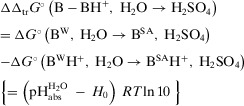




**Figure 7:**
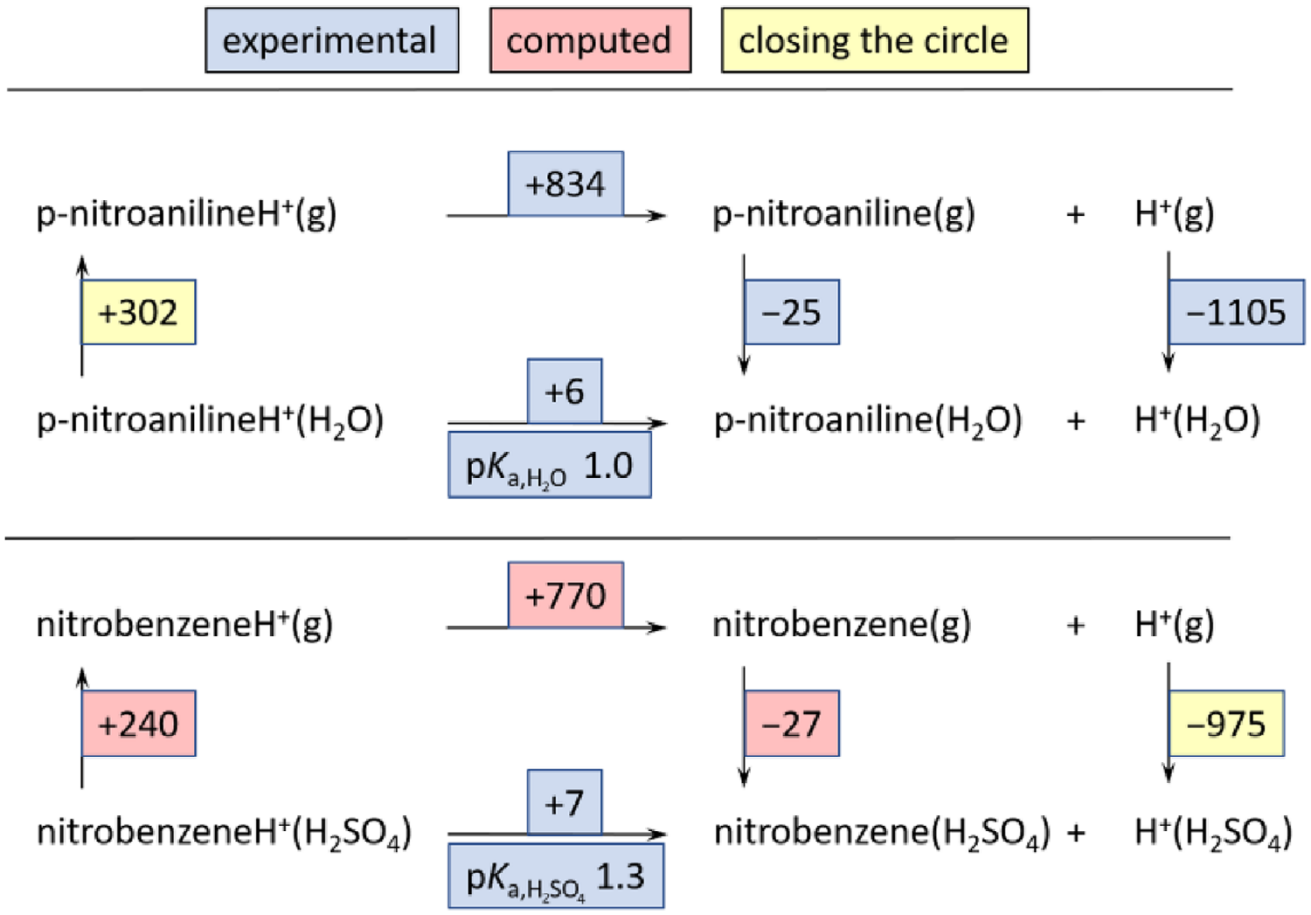
Thermodynamic cycles to obtain Gibbs solvation energies utilized in Equation (6b), exemplified for B^W^ = p‐nitroaniline and B^SA^ = nitrobenzene, the couple that Gillespie et al. explicitly used to establish the Hammett‐value of −11.9 for pure sulfuric acid. All values in kJ mol^−1^.

As an example, we take B^W^ = p‐nitroaniline and B^SA^ = nitrobenzene, the couple that Gillespie et al. explicitly used to establish the Hammett value of −11.9 for pure sulfuric acid. Figure 7 shows the thermodynamic cycles to illustrate how the four relevant Gibbs solvation energies (experimental or computed with COSMO‐RS) are related. These values inserted in Equation (6b) result in ΔΔ_tr_ *G*°(B − BH^+^, H_2_O→ H_2_SO_4_) = −64.4 kJ mol^−1^; the median of 8 bases B^SA^ with B^W^ = p‐nitroaniline is −62.9 kJ mol^−1^ (see Table S8 and cf. Table 1) and corresponds to the difference between measured ${\mathrm{pH}}_{\mathrm{abs}}^{H_{2}O}$ and *H*_0_ data (Equations 6a and 6b). With the above experimentally derived value of (${\mathrm{pH}}_{\mathrm{abs}}^{H_{2}O}$ − *H*_0_) *RT* ln 10 = −69.1 kJ mol^−1^, a small difference between computations and experiments of about 1 pH unit follows.

From the above and Equations (6) and (7) follows Equation (8) to obtain ${\mathrm{pH}}_{\mathrm{abs}}^{H_{2}O}$ values from *H*_0_ values in pure sulfuric acid. Let us denote the second term of Equation (8) as “base transfer term.”

(8) $$\begin{array}{ccc} {\mathrm{pH}}_{\mathrm{abs}}^{H_{2}O} & = & H_{0}+\frac{\Delta \Delta_{\mathrm{tr}}G^{\circ }\left(B-{\mathrm{BH}}^{+},\;H_{2}O\to H_{2}{\mathrm{SO}}_{4}\right)}{\mathrm{RT}\ln10}\\ & \approx & H_{0}-12 \end{array}$$

**Figure 8:**
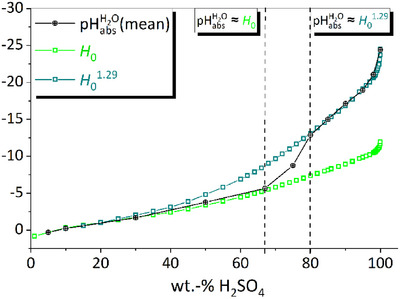
Rule of thumb between ${\mathrm{pH}}_{\mathrm{abs}}^{H_{2}O}$ and *H*_0_ using ${\mathrm{pH}}_{\mathrm{abs}}^{H_{2}O}$ = −(|*H*_0_|^1.29^).

This approach to evaluate the difference between S_0_ = 0 wt% and S_100_ = 100 wt% acid in principle also applies to the differences between all S. In order to provide a relation between ${\;\mathrm{pH}}_{\mathrm{abs}}^{H_{2}O}$ and *H*_0_ for H_2_O‐H_2_SO_4_ mixtures, Figure 8 is helpful.

In Figure 8 the mean of our experimental ${\mathrm{pH}}_{\mathrm{abs}}^{H_{2}O}$ values, the *H*_0_ values, and modified *H*_0_ values are shown as function of the H_2_SO_4_ content. In view of all our assumptions, we are astounded at the extent to which the mean value of our experimental methods coincides with the *H*_0_ values. This is true for H_2_O‐H_2_SO_4_ mixtures up to about 70 wt% H_2_SO_4_. In this case, the (protonated) indicator bases are likely to be solvated preferentially by H_2_O molecules, and therefore the base transfer term for the bases (with H_2_O and S*_x_* instead of H_2_SO_4_, *x* ≲ 70 wt% H_2_SO_4_) is negligible.

Protons are also predominantly solvated by water molecules in this region. However, if less than approximately two H_2_O molecules are available per H_2_SO_4_ molecule, pH acidity and *H*_0_ acidity begin to diverge: The chemical environment of a proton—which is not bound to an indicator base (!)—changes completely when the solvation changes. If the proton is bound to a base, its next chemical environment only changes partially when the solvation changes, because the base–H^+^ bond is not broken. In other words, the chemical potential of the proton changes to a greater extent than the chemical potential of the proton, i.e., bound to a base. Therefore, in the *H*_0_ acidity scale, the acidity appears lower than in the pH acidity scale, and we observe here exactly what has been aptly paraphrased by Bates and Schwarzenbach:^[^ ^53^ ^]^

“*The Acidity Function (H_0_) means, as it were, the difference in acidity between water and the non‐aqueous sample solution as sensed by an individual indicator*.”

And this is the meaning of the base transfer term. The latter can no longer be neglected, as both the unprotonated base and the protonated base are subject to a change in solvation, simply because there are not enough water molecules to maintain the preferential solvation.

In the range higher than about 80 wt%, this interplay of ratios around the chemical potentials of the protons, the protonated base, and the unprotonated base continues, and we state (the empirical) Equation (9) that can be used for concentrated sulfuric acid (> 80 wt% H_2_SO_4_) as a rule of thumb to convert *H*_0_ values into ${\mathrm{pH}}_{\mathrm{abs}}^{H_{2}O}$ values.

(9) $${\mathrm{pH}}_{\mathrm{abs}}^{H_{2}O}=-\left({\left|H_{0}\right|}^{1.29}\right)$$

### General Method to Convert *H*_0_ Values into ${\text{pH}}_{\text{abs}}^{H_{2}O}$ Values

Note that, even if the same set of indicators would have been used to determine the *H*_0_ values, e.g., in perchloric acid (HClO_4_), the implicit assumption that ΔΔ_tr_ *G*°(B − BH^+^, H_2_O→ S) = ΔΔ_tr_ *G*°(B − BH^+^, H_2_O→ P) (P are the H_2_O‐HClO_4_ mixtures) would have to be made when comparing the individual values directly with each other. This assumption can hardly be justified, because it does not consider the different solvation of the bases and protonated bases in pure H_2_SO_4_ or HClO_4_ resulting at least from the different permittivities of the individual systems. As a consequence, this also implies that the ${\mathrm{pH}}_{\mathrm{abs}}^{H_{2}O}$ values assigned to the *H*_0_ values in this work cannot simply be transferred to *H*_0_ values of other acidic systems (of course, this is also true for the above‐provided rule of thumb). *H*_0_ values can only be converted into ${\mathrm{pH}}_{\mathrm{abs}}^{H_{2}O}$ values, if the Gibbs transfer energies of the protonated and unprotonated bases are known.

Above we noted that for the calculation of the ${\mathrm{pH}}_{\mathrm{abs}}^{H_{2}O}$ value using the QMB‐method, one does not need any detailed information of the individual solvation of the proton. Further, we showed that also simpler quantum chemical continuum solvation models like CPCM (and related ones) should be sufficient to describe the differences of the Gibbs solvation energies of B/BH^+^ pairs in water and sulfuric acid, only based on the permittivity of the medium. And, if this holds for the complicated case of pure sulfuric acid with its pronounced autoprotolysis (p*K*_ap_ = 3.1) and, therefore, high concentrations of ions influencing the individual solvation, this should also be applicable to many other pure acids and eventually even acid‐mixtures for which an individual p*K*_a_‐value of BH^+^ in the respective medium is known. Pleasingly, the calculation of the ${\mathrm{pH}}_{\mathrm{abs}}^{H_{2}O}$ value using the QMB‐method, but only the simple CPCM model and not the COSMO RS method including all ions present at equilibrium concentration, gave average values of −22.5_CPCM_ versus −22.1_COSMO RS_, and also the individually for each base couple B/BH^+^ calculated values match within less than 1.2 pH units (Table 1). Note that a considerable amount of error cancellation occurs, since the individual Gibbs solvation energy values calculated differ by up to 22.4 kJ mol^−1^ between the methods for the same particle. Yet, in the cycle those differences cancel. As expected, the absolute calculated values with the simpler CPCM model might be erroneous, but if applied to both sides of the BFHC cycle, the relative differences come out rather well. Therefore, we do suggest that the QMB‐method might present a rather generally applicable method to estimate the ${\mathrm{pH}}_{\mathrm{abs}}^{H_{2}O}$ values from an evaluation of the Gibbs solvation energies of the B/BH^+^ systems with known p*K*_a_‐value of BH^+^ in pure acids and maybe also acidic (solvent) mixtures. The most rigorous method, however, is the experimental determination of ${\mathrm{pH}}_{\mathrm{abs}}^{H_{2}O}$ values using electrochemical cells equipped with hydrogen electrodes as described above.

### Definition of Superacidity Based on the Protochemical Potential

From the above discussion we arrive at the remarkably consistent finding that the activity of the proton in pure sulfuric acid is over thirty orders of magnitude higher than its activity in pure neutral water. In terms of ${\mathrm{pH}}_{\mathrm{abs}}^{H_{2}O}$ acidity this corresponds to an average of 31 units. We present, in accordance with Gillespie´s definition, from Figures 5 and 6 a quantitative and measurable definition of superacidity:

*Any Brønsted superacid or Brønsted superacidic environment includes protons with a protochemical potential in this environment that lies higher than the protochemical potential of pure sulfuric acid*.

We suggest this level to lie at a ${\mathrm{pH}}_{\mathrm{abs}}^{H_{2}O}$ value of −24. Note that this definition may include a liquid, gaseous, or solid environment, hence it could also address gaseous or solid superacidic systems like highly acidic zeolites. In addition, when (erroneously) taking the Hammett *H*_0_ value as a continuation of the pH scale to the realm of negative pH values, ${\mathrm{pH}}_{\mathrm{abs}}^{H_{2}O}$ = −24 indicates a by 12 orders of magnitude higher acidity than anticipated according to this widely used, but wrong interpretation of the *H*_0_ value of −11.9 of pure sulfuric acid.

## Conclusion

We used two experimental, one semi‐empirical, and one theoretical method to quantify the protochemical potential of pure sulfuric acid and in sulfuric acid‐water mixtures within the ${\mathrm{pH}}_{\mathrm{abs}}^{H_{2}O}$ scale (applicable to all media, aligned to the aqueous scale ${\mathrm{pH}}_{H_{2}O}$). Each method is based on different assumptions, which is why the individual experimental results for the acidity level of pure sulfuric acid differ by 3 pH units (−22.9 and −25.9), and the 14 computed ${\mathrm{pH}}_{\mathrm{abs}}^{H_{2}O}$ values for pure sulfuric acid scatter within −20.4 and −29.0. However, all values yield a median ${\mathrm{pH}}_{\mathrm{abs}}^{H_{2}O}$ value of −24 and hence indicate a by 12 orders of magnitude higher acidity than anticipated according to the widely used (but erroneous) interpretation of the Hammett *H*_0_ value of −11.9. The reason is that the *H*_0_ acidity function includes, besides the protochemical potential, a term that accounts for the differences of Gibbs solvation energies of the indicator bases and their protonated forms used to determine the individual *H*_0_ values. The comparison with our results allows to quantify this term for pure sulfuric acid as on average −68.5 kJ mol^−1^, depending on the indicator base used. This value corresponds to an equivalent of 12 pH units or 12 orders of magnitude, respectively, that was hitherto neglected. The *H*_0_ acidity, therefore, does not indicate the acidity in the Brønsted sense. Ignoring this aspect, hence considering the *H*_0_ acidity function as a continuation of the aqueous ${\mathrm{pH}}_{H_{2}O}$ scale, would lead to an underestimation of the acidity of pure sulfuric acid by the median of 12 pH units. The conversion of *H*_0_ values into ${\mathrm{pH}}_{\mathrm{abs}}^{H_{2}O}$ values or vice versa is not straightforward. In the range below 70 wt% H_2_SO_4_ we state that ${\mathrm{pH}}_{\mathrm{abs}}^{H_{2}O}$ ≈ *H*_0_, at concentrations higher than 80 wt% H_2_SO_4_ we find that ${\mathrm{pH}}_{\mathrm{abs}}^{H_{2}O}$ ≈ −(|*H*_0_|^1.29^), and in between, we cannot provide a sharp relation (cf. Figure 8). As a rule of thumb, it is only an approximation, and it only applies strictly to the system considered here, i.e., sulfuric acid with the indicator bases, with which the *H*_0_ values were determined. We **strongly discourage** using this rule of thumb to convert, e.g., the *H*_0_ value of the HF‐SbF_5_ system^[^ ^54^ ^]^ of −23 (which is not even comparable to the *H*_0_ value of pure H_2_SO_4_) into the value ${\mathrm{pH}}_{\mathrm{abs}}^{H_{2}O}$ −57. We expect that the difference ${\mathrm{pH}}_{\mathrm{abs}}^{H_{2}O}$ − *H*_0_ of other acids is similar to the sulfuric acid case, i.e., the protochemical potential as true acidity (in the Brønsted sense) is much higher than suggested by *H*_0_, or, in other words, that the base transfer term is also negative. However, to obtain a definite value and an own rule of thumb, each acidic system has to be investigated in terms of its protochemical potential, as shown above. Alternatively, but likely less accurately, the QMB method allows the base transfer term to be determined by computation.

The ${\mathrm{pH}}_{\mathrm{abs}}^{H_{2}O}$ value displays the protochemical potential of the medium; thus, it can be converted into the $E_{\mathrm{abs}}^{H_{2}O}$ value with Equation (5). According to this work, in pure sulfuric acid the hydrogen electrode therefore has the value $E_{\mathrm{abs}}^{H_{2}O}$(H_2_/H^+^, 100% H_2_SO_4_) = 1.42 V. At standard conditions—, i.e., $a_{H_{2}{\mathrm{SO}}_{4}}$(H^+^, H_2_SO_4_) = 1 or ${\mathrm{pH}}_{H_{2}{\mathrm{SO}}_{4}}$ = 0, respectively—the potential of the Standard Hydrogen Electrode (SHE) in sulfuric acid SHE(H_2_SO_4_) has the value $E_{\mathrm{abs}}^{H_{2}O}$(SHE, H_2_SO_4_) = 1.50 V.

Interestingly, the QME‐method suggests that the chemical entity determining the protochemical potential up to about 73 wt% sulfuric acid in water is not any of the expected (cationic) protonated water clusters H(H_2_O)_n_^+^, but is the anionic HSO_4_^−^ entity. At higher concentrations and if the mixture consists of only two water molecules per sulfuric acid molecule, a conclusive analysis is complicated. Yet, if the water amount is even lower and > 80 wt% H_2_SO_4_, the potential‐determining species are likely cationic, i.e., the H_3_SO_4_^+^ and H_5_S_2_O_8_^+^ entities in concentrated and pure sulfuric acid, respectively.

Finally, we suggest a thermodynamically consistent definition of superacidity as being a medium in which the protochemical potential lies higher than that of pure sulfuric acid at about ${\mathrm{pH}}_{\mathrm{abs}}^{H_{2}O}$ = −24. Favorably, the definition is applicable to acidic systems in liquid, solid, and even gaseous phase.^[^ ^12^ ^]^

## Methods

Full details are disclosed with the Supporting Information, here we only provide some relevant additional details to the description of the Methods in the Results section above.

### SSE‐Method

The electrochemical silver/SSE^[^ ^34^ ^]^ operating with cell I was described before, although it mostly operated with the mercury/MSE.^[^ ^39^, ^40^ ^]^ Hitherto, measurements with sulfuric acid content higher than approx. 60 wt% H_2_SO_4_ were neither published with the SSE nor with the MSE electrodes. In initial experiments, a variation of cell I with an MSE instead of the SSE was utilized up to an H_2_SO_4_ concentration of about 80 wt% H_2_SO_4_. For handling and toxicity reasons of mercury and essentially the same response of both electrodes (see Figure 3a), we switched to the SSE as the standard for the core of the investigations. Here we assembled 20 implementations of cell I differing in S with the different H_2_SO_4_ concentrations as given in Table S4. Note that in each implementation of cell I, the H_2_SO_4_ concentration is constant throughout the entire cell, avoiding the emergence of significant diffusion potentials. Details on the measurements as well as on the measured data are given in Section S1. We define each H_2_O‐H_2_SO_4_ mixture as a discrete solvent S and combine the aqueous standard electrode potential of the hydrogen electrode with the relative activity of the proton in S. Then the potential of cell I, *E*_I_, can be expressed as Equation (M1):

(M1)

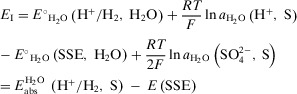




The standard potential value of the SSE in water is published as *E*°
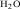
(SSE, H_2_O) = 0.654 V.^[^ ^55^ ^]^ Theoretically, the ${\mathrm{pH}}_{\mathrm{abs}}^{H_{2}O}$ of S1 = 1 wt% H_2_SO_4_ is close to 1 (approximating *a* 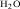
(H^+^, H_2_O) = *c* 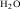
(H_2_SO_4_, H_2_O)/*c*° ≈ 0.1023 with the standard concentration *c*° = 1 mol L^−1^). With our measured *E*_I_(S1) value of −0.749 V and Equation (M1) we obtain *E*(SSE) = 0.690 V, which was assumed to be constant over the entire H_2_O‐H_2_SO_4_ mixing range, i.e., it was used to calculate $E_{\mathrm{abs}}^{H_{2}O}$(H^+^/H_2_) with Equation (M1) for all S. Then, the ${\mathrm{pH}}_{\mathrm{abs}}^{H_{2}O}$ value can simply be calculated with Equation (5).

### “Ideal” ILSB‐Method

This electrochemical method has been used to successfully determine the Gibbs energies of transfer of the silver ion,^[^ ^18^, ^21^, ^36^ ^]^ the chloride ion,^[^ ^36^ ^]^ and the proton^[^ ^56^ ^]^ between six, and in unpublished work, even 23 different solvents/solvent mixtures. The measured quantity in our case is the electric potential difference *E*_II_ of two electrochemical, hydrogen electrode driven half‐cells connected by an SB with a pure and “ideal” IL, in which similar diffusion constants of IL‐anion and IL‐cation eliminate diffusion potentials and minimize junction potentials in cell II. “Ideal” is put into quotation marks to indicate that any real IL does not behave completely ideal. See our original work^[^ ^18^, ^35^ ^]^ for requirements and evaluation of the “ideality.” Shibata et al. made measurements with a like cell with dilute H_2_SO_4_.^[^ ^57^ ^]^ In this work, C_8_MIm[FAP] is introduced as an "ideal" IL that we experimentally showed to be compatible with all acidity and reducity levels up to the stage of 100% sulfuric acid (Section S1.2). S_i_ and S_j_ refer to the content of H_2_SO_4_ in H_2_O in each of the half‐cells and thus, are representatives from the H_2_O‐H_2_SO_4_ system, i.e., a number of H_2_O‐H_2_SO_4_ mixtures or pure H_2_SO_4_. Since S_i_ and S_j_ are different in these half‐cells, an electric potential difference is caused by the difference of the proton's chemical potentials in the half‐cells. However, contrary to cell I, in this setup two liquid junction potentials (LJP), *E*_j1_ and *E*_j2_, occur, which cannot be known without the difference of the protochemical potentials. Nevertheless, the sum *E*_j1_ + *E*_j2_ = *x*_II_ can be assessed, as shown before with Ag^+^‐, Cl^−^‐, and H^+^‐ cells using the “ideal” IL N_2225_[NTf_2_], which unfortunately is not compatible with pure H_2_SO_4_.^[^ ^18^, ^21^, ^36^ ^]^ We here give a short description of this assessment for C_8_MIm[FAP]. The reaction of cell II can be written as Equation (M2).

(M2) $$H^{+}\left(\mathrm{solv},\;S_{i}\right)\to H^{+}\left(\mathrm{solv},\;S_{j}\right)$$

By measuring the potential difference in cell II with S_i_ = H_2_O and S_j_ = H_2_SO_4_ the Gibbs energy of transfer of the proton Δ_tr_ *G*°(H^+^, H_2_O→ H_2_SO_4_) can be obtained in principle. However, the dataset is not sufficient to assess *x*. To examine whether consistent data are obtained with this method, i.e., that *E*_j1_ + *E*_j2_ is constant, cell II is assembled and measured in different implementations. Cell II can be considered as a connection (i.e., the measured potential difference value *E*_I_) between two nodes (i.e., the half‐cell potentials). The mathematical equivalent is a linear equation, the two variables of which are the potential values *E*_left_ and *E*_right_ of the half‐cells (referenced against a freely defined zero point, purposefully against a half‐cell with known ${\mathrm{pH}}_{H_{2}O}$ value), and *E*_II_ is the coefficient. Three half‐cells, 1, 2, and 3, can be measured in implementations of 1 versus 2, 1 versus 3, and 2 versus 3, thereby creating a system of linear equations with three variables and three coefficients, or a “triangle” with three connections between three half‐cells. Even more half‐cells result in an overdetermined system of equations, i.e., the number of variables *n* is smaller than of coefficients *m*. A network between the nodes emerges, whose meshes are tighter the more half‐cells are measured against each other. The higher the ratio *m*/*n* the more reliable the solution of the equation system, i.e., the tighter and more robust the network is; cf. to Figure 3b, where the arrowhead indicates the left half‐cell. The solution of such an equation system can be found by the least squares method, which typically does not satisfy any of the equations exactly but gives an optimized value for each variable with an individual residue for each equation and an overall uncertainty expressed as *σ*.^[^ ^58^ ^]^ Similar to, i.e., the consistency standard deviation *s*, successfully used to build consistent p*K*_a_ scales.^[^ ^59^ ^]^ A small *σ* value indicates a high constancy of the above‐mentioned sum *E*_j1_ + *E*_j2_ and thus a high consistency of the obtained data, i.e., a high precision of measurements. It is important to stress, however, that the *σ* value only takes into account random effects, affecting different potential measurements differently. If there is a systematic effect, e.g., one that decreases all the potential differences, then this will lead to *σ* showing underestimated measurement uncertainty. In other words: With the least squares method, the constancy of *x* can be evaluated, not its magnitude. From the assembled 30 implementations of cell II, a network of nodes was created as described above and shown in Figure 3b. Note that, contrary to the SSE‐method, in each implementation the H_2_SO_4_ concentration is different in the half‐cells. From the evaluation and due to the experimentally very challenging measurements, especially with the highly viscous and reactive concentrated H_2_SO_4_ solutions, for our *m*/n = 42/12 system, we obtained *σ* as 60 mV or 1 pH unit, respectively, and, conservatively, provisionally assume *x* to be smaller than 2–3 pH units or 0.12–0.18 V (Section S1.3). Under these restrictions, *E*_II_ measures without any other contributions the Gibbs energy of reaction given in Equation (M2), i.e., Δ_tr_ *G*°(H^+^, S_i_→ S_j_). As above, we define each H_2_O‐H_2_SO_4_ mixture as a discrete solvent S, and ${\mathrm{pH}}_{\mathrm{abs}}^{H_{2}O}$ can be calculated with Equation (5). Details on the measurements as well as on the measured data and their analysis are given in Section S1.

### QMB‐Method

The dissociation of the protonated indicator base B used for *H*_0_ determination was used to determine the chemical potential of the proton with the BFHC in Figure 1. Here, the gas phase basicities of the bases were obtained from high‐accuracy quantum chemical computations. The gas‐phase structures were optimized with ORCA 5.0.3^[^ ^60^, ^61^, ^62^, ^63^ ^]^ at the double‐hybrid DSD‐BLYP^[^ ^64^ ^]^/def2‐TZVPP^[^ ^65^ ^]^ level of DFT, followed by subsequent DLPNO‐CCSD(T)^[^ ^24^, ^25^ ^]^ single points extrapolated to the CBS‐limit, that reached chemical accuracy where reliable reference data was available (Section S2). The Gibbs solvation energies of the indicator bases, Δ_solv_ *G*°(B, H_2_SO_4_), and their protonated form, Δ_solv_ *G*°(BH^+^, H_2_SO_4_), were computed with COSMO RS^[^ ^66^ ^]^ and include both the constant of autoprotolysis $pK_{\mathrm{ap},H_{2}{\mathrm{SO}}_{4}}$ = 3.10 and of self‐dehydration $pK_{\mathrm{ip},H_{2}{\mathrm{SO}}_{4}}$ = 3.85 of sulfuric acid.^[^ ^32^ ^]^ The acidity constants (p*K*_a_ values) of the protonated bases referring to the sulfuric acid reference state, $pK_{a,H_{2}{\mathrm{SO}}_{4}}$(BH^+^, H_2_SO_4_), were obtained from ${\mathrm{pH}}_{H_{2}{\mathrm{SO}}_{4}}$, $pK_{a,H_{2}O}$(BH^+^, H_2_SO_4_), and *H*_0_ of pure sulfuric acid:

(M3) $$\begin{array}{cc} & pK_{a,H_{2}SO_{4}}\;\left(BH^{+},\;H_{2}SO_{4}\right)=pH_{H_{2}SO_{4}}\left(H_{2}SO_{4}\right)\\ & +pK_{a,H_{2}O}\left(BH^{+},\;H_{2}SO_{4}\right)-H_{0}\left(H_{2}SO_{4}\right) \end{array}$$

Assuming an exceedingly small indicator base concentration, only ions resulting from the autoprotolysis and self‐dehydration of sulfuric acid are present for COSMO RS solvation. The concentrations *c*(H_3_SO_4_^+^, H_2_SO_4_) = *c*(HSO_4_^−^, H_2_SO_4_) = 0.028 mol L^−1^ result from $pK_{\mathrm{ap},H_{2}{\mathrm{SO}}_{4}}$ and *c*(H_3_O^+^, H_2_SO_4_) = *c*(HS_2_O_7_^−^, H_2_SO_4_) = 0.012 mol L^−1^ result from $pK_{\mathrm{ip},H_{2}{\mathrm{SO}}_{4}}$. Taking further _s_ *f* = 1 for all ionic species, since the concentrations are rather low, ${\mathrm{pH}}_{H_{2}{\mathrm{SO}}_{4}}$(H_2_SO_4_) = −lg (0.028 + 0.012) = 1.40. The $pK_{a,H_{2}O}$(BH^+^, H_2_SO_4_) values of BH^+^ given in Table 1  were published by Gillespie et al.^[^ ^33^ ^]^ and *H*_0_ = −11.93 is the value of the acidity function that was determined with 100% H_2_SO_4_ by the same group.^[^ ^33^ ^]^ Inserting the above values into Equation (M3) leads to $pK_{a,H_{2}{\mathrm{SO}}_{4}}$(BH^+^, H_2_SO_4_) values for different bases listed in Table 1. Figure 1 includes nitrobenzene as a worked example for any indicator base B.

### QME‐Method

In this method, we used the COSMO RS program to calculate the Gibbs solvation energies by modeling the realistic composition of (aqueous) sulfuric acid from its constituent ions and neutrals. Pure sulfuric acid was modeled as a composition with constituents resulting from its known dissociation constants, as described for the QMB method.^[^ ^32^ ^]^ Yet, the exact composition of aqueous sulfuric acid over the entire concentration range is less investigated and was modeled based on reasonable assumptions supported by additional experimental results. The HSO_4_^−^ and SO_4_^2−^ concentrations were taken from Raman data by Young et. al.^[^ ^41^ ^]^ and adapted by polynomial regression curves. For details see Section S2.3. The HSO_4_^−^ equilibrium concentration was adjusted to fulfill the condition [HSO_4_^−^] + [SO_4_^2−^] = *c*(H_2_SO_4_, S) until the maximum of the HSO_4_^−^ concentration was determined by a regression fit to the Raman data. Thereafter, the dissociation degree was kept as close as possible to the one obtained from Raman measurements. The liberated proton was described as a set of different protonated water clusters H(H_2_O)_n_^+^ with *n* = 1–5 that were balanced to keep the mixture's overall charge neutral and stoichiometric. Additional chemical entities were included at infinite dilution. Three different general mixture compositions were evaluated: starting with the Raman data, they were adjusted to diminish the concentrations of HSO_4_^−^ and SO_4_^2−^. For each of those general compositions, mixtures between 5 and 100 wt% H_2_SO_4_ in water were generated. Finally, the Gibbs free energy of solvation of the proton Δ_solv_ *G*(H^+^, H_2_SO_4_, 5%–100%) was obtained from the calculated solvation energies of the different chemical entities E in equilibrium with its protonated forms EH^+^ present in the mixture through the QME‐BFHC shown in Figure 4a. The gas‐phase basicities of the relevant entities E were optimized with ORCA 5.0.1^[^ ^60^, ^61^, ^62^, ^63^ ^]^ at DSD‐PBEP86^[^ ^67^ ^]^/def2‐TZVPP^[^ ^65^ ^]^ level of theory, with subsequent single‐point calculations approaching the DLPNO‐CCSD(T)/CBS level.^[^ ^24^, ^25^ ^]^ A detailed description of the theoretical procedure is given in the Supporting Information.

## Author Contributions

M.B. performed all electrochemical measurements with SSE and ILSB setup, R.S. performed all quantum chemical calculations, T.K. did perform the experimental checks of the stability of H_2_SO_4_ in the presence of Pt‐black and hydrogen, A.H., J.N., and E.L. performed the MSE measurements, V.R., D.H., I.L., and I.K. devised the project, and V.R., I.L., and I.K. supervised the project, V.R., R.S., and I.K. wrote the manuscript.

## Conflict of Interests

The authors declare no conflict of interest.

## Supporting information



Supporting Information

## Data Availability

The Supporting Information contains details on experimental (1.1 – 1.4) and computational details (2.1 – 2.3) and results (1.5 – 1.6) and (3), respectively.
