# Supplementary material for: About a Trillion Times More Acidic than Expected? On the Difference Between the Hammett H 0 and the Unified pH Acidity of Sulfuric Acid
Source: Angew Chem Int Ed Engl. 2025 Nov 17;64(52):e06429. doi: 10.1002/anie.202506429 (PMC12723472; doi:10.1002/anie.202506429)
Supplement: Supplementary file 1 — Supporting Information [file ANIE-64-e06429-s001.pdf]

Supplementary Information to

About a Trillion Times more Acidic than Expected? On the Determination of the Unified Acidity of Sulfuric Acid

Valentin Radtke, Monika Bäuerle, Regina Stroh, Timo Kienzle, Daniel Himmel, Agnes Heering, Jaak Nerut, Enn Lust, Ivo Leito, and Ingo Krossing

Contents

1 Experimental Details 2
1.1 General Aspects 2
1.2 Synthesis and Characterisation of [C_8MIm][FAP] 4
1.3 Assessment of the Ionic Liquid Salt Bridge (ILSB) 8
1.4 Assessment of the H_2 Electrode 9
1.5 SSE Method 11
1.6 ILSB Method 13
2 Computational Details 18
2.1 General Aspects 18
2.2 Pure Sulfuric Acid 18
2.2.1 Indicator Base Approach to determine Gibbs Energies of Proton Solvation 21
2.3 H_2O-H_2SO_4 Mixtures 25
2.3.1 First Approximation of the Composition of Aqueous Sulfuric Acid 27
2.3.2 Adjusting the Concentrations of the First Approximation 31
3 Computation Results 37
3.1 Polynomial Regression to the Raman Data 37
3.2 Concentrations 38
3.3 Gas-phase Enthalpy and Entropy of the Proton 44
3.4 Point Groups and Symmetry Number 44
3.5 Calculated Gas-Phase Energies 45
3.6 Calculated Solvation Energies 49
3.6.1 Solvation energies obtained from the concentrations of the first mixture 50
3.6.2 Solvation energies obtained from the concentrations of the second mixture 55
3.6.3 Solvation energies obtained from the concentrations of the third mixture 60
4 Literature 65

1 Experimental Details

1.1 General Aspects

Experiments were carried out under inert argon atmosphere using standard Schlenk techniques and glove boxes with an argon atmosphere containing less than 1 ppm H_2O and O_2. Glassware was cleaned by immersion in a KOH/iPrOH bath overnight, rinsing with water and immersion in a HCl bath for at least 30 min. After rinsing with deionized water, glassware was dried in an oven at 180 °C overnight. The synthesis of [C_8MIm][FAP] and the H_2SO_4 dilution series were not carried out under inert conditions.

Chemicals were used as purchased, if not stated otherwise. They were stored in a Glovebox with argon or nitrogen atmosphere except for Ag_2SO_4. Ag_2SO_4 was purified by recrystallisation from concentrated H_2SO_4. A list of all used chemicals is shown in Table S1.

Synthesis of [C_8MIm][FAP]. See Section 1.2

Preparation of H_2O-H_2SO_4 mixtures. H_2SO_4 (95%) was used as purchased. Aqueous solutions of H_2SO_4 with concentrations of 50% to 90% were prepared by dilution of the 95% H_2SO_4 with deioinized water. The 50% H_2SO_4 solution was then further diluted to obtain the respective concentrations. Density data from literature served as basis for the dilution series.[1]

Preparation of 98 wt.-% and 100 wt.-% H_2SO_4. 95% H_2SO_4 was distilled twice in vacuo to obtain 98% H_2SO_4. Pure 100% H_2SO_4 was prepared via conductometric titration. Therefore, fuming H_2SO_4 (20–30% SO_3) was added dropwise into the freshly distilled H_2SO_4 until a minimum conductivity of 10.4 mS·cm^{-1} was reached.[2] Both were stored under inert atmosphere.

Table S1 List of the used chemicals, manufactures and purities.

Chemical	Manufacturer or Origin/ Purity
Ag wire	MaTecK, ø 1 mm / 99.9999%
Ag_2SO_4	Merck / 98.5%
$CDCl_3$	Euriso-top
CD_2Cl_2	Deutero
[C_8MIm][FAP]	Synthesized as described in Section 1.2
[C_8MIm]Br	IoLiTec
H_2	Sauerstoffwerk Friedrichshafen, 5.0
HNO_3 65%	Fischer Chemical
H_2SO_4 95%	Fluka Analytical
H_2SO_4 fuming (20-30% free SO_3)	Acros Organics / >99%
K[FAP]	Merck KGaA

Saturated Silver/Silver Sulfate Electrodes (SSE) were manufactured in-house. For the deposition of Ag_2SO_4 on the polished silver wire, the latter was immersed in a saturated solution of Ag_2SO_4 in H_2SO_4 (2 mol L^{-1}, degassed for 15 min). A current of 20 mA was applied for 1.5 h with the silver wire as working electrode and a platinum wire as counter/reference electrode resulting in a white coating of the silver wires. After that the Ag/Ag_2SO_4 electrodes were rinsed with deionized water and dried at 50 °C for 1 h. The

electrodes were stored in a saturated solution of Ag_2SO_4 in H_2SO_4 (2 mol L^{-1}), before use they were rinsed with deionized water and dried.
SSEs were freshly prepared before each measurement by immersing the coated Ag wires in a saturated solution of Ag_2SO_4 in H_2SO_4 solutions of the respective concentration. The reference compartment was separated from the hydrogen electrode compartment by glass frits, glass wool, and a Luggin capillary, as ca be seen in the scheme of Figure 2a in the main text.

Saturated Mercury/Mercurous Sulfate Electrodes (MSE) were Radiometer Analytical REF621 Reference Electrodes. Their porous pin junctions were cut off and the inner saturated K_2SO_4 solution was drained. This made possible to change the inner solution of the MSE.
The Electrode was rinsed with water and then with 0.5 mol L^{-1} sulfuric acid. The Electrode was left to stay in 0.5 mol L^{-1} sulfuric acid at 45 °C for at least one week. Solution was renewed daily. This step was crucial to remove K_2SO_4 from the inside of electrode; otherwise, K_2SO_4 shifted the potential of MSE. Then the electrode was taken to a higher concentration, again at 45 °C for a week and solution was renewed. There was one MSE per sulfuric acid concentration. Starting from 14.6 mol L^{-1} the glass wool in the MSE reacted with sulfuric acid and turned dark brown to black. Then the glass wool was changed to new silanized glass wool that was boiled once in concentrated sulfuric acid and twice in MilliQ and then dried. All measurements with sulfuric acid mixtures were done successively and then the MSE solution was changed to the next concentration.

Hydrogen Electrodes were manufactured in-house. Pt flag (0.5 cm^2) electrodes in a glass tube were purchased from PHYWE Systeme & Co. KG. The electrode surface was purified by immersion in aqua regia for 15 min at 50 °C and washed with deionized water. The cleaned electrode was immersed in an aqueous solution of $H_2[PtCl_6]$ (0.04 m) and $Pb(CH_3COO)_2$ (1 mm). Applying a current of 30 $mA{\cdot}cm^{-2}$ for 10 min resulted in platinum black coating. The platinized electrode was washed with deionized water and acetone. For the use as hydrogen electrode, hydrogen gas was fed into the solution via a glass tube flowing over the platinum surface. The hydrogen pressure within the half-cells was maintained at 10^5 Pa.

Open Circuit Voltage (OCV) measurements were performed with a Biologic SP-300 or VMP3 potentiostat controlled with the software EC-Lab (V11.21). Measurements were performed at room temperature, and if not other stated, for constant potential curves the cell potential was read out after 3 h.

Conductivity measurements were performed with a METROHM 712 conductometer (C = 0.779 cm^{-1}) at room temperature with an assumed error of 1.3% for values below 2 $\mu S\,cm^{-1}$ and otherwise 0.5%. Conductivities were only determined after no significant change in conductivity and temperature was observed.

Raman spectra were recorded with Bruker (VERTEX 70) equipped with a Nd-YAG-Laser.

NMR spectra were recorded on a Bruker AVANCE III HD 300 MHz or a Bruker AVANCE II^+ 400 MHz WB spectrometer. ^1H-NMR chemical shifts are reported in ppm (δ) relative to tetramethylsilane (TMS) and referenced using the chemical shifts of residual proton solvent resonances (CD_2Cl_2: δ = 5.32 ppm, $CDCl_3$: δ = 7.26 ppm, toluene-d_8: δ = 2.08 ppm ($CH\mathit{D}_2$))[3] (H_2SO_4: δ = 10.21 ppm (this work)). Data analysis was performed using Bruker TOPSPIN 3.5 software. The change of the ^1H-NMR spectrum reference frequency (SR) is converted to heteronuclear X-NMR (X = ^{19}F, ^{31}P) SR with the spectrometer frequency SF according to Equation S1. (SR(X) = SR(^{1}H)·SF(X)/SF(^{1}H)).

$$SR(X) = SR(\,^1H)\,\frac{SF(X)}{SF(\,^1H)} \qquad \text{S1}$$

PGSTE-NMR: the DOSY NMR spectrum for [C_8MIm][FAP] was recorded on a Bruker AVANCE DSX 500 spectrometer equipped with a Diff30 gradient unit and BAFPA-40 amplifier, allowing a maximal gradient of 1180 G·cm^{-1}. Measurements were performed at 298 K in sealed 3 mm NMR tubes using the "diffSte" pulse program.
Data analysis was performed using Bruker TOPSPIN 3.2 software. Cation diffusion constants D_+ were extracted from ^{1}H spectra, anion diffusion constants D_- were extracted from ^{19}F spectra. Fitting was done with the Stejskal-Tanner equation (Eq. S2) by using the integrals of the NMR signals.

$$\frac{I(\delta,\Delta,g)}{I(\delta,\Delta,g=0)} = \exp\left[-\gamma^2\delta^2 g^2 D\,(\Delta-\frac{\delta}{3})\right] \qquad \text{S2}$$

1.2 Synthesis and Characterisation of [C_8MIm][FAP]

[C_8MIm]Br (20.8 g, 75.4 mmol) and K[FAP] (36.4 g, 75.2 mmol) in CH_2Cl_2 (190 mL) and water (190 mL) were stirred for 48 h at room temperature. The organic phase was separated and the aqueous phase was extracted with CH_2Cl_2 (3 × 10 mL). The combined organic phases were washed with water (7 × 1 mL) until no precipitation was observed in the washing water after the addition of aqueous $AgNO_3$ solution (1 mol L^{-1}). CH_2Cl_2 was removed in vacuo and [C_8MIm][FAP] was isolated as yellow oil (46.4 g, 72.5 mmol, 96%) after drying at 100 °C for 6 h.
The NMR spectra was measured in a 3 mm NMR tube with toluene-d_8 as external lock and calibrated to δ = 2.08 ppm (CHD_2). Figure S1 shows the ^{1}H-NMR spectrum of the IL, the signals are assigned to the [C_8MIm]$^+$ cation. The aromatic ring protons (a) and (b) can be found at 7.94 ppm and 7.02 ppm, coupling of the protons b result in a multiplet. The singlet of the methyl group (d) attached to the N-atom occurs at 3.65 ppm. For the octyl chain four signals can be observed at 3.91 (c), 1.71 (e), 1.16 (f) and 0.71 ppm (g), the ^3J(H, H) coupling results in triplets with a coupling constant of 7.3 Hz for (c) and 6.9 Hz for (g).
^{1}H-NMR (400.17 MHz, external lock toluene-d_8, 298 K): δ = 0.71 (t, ^3J(H, H) = 6.9 Hz, 3 H, $NCH_2CH_2CH_2CH_2CH_2CH_2CH_2CH_3$), 1.16 (m, 10 H, $NCH_2CH_2CH_2CH_2CH_2CH_2CH_2CH_3$), 1.71 (m, 2 H, $NCH_2CH_2CH_2CH_2CH_2CH_2CH_2CH_3$), 3.65 (s, 3 H, NCH_3), 3.91 (t, ^3J(H, H) = 7.3 Hz, 3 H, $NCH_2CH_2CH_2CH_2CH_2CH_2CH_2CH_3$), 7.02 (m, 2 H, NCHCHN), 7.94 (s, 1 H, NCHN) ppm.
^{19}F-NMR (376.54 MHz, external lock toluene-d_8, 298 K): δ = −116.77 (dm, ^2J(P, F) = 98 Hz, 4 F, CF_2CF_3), −116.21 (dm, ^2J(P, F) = 83 Hz, 2 F, CF_2CF_3), −88.35 (dm, ^1J(P, F) = 901 Hz, 4 F, PF_2), −82.87 (m, 6 F, CF_2CF_3), −81.09 (m, 3 F, CF_2CF_3), −44.56 (dm, ^1J(P, F) = 891 Hz, 1 F, PF) ppm.
^{31}P-NMR (161.99 MHz, external lock toluene-d_8, 298 K): δ = −147.29 (dtm, ^1J(P, F) = 891 Hz, ^1J(P, F) = 901 Hz, ^2J(P, F) = 98 Hz) ppm.

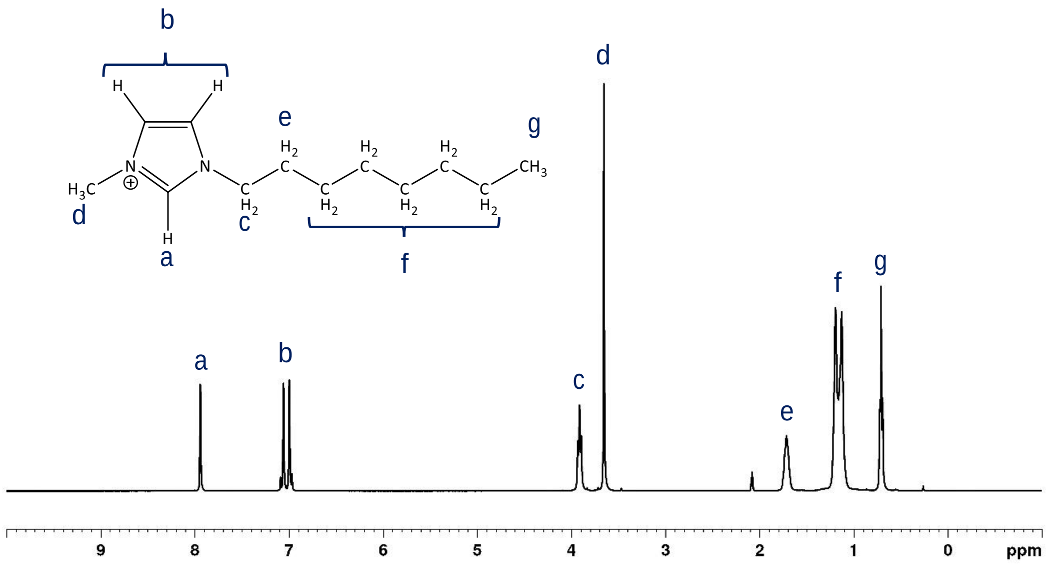

Figure S1: ^{1}H-NMR (400.17 MHz, 298K) spectrum of the neat IL [C_8MIm][FAP].

The [FAP]$^-$ anion is analysed by ^{19}F- and ^{31}P-NMR (Figure S3 and Figure S4), due to the octahedral molecular geometry of the [FAP]$^-$ anion, two isomers – facial and meridional – are possible as depicted in Figure S2

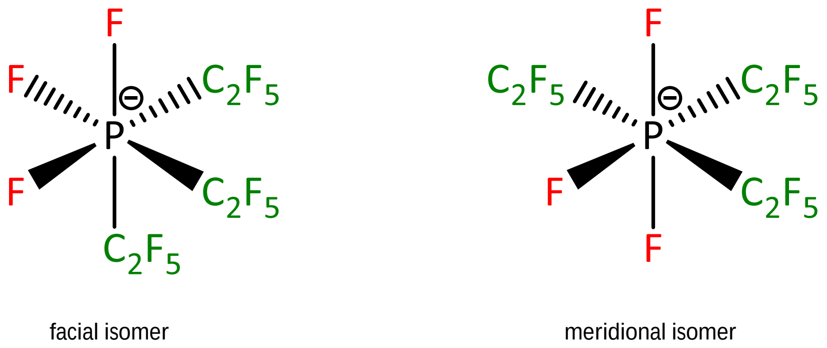

Figure S2: Schematic structures of the [FAP]$^-$ anion with the facial (fac) isomer on the left and the meridional (mer) isomer on the right side.

below.

According to literature the synthesis of [C_2MIm][FAP] yields the meridional isomer, which equilibrates thermally to 15% of the facial isomer.[4] In comparison to this, Mezger *et al.* could not detect any of the facial isomer for the IL [C_{18}MIm][FAP].[5] The conducted synthesis of [C_8MIm][FAP] also yielded in the meridional isomer, no signals of the facial isomer are observable in the ^{19}F-NMR spectrum.

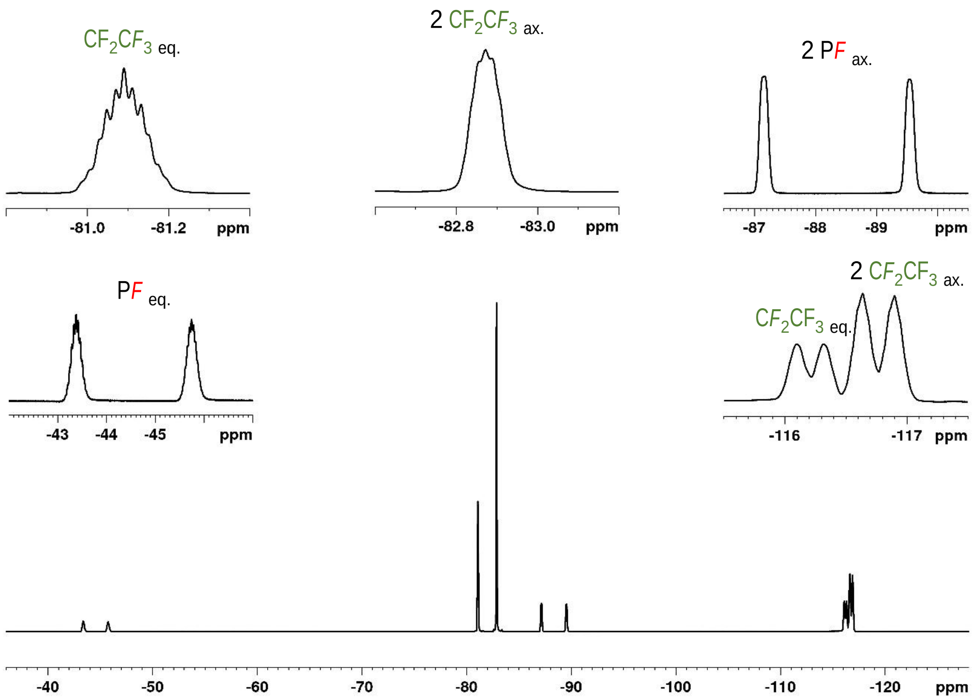

Figure S3: ^{19}F-NMR (376.54 MHz, 298K) spectrum of the neat IL [C$_8$MIm][FAP].

The signals for the two axial F-atoms attached to the phosphorous occur at −88.35 ppm as doublet of multiplets due to the ^{1}J(P, F) coupling with 901 Hz. Much more low field the signal of the equatorial F-atom is observable at −44.56 ppm with a coupling constant ^{1}J(P, F) = 891 Hz. For the perfluorinated ethyl groups four signals appear. The C*F*$_3$ groups can be found at −82.87 ppm (ax.) and −81.09 ppm (eq.) splitting into multiplets. Two pairs of doublets occur for the C*F*$_2$ groups, at −116.77 ppm with ^{2}J(P, F) = 98 Hz the axial groups and at −116.21 ppm the equatorial group with ^{2}J(P, F) = 83 Hz. The doublets are further split into multiplets due to ^{2}J-coupling with the CF_3 group.

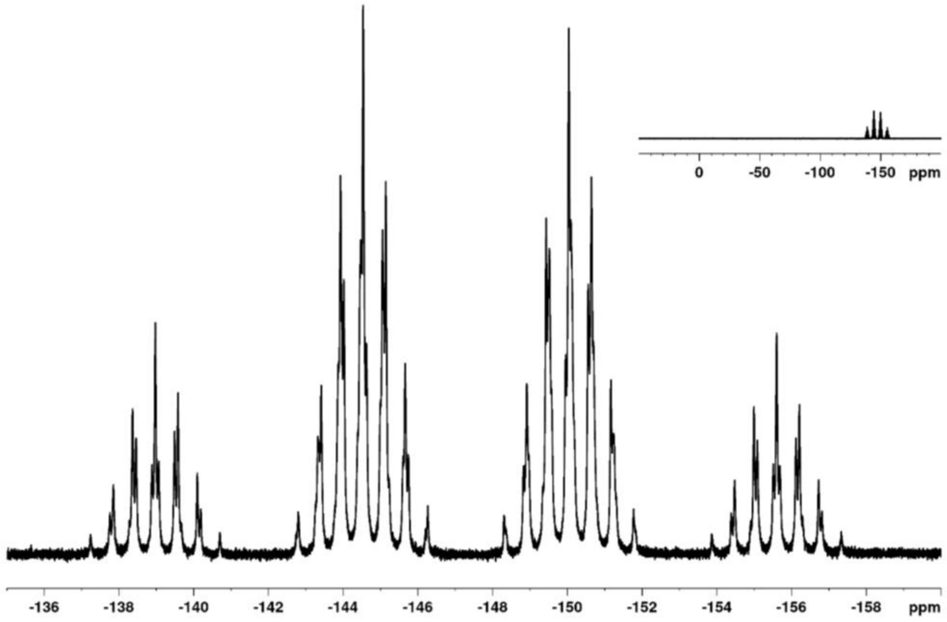

Figure S4: ^{31}P-NMR (161.99 MHz, 298K) spectrum of the neat IL [C$_8$MIm][FAP].

In the ^{31}P-NMR spectrum one signal occurs at −147.29 ppm, proving that only one phosphorous species is present, it can be considered as doublet of triplets of multiplets. ^{1}J-coupling with the attached F-atoms leads to the split-up into a doublet with ^{1}J(P, F) = 891 Hz for the equatorial and F-atom and splitting into triplets with ^{1}J(P, F) = 901 Hz for the two axial F-atoms. Due to ^{2}J- and ^{3}J-coupling with the perfluorinated ethyl groups, a further split-up into multiplets is observed.

For the application as ILSB it is of uttermost importance to be certain that only one of the $[FAP]^-$ isomers is present, as the ion interaction between cations and anions differs, and hence different diffusion constants might occur for the isomers. Only the meridional anion was found in the product, which complies with literature.[5] The meridional isomer is energetically most favourable conformation according to DFT-experiments by Voroshylova, although the cation is only coordinated by two F-atoms instead of three F-atoms as in the facial isomer.[6] For small cations a stabilising effect of the facial anions in the ion pair is observed, attributed to the energetically advantageous cup-like geometry, leading to a partial thermal transition from meridional to facial for $[C_2MIm][FAP]$.[4,6] The preferable formation of the meridional isomer can be especially detected for larger cations, such as the $[C_8MIm]^+$ in this work and the $[C_{18}MIm]^+$ as mentioned above.

Stability of $[C_8MIm][FAP]$ against $[H_2SO_4]$

A 1:1 mixture of $[C_8MIm][FAP]$ and $[H_2SO_4]$ was sealed in a NMR tube and NMR spectra were measured after one day, two days and one week (Figure S5 and Figure S6). The ^{31}P-NMR spectrum reveals the formation of at least two further phosphorous species. After two days an additional signal can be observed at −46.32 ppm and can be considered as triplet of septets with a ^{1}J(P, F) coupling constant of 1002 Hz and a ^{2}J(P, F) coupling constant of 122 Hz. This signal is attributed to the cleavage of one P–F bond, resulting in the neutral molecule $PF_2(CF_2CF_3)_3$. The corresponding F-signals are to be found at −51.16 ppm as a doublet of multiplets ($P\mathit{F}_2$) and for the perfluorinated ethyl groups at −83.64 ppm (m, $CF_2C\mathit{F}_3$) and −115.24 ppm (dm, $C\mathit{F}_2CF_3$). The formed HF, respectively SiF_4, is to be found at −164.40 ppm. Furthermore, the ^{19}F-NMR spectrum shows the partial conformational transfer into the facial isomer (−68.38 ppm, dm, ^{1}J(P, F) = 793 Hz, $P\mathit{F}_3$). The additional P-signal at −20.84 ppm after one week can be assigned to the phosphine oxide $O{=}P(CF_2CF_3)_3$.

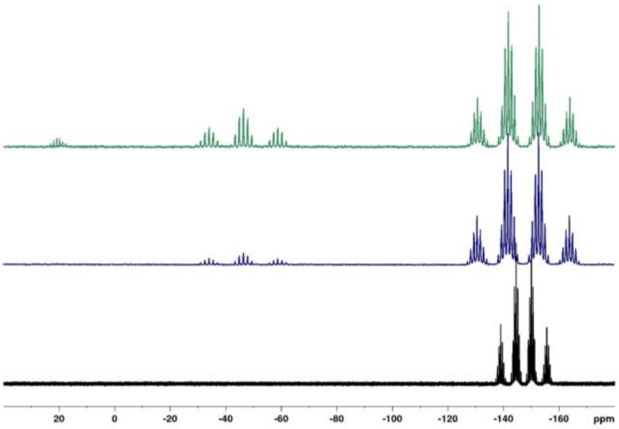

Figure S5: ^{31}P-NMR (161.99 MHz, resp. 81.01 MHz) spectra of a 1:1 mixture of $[C_8MIm][FAP]$ and H_2SO_4 after one day (black), two days (blue) and one week (green) at 298 K.

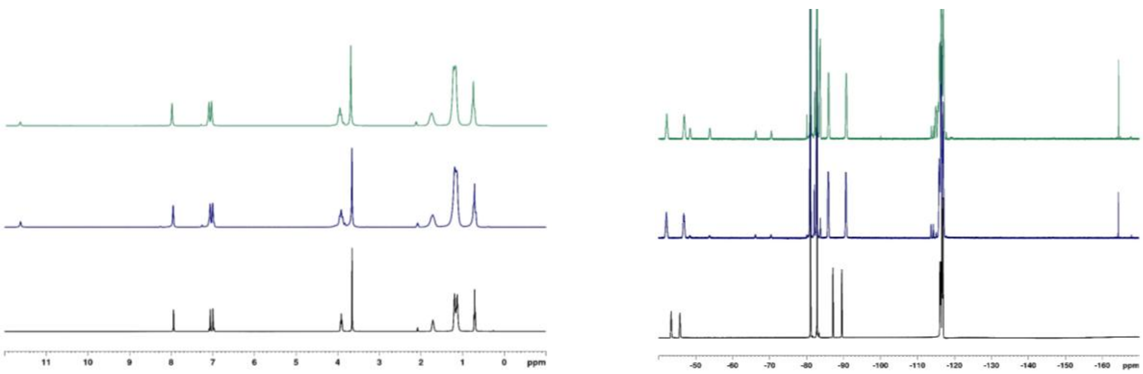

Figure S6: ^{1}H-NMR (400.17 MHz, resp. 200.13 MHz) and ^{19}F-NMR (376.54 MHz, resp. 188.31 MHz) spectra of a 1:1 mixture of $[C_8MIm][FAP]$ and H_2SO_4 after one day (black), two days (blue) and one week (green) at 298 K.

Diffusion Constants, Conductivity and Ionicity. The ion specific self-diffusion constants of [C_8MIm][FAP], [C_2MIm][FAP], [C_8MIm][OTf] and [C_4MIm][OTf] were measured with PGSE-NMR experiments at room temperature. Cation diffusion constants D_+ were extracted from ^{1}H-spectra and anion diffusion constants D_- were extracted from ^{19}F-spectra. Each IL was measured at least twice. Using the integrals of the NMR signals fitting was done with the Stejskal-Tanner equation (Eq. S2). The arithmetic mean of these measurements are given in Table S2, together with conductivity data and ionicities. In general, the self-diffusion constants decrease with increasing cation size. For example, in [C_2MIm][FAP] the cation is nearly twice as fast as the anion, while in [C_8MIm][FAP], both ions are diffusing almost equally fast. The ion self-diffusion constants in [C_8MIm][FAP] are approximately twice the size of the diffusion constants in [C_8MIm][OTf]. Contradicting common expectations, the cations diffuse faster when they are combined with the larger anion [FAP]$^-$.

Table S2: Experimental self-diffusion constants, conductivity and ionicity values.

IL	D_+ / 10^{-11} m^2·s^{-1}	D_- / 10^{-11} m^2·s^{-1}	$\sigma_{calc.}$ / mS·cm^{-1}	$\sigma_{exp.}$ / mS·cm^{-1}	I
[C_8MIm][FAP]	0.814±0.006 (51%)	0.7854±0.0014 (49%)	1.388±0.005	0.91±0.02	0.66

The most relevant information with regard to a suitable ILSB deriving from the PGSE-experiments, is that the self-diffusion constants of the ions [C_8MIm]$^+$ and [FAP]$^-$ are very similar. 51% of ion diffusion is attributed to the cations and 49% to the anions. Therefore, when using this IL in electrochemical measurements the majority of the LJPs at both ends of the ILSB should cancel as for [N_{2225}][NTf_2].[7,8]
Ionicity. In order to determine ionicities conductivity measurements were performed, leading to values presented in Table S2. The IL [C_8MIm][FAP] has an ionicity of 0.66 meaning that 34% of the ions are not available for charge transport, this has interfering influences for electrochemical applications.

1.3 Assessment of the Ionic Liquid Salt Bridge (ILSB)

The ILSB filled with [C_8MIm][FAP] was used to measure the Gibbs transfer energy of the Ag^+ ion between some selected organic solvents S_i and S_j (acetonitrile MeCN, propylene carbonate PC, and ethanol EtOH) as it was done by us with the ILSB filled with [N_{2225}][NTf_2] (amyltriethylammonium bis(trifluoromethanesulfonyl)imide.[7,8] The ILSB of cells III was filled with [C_8MIm][FAP], that of cells IV with [N_{2225}][NTf_2].

Ag | Ag^+ (S_j) | [C_8MIm][FAP] | Ag^+ (S_i) | Ag   cell III
Ag | Ag^+ (S_j) | [N_{2225}][NTf_2] | Ag^+ (S_i) | Ag   cell IV

We consider the probability as high that within cells IV the LJPs cancel almost completely, that means $x_{IV} = 0$,[7,8] and thus, the $\Delta_{tr}G°(Ag^+, S_i{\rightarrow}S_j)$ can be determined directly. The comparison with these data gives an indication of the LJP contributions x_{III} that exist in cell III: $E_{III} - E_{IV} = x_{III}$. The result of this comparison is given in Table S3, where the mean of the measured values are given, and the values are not optimized by the network analysis (however, the optimized values give essentially the same result). It can be seen that the uncertainty caused by x_{III} corresponds to approximately 0.24 pH units.
However, we point out that this assessment applies strictly only to the systems under consideration and can only be transferred to cell II that includes x_{II} (i.e. to H_2O-H_2SO_4 mixtures) with the necessary caution.

Table S3: Potential differences E_{III} and E_{IV} and the resulting LJP contributions in cell III in mV.

$S_i \rightarrow S_j$	E_{III} / mV	E_{IV} / mV	x_{III} / mV
MeCN → PC	−443.1	−415.8	−27.3
MeCN → EtOH	−291.0	−277.5	−13.5
EtOH → PC	−145.0	−143.5	−1.5
mean			−14.1 (0.24 pH)

1.4 Assessment of the H_2 Electrode

To assess possible side reactions concomitant with a mixed electrode potential at the Pt|H_2|H^+(H_2SO_4) electrode, we analysed samples of concentrated sulfuric acid as well as its suspensions with platinum black with and without hydrogen atmosphere (stored in NMR tubes safely sealed with a J. Young valve) by NMR and Raman spectroscopy. After a measurement series of 8 days, Raman spectra (Figure S7) and the NMR (Figure S8) did not change with reference to the neat concentrated sulfuric acid and we did not observe any evidence for any reduction products. In the ^{1}H-NMR spectra focussed on the H_2-Signal at 4.06 ppm (Figure S8 bottom)), the gradual dissolution of H_2 is observed in the layered samples. In the sample pre-treated by bubbling H_2, the hydrogen concentration remains stable. Furthermore, in samples containing platinum, the spectra indicate that H_2 either diffuses through the concentrated sulfuric acid into the platinum (layered sample) or has already diffused into the platinum before the initial measurement (bubbled sample).

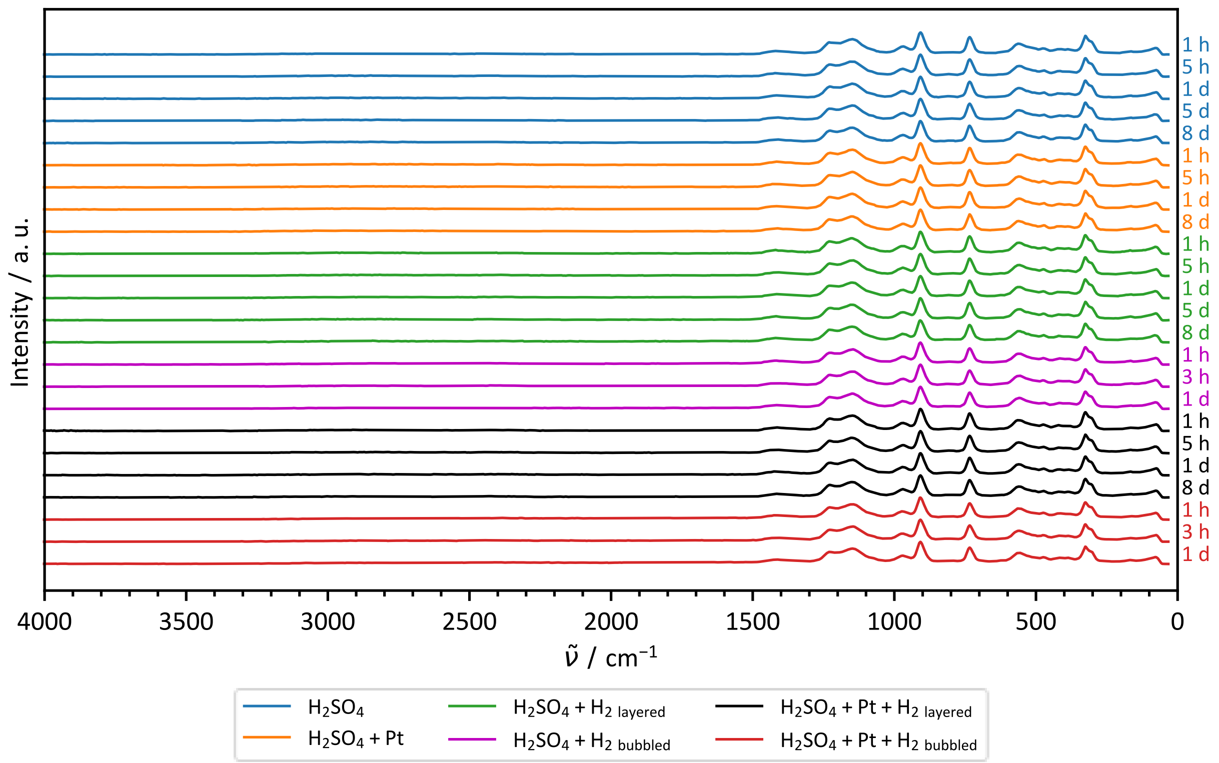

Figure S7: Raman data of the samples as indicated.

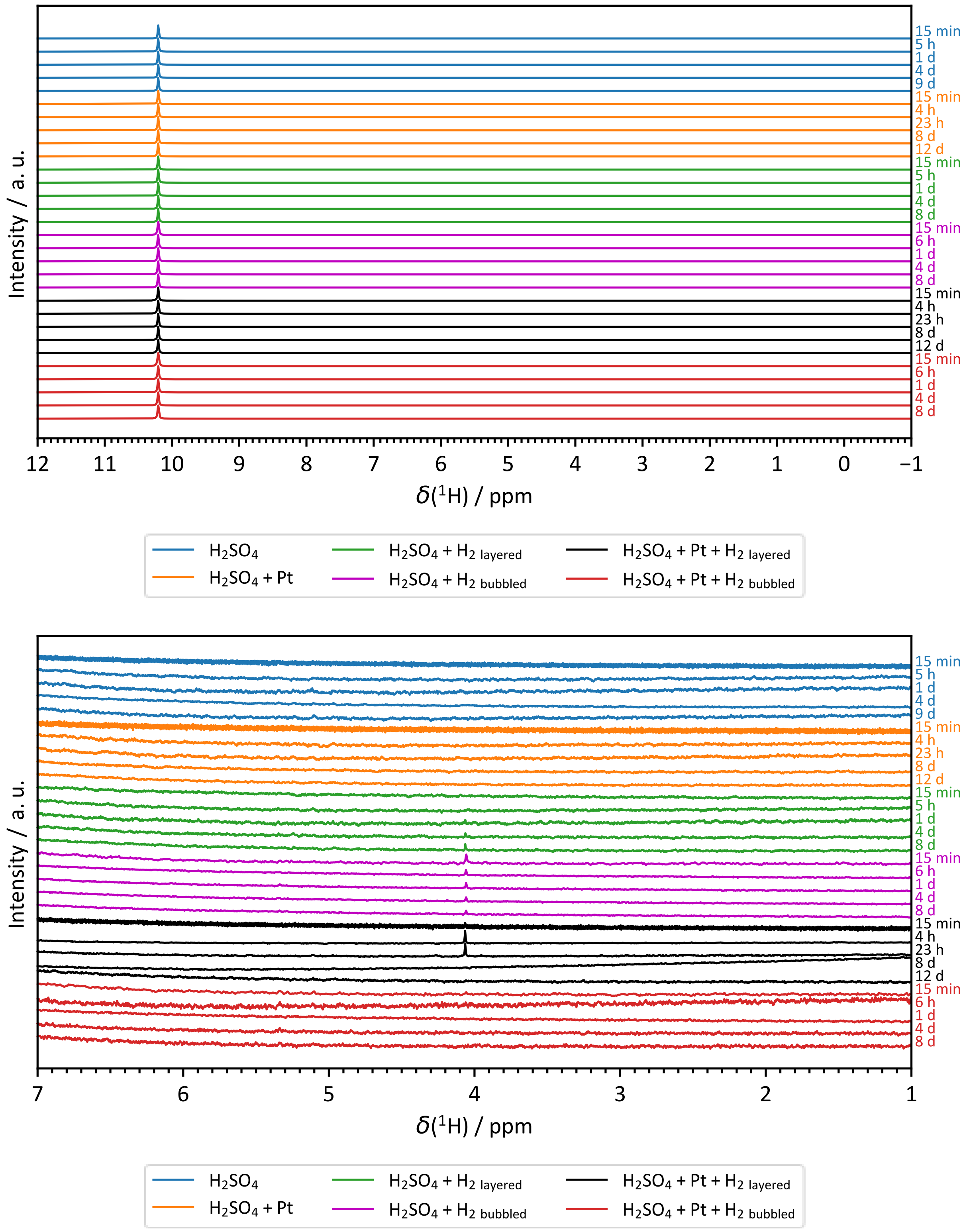

Figure S8 Top: ^{1}H-NMR data of the samples as indicated. Bottom: ^{1}H-NMR data of the samples as indicated around the H_2-Signal (4.06 ppm).

1.5 SSE Method

Ag | Ag_2SO_4 | Ag_2SO_4 (sat., S) ⋮ S ⋮ S | H_2 | Pt cell I

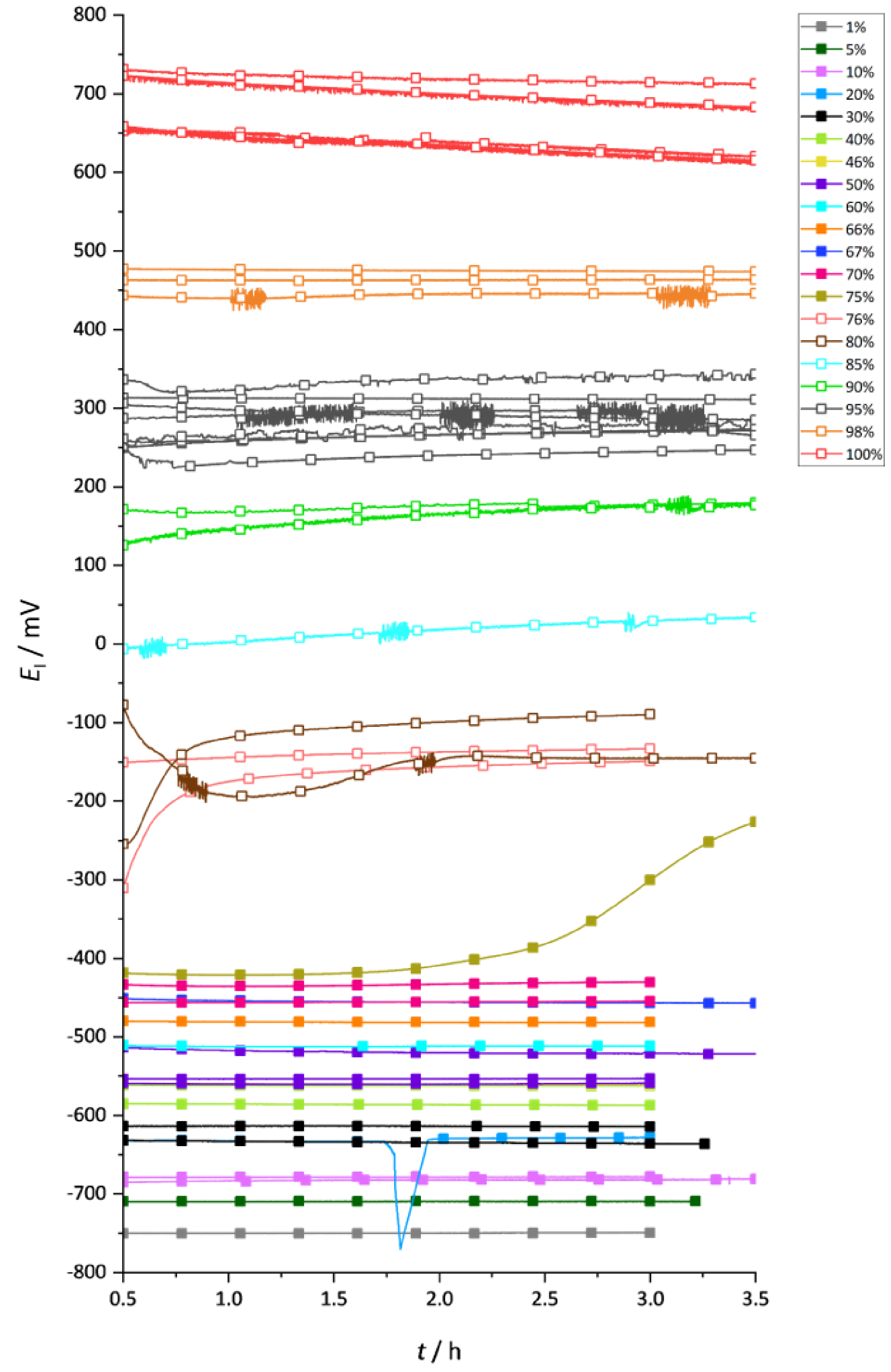

Figure S9 Measurement curves of cell I with different H_2O-H_2SO_4 mixtures as indicated.

The cell potentials were taken when a constant potential was achieved, for H_2SO_4 contents greater than 75 wt.-% at t = 3 h. The mean values of the measured cell potential $\bar{E}_I$ for each concentration are given in Table S4.

Table S4 Measured values E_I of cells I with different H_2SO_4 concentrations and the resulting $pH_{abs}^{H_2O}$ values.

S (wt.-% H_2SO_4)	c / mol•L^{-1}	E_I / mV	$E_{abs}^{H_2O}(H^+/H_2, S)$ / mV	$pH_{abs}^{H_2O}$
1	0.10	−749.0	−58.6	1.0
5	0.53	−709.1	−18.7	0.3
10	1.08	−679.8	10.6	−0.2
20	2.32	−628.0	62.4	−1.0
30	3.72	−624.7	65.7	−1.1
40	5.30	−574.5	115.9	−2.0
46	6.35	−560.5	129.9	−2.2
50	7.09	−544.1	146.3	−2.5
60	9.14	−511.7	178.7	−3.0
66	10.50	−481.4	209.0	−3.5
67	10.73	−456.7	233.7	−4.0
70	11.46	−442.3	248.1	−4.2
75	12.73	−300.5	389.9	−6.6
76	12.99	−144.8	545.6	−9.2
80	14.05	−117.5	572.9	−9.7
85	15.36	29.2	719.6	−12.2
90	16.60	176.0	866.4	−14.6
95	17.71	287.4	977.8	−16.5
98	18.30	461.4	1151.8	−19.5
100	18.61	662.6	1353.0	−22.9

1.6 ILSB Method

Pt | H_2 | H_2SO_4 (S_j) | [C$_8$MIm][FAP] | H_2SO_4 (S_i) | H_2 | Pt cell II

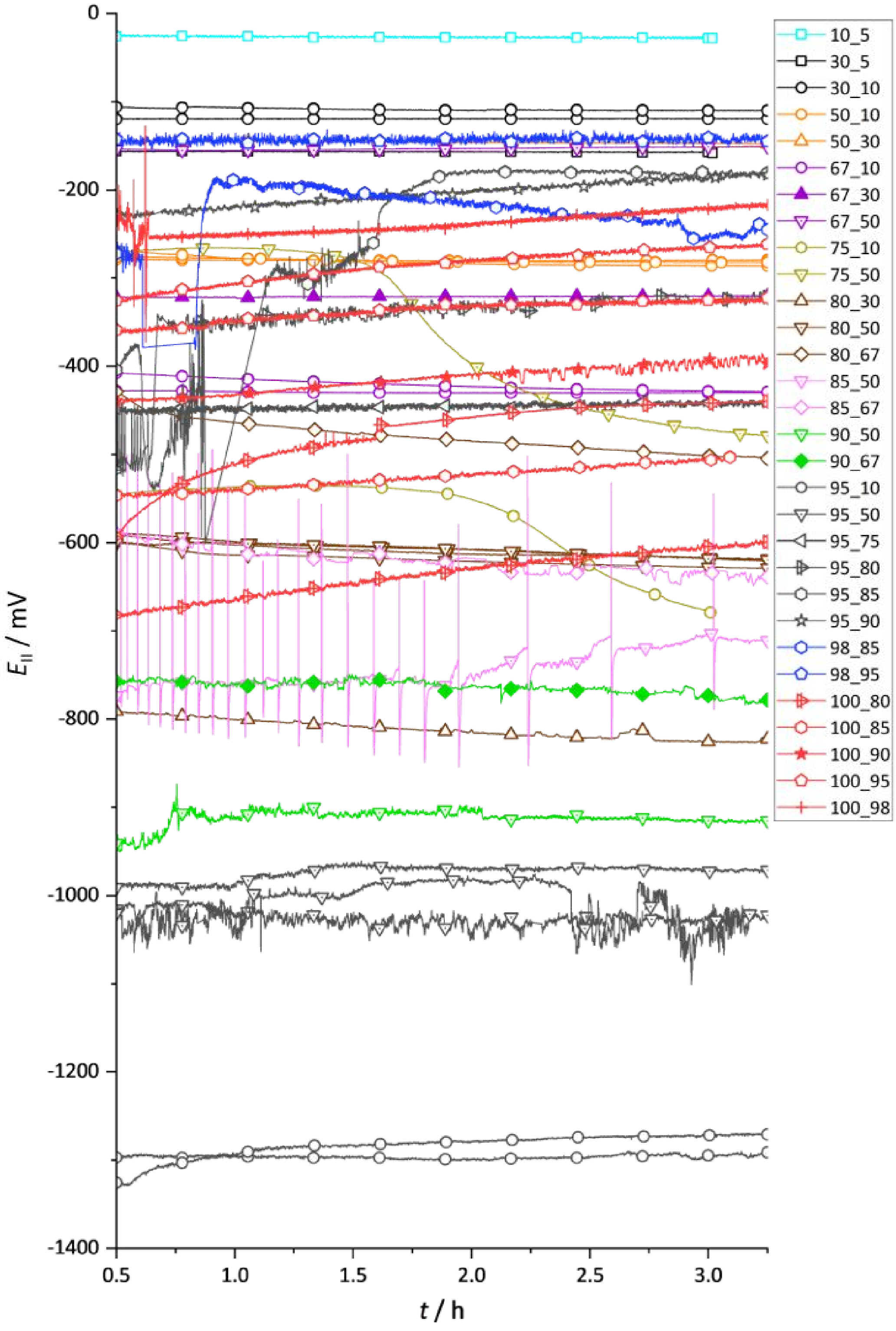

Figure S10 Measured values E_{II} of cells II with different H_2O-H_2SO_4 mixtures in the half-cells as indicated.

The cell potential values were taken when a constant potential was achieved, or at t = 3 h.

Network Analysis H_2SO_4 5 wt.-% to 100 wt.-%

The detailed procedure of the network analysis can be found elsewhere.[7,8] The first value of the input file is the measured value E_{II} in mV. The second and third value are the half-cells as indicated in the system list below where 1 means the half-cell with 5 wt.-% H_2SO_4, 2 with 10 wt.-% H_2SO_4 and so forth. As can be seen from the input file (and from Figure S10) some implementations of cell II where measured more than once.

Input file

```
43 12 equations, unknown variables
   -27.4   1  2
  -157.2   1  3
  -119.2   2  3
  -109.9   2  3
  -279.7   2  4
  -285.8   2  4
  -281.8   2  4
  -147.2   3  4
  -429.8   2  5
  -428.0   2  5
  -320.1   3  5
  -151.2   4  5
  -536.6   2  6
  -266.0   4  6
  -825.3   3  7
  -616.9   4  7
  -617.8   4  7
  -617.6   4  7
  -627.6   4  7
  -501.4   5  7
  -703.8   4  8
  -635.4   5  8
  -914.4   4  9
  -773.2   5  9
 -1295.1   2 10
 -1271.7   2 10
 -1025.6   4 10
  -971.2   4 10
 -1043.3   4 10
  -442.0   6 10
  -323.9   7 10
  -180.3   8 10
  -186.8   9 10
  -253.2   8 11
  -141.3  10 11
  -441.3   7 12
  -603.9   7 12
  -505.9   8 12
  -392.3   9 12
  -265.4  10 12
  -325.0  10 12
  -221.4  11 12
     0.0   1  1

systems list:
   1   5
```

```
  2   10
  3   30
  4   50
  5   67
  6   75
  7   80
  8   85
  9   90
 10   95
 11   98
 12  100
```

Output file

The output contains the coefficients matrix, which is solved to obtain the solution. The solution list contains the number of the half-cell is according the system list above (first column). The second column is the potential of the indicated half-cell with respect to half-cell 1 (in mV), and the last column repeats the H_2SO_4 content in wt.-% divided by 10. It follows a list, where each equation is numbered consecutively (first column) with the individual residual (in mV, second column) of the respective cells II (which of the half-cells are indicated in the last two columns). Finally, the maximum residual is given and the root means square (rms) of the solution.

```
 least squares electrochemical network analysis

         12  species
         43  equations

 rhs, coefficient matrix
  -27.4 -1  1  0  0  0  0  0  0  0  0  0  0
 -157.2 -1  0  1  0  0  0  0  0  0  0  0  0
 -119.2  0 -1  1  0  0  0  0  0  0  0  0  0
 -109.9  0 -1  1  0  0  0  0  0  0  0  0  0
 -279.7  0 -1  0  1  0  0  0  0  0  0  0  0
 -285.8  0 -1  0  1  0  0  0  0  0  0  0  0
 -281.8  0 -1  0  1  0  0  0  0  0  0  0  0
 -147.2  0  0 -1  1  0  0  0  0  0  0  0  0
 -429.8  0 -1  0  0  1  0  0  0  0  0  0  0
 -428.0  0 -1  0  0  1  0  0  0  0  0  0  0
 -320.1  0  0 -1  0  1  0  0  0  0  0  0  0
 -151.2  0  0  0 -1  1  0  0  0  0  0  0  0
 -536.6  0 -1  0  0  0  1  0  0  0  0  0  0
 -266.0  0  0  0 -1  0  1  0  0  0  0  0  0
 -825.3  0  0 -1  0  0  0  1  0  0  0  0  0
 -616.9  0  0  0 -1  0  0  1  0  0  0  0  0
 -617.8  0  0  0 -1  0  0  1  0  0  0  0  0
 -617.6  0  0  0 -1  0  0  1  0  0  0  0  0
 -627.6  0  0  0 -1  0  0  1  0  0  0  0  0
 -501.4  0  0  0  0 -1  0  1  0  0  0  0  0
 -703.8  0  0  0 -1  0  0  0  1  0  0  0  0
 -635.4  0  0  0  0 -1  0  0  1  0  0  0  0
 -914.4  0  0  0 -1  0  0  0  0  1  0  0  0
 -773.2  0  0  0  0 -1  0  0  0  1  0  0  0
-1295.1  0 -1  0  0  0  0  0  0  0  1  0  0
-1271.7  0 -1  0  0  0  0  0  0  0  1  0  0
-1025.6  0  0  0 -1  0  0  0  0  0  1  0  0
 -971.2  0  0  0 -1  0  0  0  0  0  1  0  0
-1043.3  0  0  0 -1  0  0  0  0  0  1  0  0
 -442.0  0  0  0  0  0 -1  0  0  0  1  0  0
 -323.9  0  0  0  0  0  0 -1  0  0  1  0  0
```

-180.3	0	0	0	0	0	0	0	-1	0	1	0	0
-186.8	0	0	0	0	0	0	0	0	-1	1	0	0
-253.2	0	0	0	0	0	0	0	-1	0	0	1	0
-141.3	0	0	0	0	0	0	0	0	0	-1	1	0
-441.3	0	0	0	0	0	0	-1	0	0	0	0	1
-603.9	0	0	0	0	0	0	-1	0	0	0	0	1
-505.9	0	0	0	0	0	0	0	-1	0	0	0	1
-392.3	0	0	0	0	0	0	0	0	-1	0	0	1
-265.4	0	0	0	0	0	0	0	0	0	-1	0	1
-325.0	0	0	0	0	0	0	0	0	0	-1	0	1
-221.4	0	0	0	0	0	0	0	0	0	0	-1	1
0.0	1	0	0	0	0	0	0	0	0	0	0	0

solution

i, potential (mV)

1	0.0	0.5
2	-34.5	1.0
3	-150.1	3.0
4	-318.2	5.0
5	-453.4	6.7
6	-664.9	7.5
7	-966.5	8.0
8	-1072.9	8.5
9	-1178.3	9.0
10	-1281.4	9.5
11	-1359.7	9.8
12	-1551.7	10.0

eq. no., residual (mV)

1	7.074	1	2
2	7.074	1	3
3	3.547	2	3
4	5.753	2	3
5	3.995	2	4
6	2.105	2	4
7	1.895	2	4
8	20.842	3	4
9	10.842	2	5
10	9.042	2	5
11	16.795	3	5
12	15.937	4	5
13	93.822	2	6
14	80.728	4	6
15	8.915	3	7
16	31.443	4	7
17	30.543	4	7
18	30.743	4	7
19	20.743	4	7
20	11.680	5	7
21	50.884	4	8
22	15.979	5	8
23	54.254	4	9
24	48.317	5	9
25	48.127	2	10
26	24.727	2	10
27	62.322	4	10
28	7.922	4	10
29	80.022	4	10
30	174.550	6	10

31	8.965	7	10
32	28.294	8	10
33	83.668	9	10
34	33.651	8	11
35	63.043	10	11
36	143.901	7	12
37	18.699	7	12
38	27.040	8	12
39	18.902	9	12
40	4.866	10	12
41	54.734	10	12
42	29.391	11	12
43	0.000	1	1

174.55016978133153 = maximum residual (mV)
59.853923783799807 = rms error (mV)

Table S5 Optimized values $\Delta_{tr}G°(H^+, S_1 \rightarrow S_2)$ as obtained from the network analysis (cf. Figure 3b). Measured values E_{II} of cells II with different H_2SO_4 concentrations and the resulting $pH_{abs}^{H_2O}$ values.

S (wt.-% H_2SO_4)	c / mol·L^{-1}	$E_{II,opt}$ / mV	$pH_{abs}^{H_2O}$
5	0.53	0	0.3
10	1.08	−34.5	−0.3
30	3.72	−150.1	−2.2
50	7.09	−318.2	−5.1
67	10.73	−453.4	−7.4
75	12.73	−664.9	−10.9
80	14.05	−966.5	−16.0
85	15.36	−1072.9	−17.8
90	16.60	−1178.3	−19.6
95	17.71	−1281.4	−21.4
98	18.30	−1359.7	−22.7
100	18.61	−1551.7	−25.9
m	*n*	*σ*	
42	12	59.9 mV	1.0 pH-units

2 Computational Details

2.1 General Aspects

The structures used for the gas phase thermodynamics were calculated with the ORCA 5.0.1 and 5.0.3 program packages.[9] Geometry optimizations were performed at the DSD-PBEP86[10]/def2-TZVPP[11] level of theory with the Resolution of Identity (RI) approximation and the def2/J[12] and def2-TZVPP/C[13] auxiliary basis sets. Additionally, the atom-pairwise dispersion correction with Becke-Johnson damping scheme (D3BJ)[14] was applied. The optimization utilized the smallest integration grid (DefGrid3) and all structures were identified as true minima by means of a numerical frequency calculation, which showed no imaginary modes. The geometries of the Hammett bases were optimized at the DSD-BLYP[15]/def2-TZVPP[11] level of theory with otherwise identical settings otherwise. The parameterization of both DSD-double-hybrid functionals is consistent with the GMTKN55 benchmark.[16]
These structures were then used for DLPNO-CCSD(T)[17] and CCSD(T) single point calculations. The energies were extrapolated to the complete basis set limit (CBS) through the cc-pVQZ[18,19] and cc-pV5Z [18,19] basis sets with the integrated extrapolation method (Extrapolate 4/5). The DLPNO approximation made use of the auxiliary basis cc-pV5Z/C[20] and tight PNO settings. Convergence tolerance was set to an energy change of 1.0^{-8} au (tightSCF).
The free energies of solvation were calculated via the COSMO-RS[21] theory, as implemented in BIOVIA COSMO*therm* 2023.[22] The structures were optimized with TURBOMOLE 7.5.1[23] through the COSMO*conf* program, which is part of the COSMO*therm* suite, in order to use its isomer search. All found isomer structures were calculated with the default settings for the *BP-TZVPD-FINE* parameterization (RI-DFT BP86[24]/def2-TZVPD[11] single point with a fine grid marching tetrahedron cavity[25] on a BP86/def-TZVP[11] optimized structure).
Additional solvation calculations for the Hammett Bases were performed with ORCA and the CPCM[26] solvation model. The geometry optimizations and single-point calculations used identical settings as the gas-phase calculations with the Gaussian charge scheme (*vdw_gaussian* surface type) and a dielectric constant of 110.[27] The single-points utilized the *CPCMccm 2* scheme.

2.2 Pure Sulfuric Acid

Pure sulfuric acid has been studied extensively and served as a starting point for these calculations. Even in its anhydrous state, it still contains trace amounts of ions because of several dissociation processes. The main dissociation equilibrium is the autoprotolysis to $H_3SO_4^+$ and HSO_4^-, along with a minor self-dehydration, that is accompanied by an ionization of both products to H_3O^+ and $HS_2O_7^-$.[28,29] Different methods have determined a variety of values for both constants.[30] Gillespie *et al*. used cryoscopy to determine the constants for both dissociation processes at 298.15 K, which were selected for the calculations in this work.[28] These autoprotolysis constant K_{ap} and constant of self-dehydration K_{ip} as well as their pK_{ap} and pK_{ip} values, are provided in Eqs S3 and S4, respectively:

$$K_{\mathrm{ap}} = 8.0 \cdot 10^{-4}\ \mathrm{mol^2 L^{-2}}\ (\mathrm{p}K_{\mathrm{ap}} = 3.10), \quad \text{S3}$$

$$K_{\mathrm{ip}} = 1.4 \cdot 10^{-4}\ \mathrm{mol^2 L^{-2}}\ (\mathrm{p}K_{\mathrm{ip}} = 3.85). \quad \text{S4}$$

The autoprotolysis constant ensues a concentration of 0.028 mol L^{-1} for both HSO_4^- and $H_3SO_4^+$ and the self-dehydration constant results in 0.012 mol L^{-1} for H_3O^+ and $HS_2O_7^-$, respectively. All species besides these four ions and H_2SO_4 are included at infinite dilution, which is ultimately a mole fraction of zero, meaning they do not impact the overall composition of the mixture or the resulting solvation energies. Figure S11 shows the optimized gas-phase structures of all the species added to the mixture, as determined at the DSD-PBE86/def2-TZVPP level of theory.

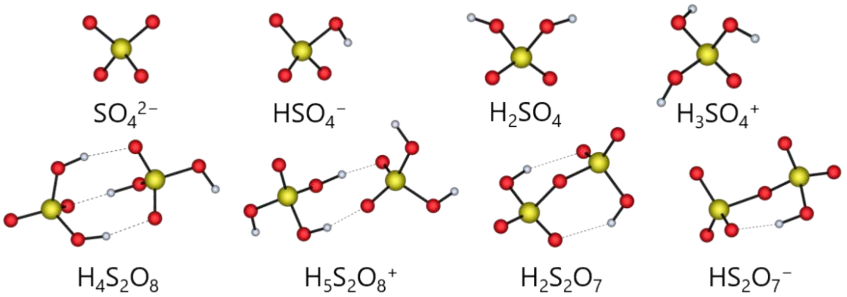

Figure S11 Optimized DSD-PBEP86/def2-TZVPP gas-phase structures of the different sulfuric acid derivatives used to calculate the solvation energies of the proton in pure sulfuric acid.

Where experimental gas-phase basicities were available, they were in good agreement with the DLPNO-CCSD(T)/CBS results. The calculated GB for H_2SO_4 was 684.7 kJ mol^{-1}, which closely matches the experimental value of 683 ± 3 kJ mol^{-1}.[31] The HSO_4^- ion shows a small deviation with a calculated GB of 1270.5 kJ mol^{-1}, compared to the value of 1265.2 kJ mol^{-1} reported in the literature.[32] Table S19 summarizes all gas-phase basicities at the DSD-PBEP86 and DLPNO-CCSD(T) level of theory. Coupled cluster calculations without the DLPNO approximation, CCSD(T)/CBS were only feasible for the gas-phase basicities of the three water clusters $(H_2O)_{n=1–3}$, which are also included in Table S19. These three energies differ by less than 0.5 kJ mol^{-1}, which demonstrates the excellent performance of the DLPNO, even for non-covalently bound systems, albeit very small ones.

The structures acquired from COSMO calculations, which are required as input for COSMO-RS, may show notable differences and additional isomers compared to the gas-phase structures because of the different theoretical methods and the inclusion of the solvation model COSMO. Nevertheless, in certain cases, the level of theory and integration grid settings required by COSMOtherm's parameterization are insufficient for a subsequent frequency calculation. Consequently, these structures are not illustrated in this work. Since the program is designed to use these specific optimization settings with additional parameters and to prioritize, among other properties, solvation energies, the actual COSMO structures become secondary in this approach.

Apart from H_2SO_4, $H_3SO_3^+$, HSO_4^- and the sulfate anion SO_4^{2-}, which is in fact only present in diluted aqueous solutions, these structures mainly arise from different adduct formations or dimerizations in pure sulfuric acid. The ion $H_5S_2O_8^+$ is considered an adduct of sulfuric acid and $H_3SO_4^+$ and $H_3S_2O_8^-$ is a combination of sulfuric acid and HSO_4^-. Including more species allows for the application of specific thermodynamic cycles, such as adding SO_4^{2-} to complete the cycle with HSO_4^- or H_2O to complete the cycle with H_3O^+. Therefore, besides species that were reasonable approximations of the pure sulfuric acid environment or supported by experimental data, certain species were also included to close specific thermodynamic cycles. Figure S12 illustrates the thermodynamic cycle that is employed to calculate the Gibbs energy of solvation of the proton $\Delta_{solv}G(H^+, H_2SO_4)$ in pure sulfuric acid using the components of the modelled sulfuric acid denoted as E and EH^+.

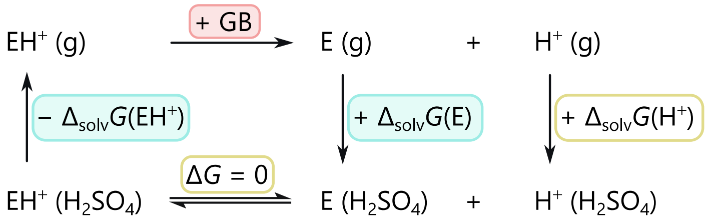

Figure S12 Thermodynamic cycle to calculate the Gibbs energy of solvation of the proton $\Delta_{solv}G(H^+, H_2SO_4)$ through the solvation energies of the species E and EH^+ that are both components of the sulfuric acid modeled with COSMOtherm as well as the gas-phase basicity of E.

The label E represents any neutral, positively or negatively charged entity present in pure sulfuric acid, thus EH^+ is not necessarily positively charged. The top portion of the cycle depicts the gas-phase basicity of species E at 1 bar and the calculated solvation energies of both E and EH^+ describe the transition from 1 bar

gas into an ideal 1 mol L^{-1} solution. However, if the bottom reaction is assumed to have reached equilibrium (ΔG = 0), the concentrations of all species deviate from the ideal 1 mol L^{-1}. This means that the solvation energy of the proton $\Delta_{solv}G(H^+, H_2SO_4)$ that results from this cycle is no longer a standard state of 1 mol L^{-1}, either. Instead, it refers to the pH of neutral sulfuric acid defined through the dissociation processes mentioned above, which are included in the modelled mixture. This state is consistent with the experimental results obtained from both the SSE and ILSB measurements. In order to convert the proton's solvation energy to standard conditions of an ideal solution with 1 mol L^{-1} ($pH_{H_2SO_4}$ = 0), it is necessary to subtract the pH of pure sulfuric acid. This $pH_{H_2SO_4}$ value is calculated from the ions resulting from the self-dehydration and autoprotolysis according to −log(0.028 + 0.012) = 1.40, which corresponds to 8 kJ mol^{-1}. Applying this adjustment to convert the experimental results to standard conditions would shift the ILSB value from −25.9 to −27.3 and the SSE value from −22.9 to −24.3. Both the calculated and experimental solvation energies refer to a $pH_{abs}^{H_2O}$ of 1.4, which is the correct definition of the superacidity threshold and do not require additional adjustments. Thus, the proton's solvation energy $\Delta_{solv}G(H^+, H_2SO_4)$ at the $pH_{H_2SO_4}$ of 1.4 is given by

$$\Delta_{solv}G(H^+, H_2SO_4) = -GB + \Delta_{solv}G(EH^+, H_2SO_4) - \Delta_{solv}G(E, H_2SO_4). \qquad S5$$

Table S6 provides the calculated $\Delta_{solv}G(H^+, H_2SO_4)$ and $pH_{abs}^{H_2O}$ values for each species E/EH+, depicted in Figure S11 , along with the energies needed to complete the thermodynamic cycles according to Eq S5, such as the GB and the solvation energies of both E and EH+s.

Table S6 All calculated energies required to complete the thermodynamic cycle in Figure Figure S11 to calculate the solvation energy of the proton $\Delta_{solv}G(H^+, H_2SO_4)$ and the respective absolute acidity $pH_{abs}^{H_2O}$. These energies comprise the solvation energy of the protonated species E $\Delta_{solv}G(EH^+)$, the solvation energy of species E $\Delta_{solv}G(E)$ and the GB of species E. EH^+ represents the protonated form of E and does not always carry a positive charge. The solvation energies as well as the $pH_{abs}^{H_2O}$ values are not under standard conditions, but at the $pH_{H_2SO_4}$ of neutral sulfuric acid of 1.4.

EH^+		$\to H^+ + E$	GB kJ mol^{-1}	$\Delta_{solv}G(EH^+)$ kJ mol^{-1}	$\Delta_{solv}G(E)$ kJ mol^{-1}	$\Delta_{solv}G(H^+, H_2SO_4)$[a] kJ mol^{-1}	$pH_{abs}^{H_2O}$[a]
HSO_4^-	→	$H^+ + SO_4^{2-}$	1860.1	–310.3	–1216.0	−954.5	−26.3
$H_3SO_4^+$	→	$H^+ + H_2SO_4$	684.7	–296.0	–41.6	−939.2	−29.0
$H_2S_2O_7$	→	$H^+ + HS_2O_7^-$	1172.2	−36.6	−220.7	−988.1	−20.4
H_3O^+	→	$H^+ + H_2O$	656.9	–337.7	–32.1	–962.5	−24.9
$H_5S_2O_8^+$	→	$H^+ + H_4S_2O_8$	732.7	–279.3	–54.1	−957.9	−25.7
$H_5S_2O_8^+$	→	$H^+ + 2\ H_2SO_4$	753.8	–279.3	–83.2	−949.9	−27.1
$H_4S_2O_8$	→	$H^+ + H_3S_2O_8^-$	1154.5	–54.1	–230.2	−978.4	−22.1
median						−957.9	−25.7

[a] 1.4 or 8 kJ mol^{-1} have to be subtracted to obtain standard conditions of an ideal solution of 1 mol L^{-1} ($pH_{H_2SO_4}$ = 0)

Similar to the calculations in part one, it is crucial to understand the precise manner in which the proton exists in this mixture. The focus here is not on the exact structure, but on the most likely species EH^+ that represents the chemical potential of the proton through the described thermodynamic cycle. The calculated energies cover a range of nearly 9 pH units with a median of −25.7. Given that the two experimental values differ by 3 pH units and each method has an estimated uncertainty of 2 to 3 pH units, most of the calculated values fall within this range. The outlier with the highest $pH_{abs}^{H_2O}$ of −29.0 is the cycle with the $H_3SO_4^+$ ion. Among the different species, the $pH_{abs}^{H_2O}$ of the sulfuric acid dimer $H_4S_2O_8$ closely matches the experimental SSE value of −22.9, while the $pH_{abs}^{H_2O}$ resulting from the $H_4S_2O_8^+$ cycle corresponds to −25.9 from the ILSB method.

It is important to note that these thermodynamic cycles are not equivalent and cannot result in the same absolute acidities. Moreover, the continuum model, which serves as the foundation for the COSMO-RS

theory, improves in accuracy as the solute size increases, as shown in part one. Consequently, the solvation energies of the species $H_5S_2O_8^+$ and $H_4S_2O_8$ are more precise, while the accuracy of the gas-phase contribution remains consistently good, as long as the size of the species remains relatively small. Moreover, pure sulfuric acid is highly viscous and creates a strong hydrogen bond network.[33] The three species at the bottom of Table Table S6 , which form hydrogen bonded adducts with sulfuric acid, contribute to increased viscosity due to their larger size and have a greater likelihood of affecting the proton's chemical potential. Their $pH_{abs}^{H_2O}$ values lie between −22.1 and −27.1, close to both experiments. Additional information from the evaluation of the modeled aqueous sulfuric acid in Section 2.3 supports the sulfuric acid adducts and the sulfuric acid dimer as the most probable species to determine the chemical potential. Depending solely on these calculations does not reliably identify the optimal thermodynamic cycle or the crucial species that determines the proton's chemical potential, especially when the experimental values themselves vary greatly. Supplementary information about the medium's composition and other characteristics, such as viscosity, is crucial for achieving the most precise estimate of its acidity. The proton's chemical potential might be distributed across multiple species in a complex equilibrium, which complicates this quantum chemical approach that needs a defined species to apply the thermodynamic cycle. The closely clustered $pH_{abs}^{H_2O}$ values indicate that this might be the case. However, the most important aspect of these results is that even the lowest $pH_{abs}^{H_2O}$ value of −20.4 from the $H_2S_2O_7$ cycle is still eight orders of magnitude greater than the H_0 value of −11.93, supporting the new acidity measurements[34] in the conclusion that the Hammett function severely underestimates the acidity of pure sulfuric acid.

Nevertheless, this approach introduces ambiguity surrounding the species that determines the chemical potential of the proton and thus the acidity, leading to uncertainty in the calculated $pH_{abs}^{H_2O}$ values. The upcoming section deals with the uncertainty caused by the lack of knowledge about potential determining species.

2.2.1 Indicator Base Approach to determine Gibbs Energies of Proton Solvation

This approach uses the pure sulfuric acid model described above, but the species E and EH^+ are no longer used to calculate the proton's solvation energy in pure sulfuric acid $\Delta_{solv}G(H^+, H_2SO_4)$. Instead, an additional weak indicator base B and its protonated form BH^+ are incorporated into the cycle. Gillespie and colleagues used multiple indicator bases, following Hammett's approach, to determine the H_0 value of pure sulfuric acid.[35] These bases were added at infinite dilution to the previously described pure sulfuric acid model and their standard solvation energies, $\Delta_{solv}G°(B, H_2SO_4)$ and $\Delta_{solv}G°(BH^+, H_2SO_4)$.

The main benefit of this approach is that it no longer requires knowledge about the potential determining species and unlike the cycles involving various E/EH^+ species, cycles using the bases B/BH^+ are all equivalent and should, at least in theory, lead to the same outcome. Moreover, this approach does not require in-depth understanding of the system's composition and the precise solvation structure of proton. This comes with the benefit of a simple continuum model, such as CPCM, being an appropriate alternative to the more intricate COSMO-RS approach. Another advantage is that the aromatic indicator bases are larger molecules compared to the chemical entities E and EH^+ found in sulfuric acid. They are less prone to strong '*first solvation shell effects*' that small and highly charged ions experience. Furthermore, while sulfuric acid forms a robust hydrogen bond network, it is not as strong as that observed in certain other solvents, such as water. Consequently, the solvation energies of the indicator bases and their protonated counterparts were also calculated with the continuum model CPCM with a relative permittivity ε_r of 110.[27] The thermodynamic cycle required for this approach is depicted in Figure S13, with the species B as the neutral indicator base and BH^+ as its protonated form.

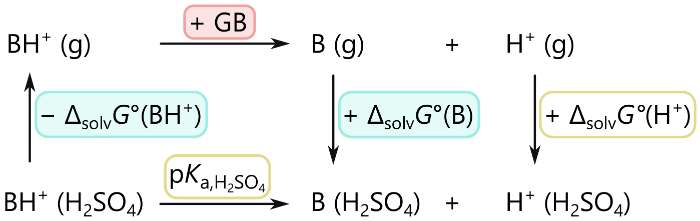

Figure S13 Thermodynamic cycle to determine the Gibbs energy of solvation of the proton $\Delta_{solv}G°(H^+, H_2SO_4)$ under standard conditions (pH = 0) in pure sulfuric acid through an indicator base B and the $pK_{a,H_2SO_4}(BH^+, H_2SO_4)$ of the respective protonated form BH^+.

The distinction from the previous thermodynamic cycle lies in the bottom reaction, which is no longer in equilibrium. Now, the energy difference is described by the experimental dissociation constant K_a of the protonated indicator base BH^+ in pure sulfuric acid, which remains constant even as concentrations change, allowing for the use of various concentrations. Therefore, all species exist in the standard state of 1 mol L^{-1} because they originate from the gas-phase, which is at a standard state of 10^5 Pa. This results in a standard solvation energy of the proton $\Delta_{solv}G°(H^+, H_2SO_4)$ at a concentration of 1 mol L^{-1}, which has to be adjusted by 1.4 pH values for the state of neutral sulfuric acid.
The $pK_{a,H_2SO_4}(BH^+, H_2SO_4)$ values of the protonated indicators that were used to describe the energy difference in the bottom reaction were not directly measured in pure sulfuric acid, but adjusted with the $pH_{H_2SO_4}$ and the H_0 value. Assuming the concentration of the indicator base is negligibly low, the $pH_{H_2SO_4}$ remains at 1.40. With the experimental $pK_{a,H_2O}(BH^+, H_2SO_4)$ values that reference water as their standard state[35] the $pK_{a,H_2SO_4}(BH^+, H_2SO_4)$ values in the reference state of pure sulfuric acid were derived according to

$$pK_{a,H_2SO_4}(BH^+, H_2SO_4) = pK_{a,H_2O}(BH^+, H_2SO_4) + pH_{H_2SO_4} - H_0(H_2SO_4),$$
$$pK_{a,H_2SO_4}(BH^+, H_2SO_4) = pK_{a,H_2O}(BH^+, H_2SO_4) + 1.40 - 11.93. \qquad S6$$

Table S7 summarizes the $pK_{a,H_2O}(BH^+, H_2SO_4)$ values determined by Gillespie *et al.*[35] and the calculated $pK_{a,H_2SO_2}(BH^+, H_2SO_4)$ values for each indicator base, together with the resulting dissociation energies $\Delta G^a = pK_{a,H_2SO_2}(BH^+, H_2SO_4)RT\ln(10)$.

Table S7 The $pK_{a,H_2O}(BH^+, H_2SO_4)$ values of the indicator bases, which refer to water as the standard state[35] and the thermodynamically consistent $pK_{a,H_2SO_2}(BH^+, H_2SO_4)$ values with the reference state of pure sulfuric acid as well as the dissociation energies ΔG^a calculated from them.

Base B	$pK_{a,H_2O}(BH^+, H_2SO_4)$	$pK_{a,H_2SO_2}(BH^+, H_2SO_4)$	ΔG^a kJ mol^{-1}
2,4,6-trinitroaniline	−10.10	3.23	18.44
p-nitrotoluene	−11.35	1.98	11.30
m-nitrotoluene	−11.99	1.34	7.65
nitrobenzene	−12.14	1.19	6.79
p-nitrofluorobenzene	−12.44	0.89	5.08
p-nitrochlorobenzene	−12.70	0.63	3.60
m-nitrochlorobenzene	−13.20	0.13	0.74
2,4-dinitrotoluene	−13.74	−0.41	−2.34

The Gibbs energy of solvation of the proton $\Delta_{solv}G°(H^+, H_2SO_4)$ under standard conditions can be calculated from Eq S6.

$$\Delta_{solv}G°(H^+, H_2SO_4) = -GB + \Delta_{solv}G°(BH^+, H_2SO_4) - \Delta_{solv}G°(B, H_2SO_4) - \Delta G^a. \qquad S7$$

Table 1 in the main text summarizes all the energies required to complete the thermodynamic cycle illustrated in Figure S13, according to Eq S7, together with the resulting proton solvation energies $\Delta_{solv}G°(H^+,$

H_2SO_4) under standard conditions and the absolute acidities $pH_{abs}^{H_2O}$, which are corrected by 1.4 pH values for the state of neutral sulfuric acid. The table includes the gas-phase basicities of the indicators B as well as the solvation energies of all species B and BH^+ computed with both COSMO-RS and CPCM, which are enclosed in brackets and listed below the COSMO-RS energies. Table S8 contains the corresponding base transfer terms.

Table S8 Solvation energies $\Delta_{solv}G°$ of the Hammett bases in sulfuric acid with the constants of autoprotolysis K_{ap} and self-dehydration K_{ip} obtained with COSMOtherm in kJ mol^{-1}. They are used to obtain $\Delta_{solv}G°(H^+, H_2SO_4)$ as given in Table 1 in the main text. Further, they are used with Eq. 6b (as sB and sBH^+) together with wB = p-nitroaniline, $\Delta_{solv}G°(wB, H_2O) = -25$ kJ mol^{-1} and $\Delta_{solv}G°(wBH^+, H_2O) = -302$ kJ mol^{-1}.

Base B	$\Delta_{solv}G°(B, H_2SO_4)$	$\Delta_{solv}G°(BH^+, H_2SO_4)$	$\Delta\Delta_{tr}G°(B - BH^+, H_2O \rightarrow H_2SO_4)$
2,4,6-trinitroaniline	−61.48	−287.13	−51.4
p-nitrotoluene	−31.20	−232.86	−75.3
m-nitrotoluene	−28.65	−234.52	−71.2
nitrobenzene	−27.11	−239.67	−64.4
p-nitrofluorobenzene	−25.62	−241.35	−61.2
p-nitrochlorobenzene	−26.94	−239.47	−64.4
m-nitrochlorobenzene	−24.43	−244.53	−56.9
2,4-dinitrotoluene	−44.39	−260.11	−61.3
		median	−62.9

Naturally, these results are more consistent compared to the energies gained from the different species, E and EH^+, present in the acid. COSMO-RS theory determines a median $pH_{abs}^{H_2O}$ of −21.5 with values spanning from −21.0 to −25.7. The median $pH_{abs}^{H_2O}$ from the CPCM calculations is −22.1, with a range of −22.0 to −24.9. The medians of both methods are only 0.5 pH units apart, but the range of $pH_{abs}^{H_2O}$ values obtained with COSMO-RS covers 4.7 pH units, whereas the CPCM values cover only 2.9 pH units. Since the indicator bases have similar aromatic structures, their description by the pure continuum model is more uniform. However, the parameterization of COSMO-RS introduces additional terms to account for the interactions with the solvent that are not included in the CPCM. This may contribute to a higher dispersion of the calculated solvation energies of the proton $\Delta_{solv}G°(H^+, H_2SO_4)$. It should be noted that the proton's solvation energies include a substantial amount of error cancellation, as the calculated individual solvation energies of the bases and their protonated forms differ noticeably. Within the same method, there is a difference of approximately 50 kJ mol^{-1} between the highest and the lowest solvation energy of the protonated bases BH^+. While the general structures of these bases are similar, the presence of different functional groups leads to some variations in their solvation energies. In both methods, the highest $pH_{abs}^{H_2O}$ value comes from the cycle incorporating 2,4,6-trinitroaniline. This compound was the indicator base used by Hammett in his original research to determine the H_0 function of pure sulfuric acid.[36] Unlike the other aromatic nitrobenzene and nitrotoluene compounds examined in this study, 2,4,6-trinitroaniline does not undergo protonation on an oxygen atom. Figure S14 shows the optimized gas-phase structures of the indicators B and their protonated counterparts at the DSD-BLYP/def2-TZVPP level of theory.

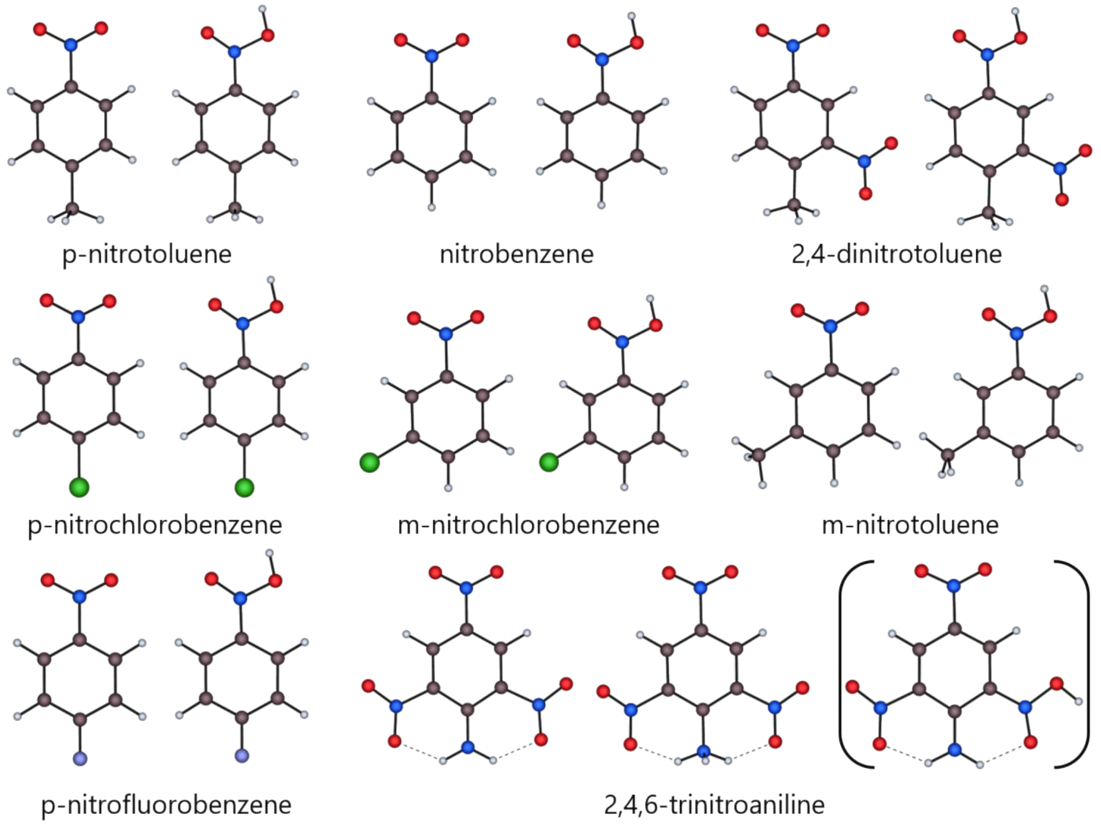

Figure S14 Optimized DSD-BLYP/def2-TZVPP gas-phase structures of the indicator bases and their protonated forms used to calculate the solvation energies of the proton in pure sulfuric acid. The bracketed structure of protonated 2,4,6-trinitroaniline was excluded from further calculations.

The 2,4,6-trinitroaniline indicator is stabilized by two intramolecular hydrogen bonds and is protonated on the NH_2-group instead of NO_2-group. The protonation of the NO_2-group in the 2,4,6-trinitroaniline is energetically less favorable, both in the gas-phase and within the CPCM, and was excluded from further calculations. Gillespie *et al.*[35] discussed that there might be a higher level of uncertainty associated with the mix of aromatic nitro compounds and 2,4,6-trinitroaniline, since the activity coefficient ratio of these different types of indicators is less likely to reach unity, as required by the Hammett assumption, although this requirement was satisfactorily met in their experiment. However, the computational approach used in this work may not follow the same principles. Apart from the differences mentioned earlier in 2,4,6-trinitroaniline and its protonated form, the presence of multiple nitro groups greatly affects the solvation energies of the indicators. When compared to the COSMO-RS calculations of the other bases, which have solvation energies between −25 and −30 kJ mol^{-1}, both 2,4-dinitrotoluene and 2,4,6-trinitroaniline show higher solvation energies, measuring −44.4 kJ mol^{-1} and −61.5 kJ mol^{-1}, respectively. The same applies to the protonated forms of the respective bases with an increase of the solvation energy with the number of nitro groups. This trend was also observed in a study that combined experimental and theoretical methods to analyze the hydration energies of multifunctional aromatic nitro compounds.[37] The CPCM calculations show the same pattern, albeit not as pronounced. The solvation energies are slightly influenced by other functional groups, such as the CH_3 group and their general positions. [37] These small differences cancel once the energies pass through the thermodynamic cycles, while the impact of several NO_2-groups prevails in the resulting $pH_{abs}^{H_2O}$ values.

To ensure the equivalence of thermodynamic cycles, it is crucial to use indicator bases that have similar structures and sizes to prevent significant fluctuations in the solvation energies of the bases and the proton. Consequently, the set of indicator bases used for the theoretical approach might need additional adjustments and cannot be transferred directly from the experimental measurements. Excluding 2,4-dinitrotoluene and 2,4,6-trinitroaniline, which is eventually accomplished by the median, leaves a very

consistent set with comparable proton solvation energies. Hammett's approach requires indicator bases that allow for an accurate determination of the protonation ratios B/BH$^+$. Yet, it may not always be possible to exclude certain functional groups, but the computational method is not restricted by this limitation, allowing to add or remove certain functional groups to create a more homogeneous set of indicators.

There is a distinction between the two solvation models, particularly regarding the solvation energies of the neutral bases. While the solvation energies of the protonated bases show little variation between the solvation models, the solvation energies of the neutral bases exhibit significant differences, particularly given their overall small magnitude. Continuum solvation models cannot be fitted to represent charged and neutral solutes equally well. The CPCM is better suited for solutes that carry a charge, rather than neutral species. Ultimately, these variations cancel in the thermodynamic cycle, resulting in highly consistent proton solvation energies across all indicator bases and both solvation models. Although the CPCM may be less accurate, when applied to both sides of the cycle, the error cancellation reveals satisfactory outcomes.

Importantly, all $pH_{abs}^{H_2O}$ values, including the outliers, predict a considerably higher acidity for pure sulfuric acid than that predicted by the H_0 value, which was determined using the same indicators as those used in this work. This agrees with the results of the previous method, which led to the same conclusion, albeit with a higher uncertainty and variations in the solvation energies. In contrast, this approach offers a widely applicable method for acquiring accurate acidities by assessing the Gibbs solvation energies of the respective indicator base system B/BH$^+$ in various acids and acidic solvent mixtures. If this method applies to pure sulfuric acid with its relatively high ionic strength resulting from the autoprotolysis and self-dehydration, this should also apply to other acids or mixtures.

2.3 H_2O-H_2SO_4 Mixtures

The exact composition of aqueous sulfuric acid solutions is not known in its entirety, thus additional assumptions were made in order to create a theoretical description. The calculations started with the 5% concentration, which is the first point of the ILSB measurement. As H_2SO_4 dissociates into SO_4^{2-} and HSO_4^-, the resulting proton is incorporated into different protonated water clusters $(H(H_2O)_n)^+$, which decrease in size as the water concentration decreases. Since the exact concentrations and compositions of those water clusters are not fully known, approximations were used to maintain charge neutrality and ensure balanced molar masses in the mixture. Protonated water clusters with *n* values of 1–5 were included in the model. Clusters with higher values of *n* were not considered because they exist in highly diluted solutions and higher concentrated sulfuric acid was the main point of interest. Furthermore, test calculations showed that including $H_9O_4^+$ and $H_{11}O_5^+$ had no significant impact on the calculated solvation energies of the proton.

Young *et al.* determined the concentration of SO_4^{2-} and HSO_4^- through Raman spectroscopy.[38] They found that the first dissociation to HSO_4^- is incomplete. At a concentration of 13.9 mol L^{-1} sulfuric acid (79.4%), the maximum concentration of HSO_4^- is reached with only 13.6 mol L^{-1}. The dissociation to HSO_4^- requires excess water, so that an exclusive 1 to 1 mixture of H_3O^+ and HSO_4^- is never realized.[39,38] The concentration of SO_4^{2-} progresses in a parabolic fashion and reaches its maximum of 1.8 mol L^{-1} at 6–7.8 mol L^{-1} of sulfuric acid. These experimentally determined concentrations of HSO_4^- and SO_4^{2-} were approximated by polynomial regression curves of 8th and 6th order, respectively. Figure S15 shows the experimental concentrations in mol L^{-1} with the polynomial regression curves.

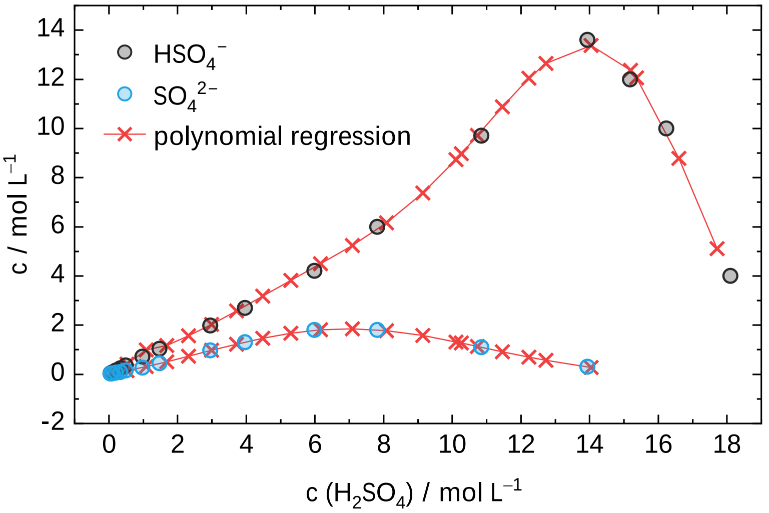

Figure S15 Concentrations of SO_4^{2-} and HSO_4^- in mol L^{-1} obtained from Raman measurements[39] plotted against the concentration of sulfuric acid in mol L^{-1} with polynomial regression curves fitted to both data sets.

Ultimately, the concentration profile of the first mixture was calculated using these fitted curves and later adjusted to lower dissociation degrees of sulfuric acid for the second and third mixture. All additional information on the regression curves is summarized in Section 3.1. The concentrations were calculated at intervals of 5% sulfuric acid ranging from 5% to 100% with additional points that were considered in the experiment, such as 67% and 98%. Other percentages that were calculated are 64.5%, 73.1%, and 84.5% and represent specific ratios of water to sulfuric acid, namely 3 to 1, 2 to 1, and 1 to 1, respectively. These unique points also correspond to the approximate locations of the concentration maxima for $H_7O_3^+$, $H_5O_2^+$, and H_3O^+.

Species other than the protonated water clusters $(H(H_2O)_n)^+$, H_2O, H_2SO_4, HSO_4^-, SO_4^{2-} exist in the calculated aqueous sulfuric mixtures only at infinite dilution. All species mentioned in Section 2.2 are accounted for in the aqueous mixture at infinite dilution. Typically, even species that eventually reach a concentration of zero are still present in the mixture at infinite dilution. This allows for the calculation of solvation curves that cover the entire concentration range, including pure sulfuric acid, which remains otherwise unchanged. It should be mentioned that determining the solvation energy of a species for the entire concentration range does not imply its existence at every concentration.

Figure S16 shows all optimized gas-phase structures at the DSD-PBE86/def2-TZVPP level of theory that were, besides the structures in Figure S11 , used to model the aqueous sulfuric acid mixtures.

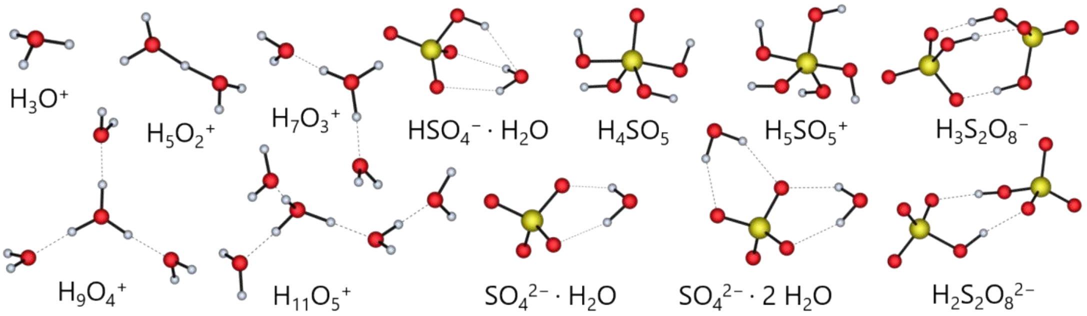

Figure S16 Optimized DSD-PBEP86/def2-TZVPP gas-phase structures of the sulfuric acid species used to calculate the solvation energies of the proton in aqueous sulfuric acid.

The water adducts of HSO_4^- and SO_4^{2-} were added at infinite dilution, eliminating the need for any further assumptions regarding their precise concentrations and dependence on the concentration of the protonated water clusters. In this work, all percentages are expressed as mass percent.

2.3.1 First Approximation of the Composition of Aqueous Sulfuric Acid

The initial approximation closely matches the concentrations derived from the polynomial regression curves to the Raman data[38] leading to the concentration profile illustrated in Figure S17 .

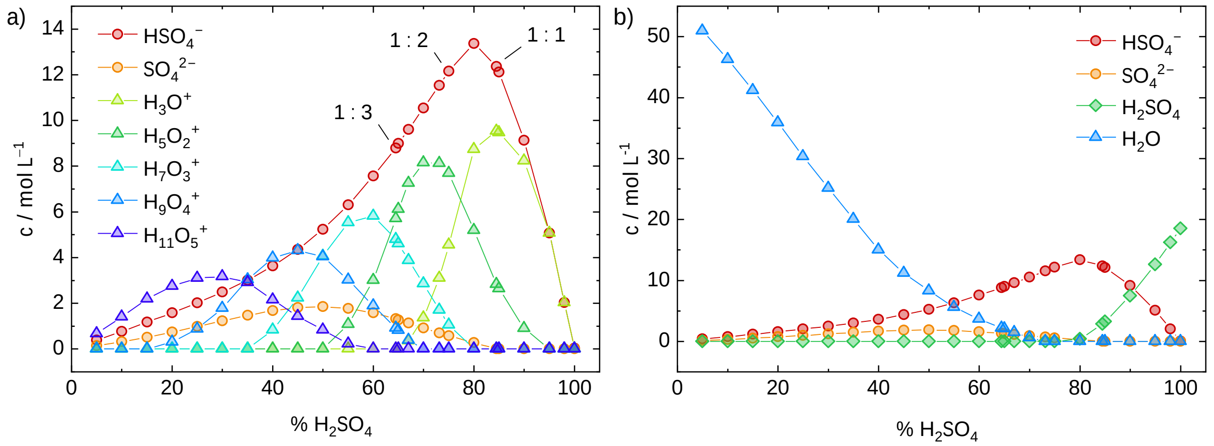

Figure S17 Concentration profile of the first mixture in mol L^{-1} against the sulfuric acid concentration in mass percent. a) Concentrations of the ions HSO_4^-, SO_4^{2-}, H_3O^+, $H_5O_2^+$ and $H_7O_3^+$, $H_9O_4^+$ and $H_{11}O_5^+$, with a highlight of the exact ratios of water to sulfuric acid of 3:1, 2:1 and 1:1 on the HSO_4^- curve. b) Concentrations of water and the main sulfuric acid species HSO_4^-, SO_4^{2-}, H_2SO_4.

The concentration of SO_4^{2-} was taken from the polynomial regression to the experimental results in Figure S15 . H_2SO_4 was assumed to dissociate completely into H_3O^+ and HSO_4^- until the maximum point determined by the regression curve. At this maximum, the dissociation is no longer complete. From here onwards, the HSO_4^- concentration approximates the regression curve, albeit not precisely, because neither the experimental nor the fitted concentrations are stoichiometrically balanced and satisfy the condition $[HSO_4^-] + [SO_4^{2-}] = c(H_2SO_4)$ exactly. It is worth noting that this concentration profile resembles the one from Robertson and Dunford,[40] who made similar assumptions but did not employ a quantum chemical method. After 73.1% (2:1) sulfuric acid, the water is distributed among the protonated water clusters, resulting in either the absence of free water or only a minimal amount remaining in the mixture. Small traces of any species do not affect the solvation energies of the other species in this theoretical environment. Once 84.5% (1:1) is reached, there is no longer free water present.

Section 3.2 summarizes the precise concentrations of all species used as input for COSMOtherm, while the calculated solvation energies can be found in Section 3.6. Taking the solvation energies of all species in this mixture, the solvation energies of the proton $\Delta_{solv}G(H^+, H_2SO_4)$ were determined for each concentration through the thermodynamic cycle depicted in Figure S12. Table S9 summarizes the thermodynamic cycles of the species E and their protonated counterparts EH^+ that most accurately represent the curve progression of the proton solvation energies observed in the ILSB and SSE experiments.

Table S9 Key thermodynamic cycles used to calculate the proton's solvation energies and absolute acidities in the aqueous sulfuric acid mixtures $\Delta_{solv}G(H^+, H_2SO_4)$, along with their designated numbers and the approximate concentration range, where these cycles apply within the first mixture provided in mass percent sulfuric acid.

EH^+	→	$H^+ + E$		Approx. concentration range %
HSO_4^-	→	$H^+ + SO_4^{2-}$	(1a)	80–90
$HSO_4^- + H_2O$	→	$H^+ + SO_4^{2-} \cdot H_2O$	(1b)	65–75
$HSO_4^- \cdot H_2O + H_2O$	→	$H^+ + SO_4^{2-} \cdot 2\ H_2O$	(1c)	5–60
$H_3SO_4^+$	→	$H^+ + H_2SO_4$	(2)	80–98
$H_5S_2O_8^+$	→	$H^+ + 2\ H_2SO_4$	(3a)	80–100
$H_5S_2O_8^+$	→	$H^+ + H_4S_2O_8$	(3b)	80–100
$H_3S_2O_8^-$	→	$H^+ + 2\ HSO_4^-$	(3c)	80–90
$H_3S_2O_8^-$	→	$H^+ + H_2S_2O_8^{2-}$	(3d)	80–90

The upcoming discussion will center on these key thermodynamic cycles identified by the numbers 1a–3d. Naturally, none of the thermodynamic cycles could accurately describe the experimental findings across the entire concentration range of aqueous sulfuric acid, thus Table S9 also provides the approximate concentration range where these cycles apply or, in simpler terms, where they fit best to the experiment. As stated during the discussion on pure sulfuric acid in Section 2.2, these cycles are not equivalent. Specifically, in the case of aqueous sulfuric acid, each concentration represents a separate system. The resulting proton solvation energies $\Delta_{solv}G(H^+)$ were used to divide the sulfuric acid solutions into different concentration regions, each represented by a different thermodynamic cycle. Figure S18 shows the proton solvation energies $\Delta_{solv}G(H^+)$ resulting from the cycles in the Table S9 split into two separate graphs to enhance readability, along with the SSE and ILSB curves.

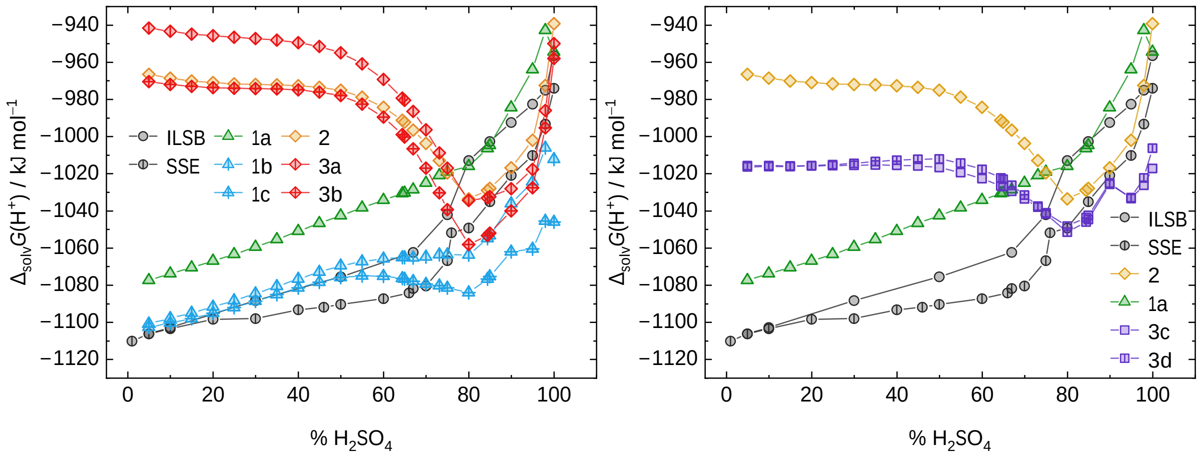

Figure S18 Gibbs energies of solvation of the proton $\Delta_{solv}G(H^+)$ determined by the thermodynamic cycles 1a–1c, 2 and 3a–3d from the solvation energies of the first approximation.

This graph illustrates how each cycle represents only portions of the mixture by reproducing certain sections of the experimental proton solvation energies. The cycles also display two main patterns based on whether a cycle uses a positively or a negatively charged EH^+. Cationic species EH^+, which are present in cycles 2, 3a, and 3b show initially lower solvation energies $\Delta_{solv}G(H^+)$ compared to the experiment and gradually increase until reaching a very prominent solvation energy maximum at 80% sulfuric acid, which is visualized as a minimum in the graph. Following that, the solvation energies decrease again, now aligning with the experimental results, until reaching 100% sulfuric acid. The thermodynamic cycles 1a–1c that contain the negatively charged HSO_4^- deliver solvation energies $\Delta_{solv}G(H^+)$ that are initially lower than the experimental energies or lie precisely on the experimental data, then decrease until reaching a concentration of 50% sulfuric acid. From there, the solvation energies $\Delta_{solv}G(H^+)$ increase until they reach their maximum at 80% H_2SO_4 that is less prominent compared to the maximum from the solvation energies of the positively charged species EH^+. When the concentration of sulfuric acid exceeds 80%, the solvation energies $\Delta_{solv}G(H^+)$ determined through any thermodynamic cycle used in these calculations decrease, which is consistent with the overall trend observed in the experimental data. The mentioned patterns are observed in the solvation energies $\Delta_{solv}G(H^+)$ of most cycles that can be constructed from the species E and EH^+ in the mixture, but there are some exceptions. Cycles 3c and 3d are such exceptions, with a trend somewhere in between the two previously described patterns. In addition to the solvation energy maximum at 80% sulfuric acid, these cycles have another minimum at 95% sulfuric acid, that is also present in cycles 1b and 1c, but in a less prominent form.

Cycles 1b and 1c are a good representation of the proton's solvation energies in the low concentrated region from 5 to approximately 60%. These cycles become less reasonable at higher concentrations since they include the water adducts of HSO_4^- and SO_4^{2-}. Protonated water clusters are more likely to bind the water, as water is a stronger base compared to HSO_4^-. Apart from that, there is a gradual decrease of the water content and the concentration of the $H_5O_2^+$ ion peaks at approximately 75% sulfuric acid, where the

proton is strongly bound by two symmetric hydrogen bonds, making it less likely that the free water is attached to the HSO_4^- instead. While SO_4^{2-} is a stronger base than water, it is present in a smaller quantity that gradually decreases beyond 50% sulfuric acid. According to Young's Raman measurements, there are no longer SO_4^{2-} ions present after a concentration of 80% H_2SO_4 is reached. The presence of SO_4^{2-} is also responsible for the sudden increase in the proton's solvation energy in the cycles 1a to 1c, from 98% to 100% sulfuric acid. However, these cycles are unsuitable for high concentrations, since the SO_4^{2-} is no longer present above 80% sulfuric acid, albeit still included at infinite dilution.

Removing the water from cycles 1c to 1b reduces the solvation energies of the species involved, which in turn decreases the solvation of the proton. Although both cycles 1b and 1c fit adequately within the diluted region, especially when compared to the ILSB measurements, cycle 1a, which is stripped of additional water, stands out due to a greater uncertainty in the diluted region. It appears that cycle 1a is a good approximation to the ILSB results in 80-90% sulfuric acid, although it does not align as well with the SSE results. These three cycles alone indicate that the potential determining species might change from the solvated HSO_4^- to a water-free version, causing the sudden decrease in the proton's solvation energy at 75% sulfuric acid. This point is very interesting, since none of the thermodynamic cycles investigated in this work follow the slope created by the experimental solvation energies, which suggests a structural change that is described by different species EH^+. Meanwhile, the ionic strength experiences a significant increase, peaking when the concentration of sulfuric acid reaches 80%. The highest concentration of HSO_4^- is reached at 80% sulfuric acid, accompanied by substantial quantities of $H_5O_2^+$ and H_3O^+. During the initial stage of these calculations, one potential explanation for the decrease in the proton's solvation energy was the transition from $H_5O_2^+$ to H_3O^+. The breaking of one of the two symmetric hydrogen bonds in the Zundel ion causes the jump to a lower solvation energy of the proton. While the calculations do not disprove this theory, as this transition might still contribute to this change, the $\Delta_{solv}G(H^+)$ values derived from the protonated water clusters do not support the protonated water clusters as potential determining species. In fact, the solvation energies $\Delta_{solv}G(H^+)$ from these cycles follow the general trend established for all positively charged species EH^+. Figure S19 illustrates the proton solvation energies resulting from the protonated water clusters $(H(H_2O)_n)^+$ present in the mixture.

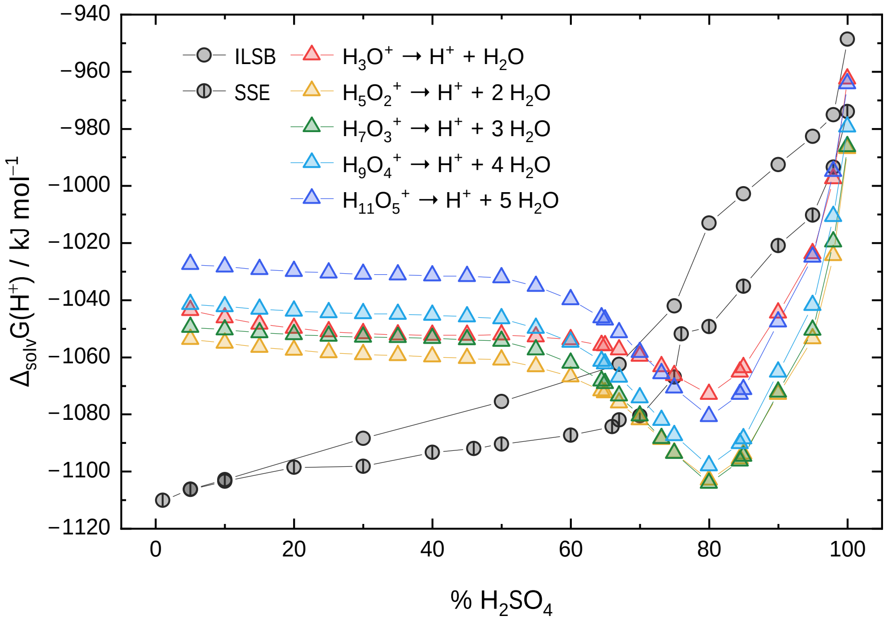

Figure S19 Gibbs energies of solvation of the proton $\Delta_{solv}G(H^+)$ obtained through thermodynamic cycles of the protonated water clusters $(H(H_2O)_n)^+$ with n = 1–5 from the solvation energies calculated with the concentrations of the first mixture.

These energies are not in agreement with the experimental results, until concentrations of approximately 98% are reached, where the solvation energy $\Delta_{solv}G(H^+)$ resulting from the H_3O^+ cycle fits in between the ILSB and SSE curves. While they do not appear to be determining the proton's chemical potential, they still

affect the solvation energies of all species present in the mixture, suggesting that they contribute to the sharp decrease in the proton's solvation energy at 75%. Even though the solvation energies of the protonated water clusters were determined for the entire concentration range, there are of course no $H_{11}O_5^+$ ions in pure sulfuric acid, just as highly diluted regions do not contain any H_3O^+ ions, although the respective solvation energies can be calculated.

One Raman study suggests a structural change at approximately 75% sulfuric acid that was attributed to the existence of *para*-sulfuric acid H_4SO_5, which dehydrates above 75% into H_2SO_4 and H_2O.[41] Theoretical studies have invalidated this interpretation by showing the extreme instability of para-sulfuric acid.[42] The presence of H_4SO_5 and $H_5SO_5^+$ in the mixture was evaluated under the condition of infinite dilution, but their calculated solvation energy did not match the experimental results at any point.

The comparison of the calculated energies with the ILSB curve suggests either a transition from cycle 1b to 1a at 75% sulfuric acid or a transition to cycles 2 and 3b. At 75% sulfuric acid cycles 1a and 2 approach the ILSB curve, with an overall high uncertainty of approximately 30 kJ mol^{-1} and an even higher uncertainty when the SSE curve is considered, where cycle 1b fits exceptionally well. The ILSB curve is well represented by the solvation energies of cycle 1a from 80 to 95% H_2SO_4, at which point cycle 2 takes over and approximates the curve up to 100% sulfuric acid. The SSE curve is better approximated by cycles 2–3b, from 80 to 100% sulfuric acid with an excellent approximation between 80 and 90% through cycle 3c and 3d. Focusing solely on the SSE curve indicates a smoother progression from 1b to 3c and 3d. When the concentration of sulfuric acid reaches 75%, water molecules become scarce in the modeled system. As a result, the amount of undissociated H_2SO_4 increases, suggesting the transition from solvated HSO_4^- to an adduct with sulfuric acid $H_3S_2O_8^-$, which is eventually replaced by $H_5S_2O_8^+$ as the concentration increases.

Unfortunately, there is a notable disparity between the two experimental methods when the sulfuric acid concentration surpasses 80%. Both methods make different assumptions, and it is unclear which method is superior, whereas both experiments carry an approximated uncertainty of 2 to 3 pH values.[34,43] When comparing the calculated solvation energies, it is important to consider the uncertainties associated with both curves to avoid drawing misleading conclusions and accurately evaluate the acidity compared to the H_0 scale. Therefore, the arithmetic mean of the results from both experimental methods was used as an additional reference. The $pH_{abs}^{H_2O}$ values of the discussed thermodynamic cycles are depicted in Figure S20 (a), with each cycle only plotted in its relevant concentration regions, along with the experimental values and their error bars of 3 pH units, expressed as fill areas. Figure S20 (b) shows the mean and a further adjustment of the concentration regions of the cycles to better align the with the mean.

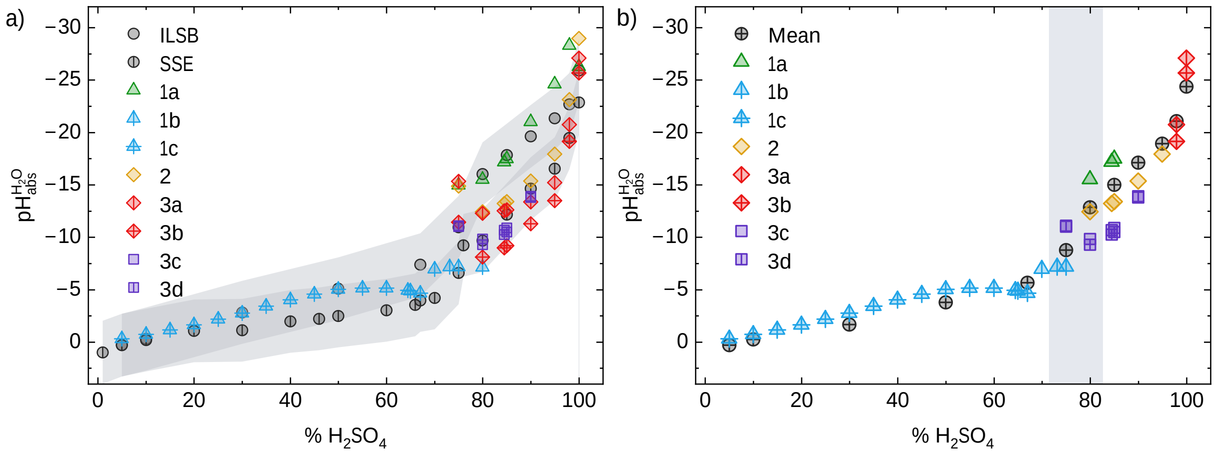

Figure S20 Absolute acidities $pH_{abs}^{H_2O}$ determined through the thermodynamic cycles 1a–1c, 1b, 2, 3a and 3b from the solvation energies of the first mixture. a) ILSB and SSE measurements and their respective error bars. b) Arithmetic mean of the ILSB and SSE curves with a highlight of the area where both experiments show a sudden decrease in the $pH_{abs}^{H_2O}$ values.

Most of the calculated values shown in Figure S20 (a) fall comfortably within the error bars of both experiments. However, the ideal thermodynamic cycles that reflect either the ILSB or the SSE curve change when the mean is considered instead. Comparing the results of the cycles to the mean does not change the previous discussion of the cycles 1b and 1c, but the concentrated region above 75% sulfuric acid is more aligned with the interpretation that also fits to the SSE results. The $pH_{abs}^{H_2O}$ values determined through cycle 1a show a greater deviation from the mean compared to the ILSB results. Between approximately 80 and 90% sulfuric acid, cycles 2, 3c and 3d are closest to the mean. Above 95%, cycles 3a and 3b remain the best approximation for all experimental values, since the discrepancy of the ILSB and SSE values decreases at this point. This supports the previously stated transition of the dominant species at 75% sulfuric acid from solvated HSO_4^- to the adduct $H_3S_2O_8^-$. This is followed by a transition to $H_3SO_4^+$ and its corresponding adduct with sulfuric acid, $H_5S_2O_8^+$, which becomes more significant as the concentration increases.

The concentration profile used for these calculations is an approximation, which introduces uncertainties related to the assumptions made, the Raman data used as a reference, and the theoretical methods employed to calculate the solvation energies. Young's Raman measurements revealed that the 1:1 mixture of sulfuric acid and water does not result in the formation of an ionic liquid containing H_3O^+ and HSO_4^- because of the incomplete dissociation of sulfuric acid. To address the uncertainty in the concentrations, additional modifications were made to the mixture in order to examine the potential effects of a smaller dissociation of H_2SO_4.

2.3.2 Adjusting the Concentrations of the First Approximation

The dissociation of H_2SO_4 to HSO_4^- and SO_4^{2-} was reduced below the experimentally determined values to create two additional mixtures. To achieve this, the concentrations of both HSO_4^- and SO_4^{2-} were lowered and the concentrations of the remaining components were adjusted accordingly with the same approach as outlined in Section 2.3.1 for the first mixture. These modifications were primarily made to concentrations of 64–98% sulfuric acid, with minimal changes to the diluted part. The concentrations of HSO_4^- and SO_4^{2-} were reduced by the same percentage at each concentration, resulting in minimal changes in the amount of SO_4^{2-} due to its already low concentration. Figure S21 combines the concentration profiles of the second (c) and third approximation (d) at the bottom and a comparison of the HSO_4^-, SO_4^{2-} and H_2SO_4 concentrations of the three mixtures at the top.

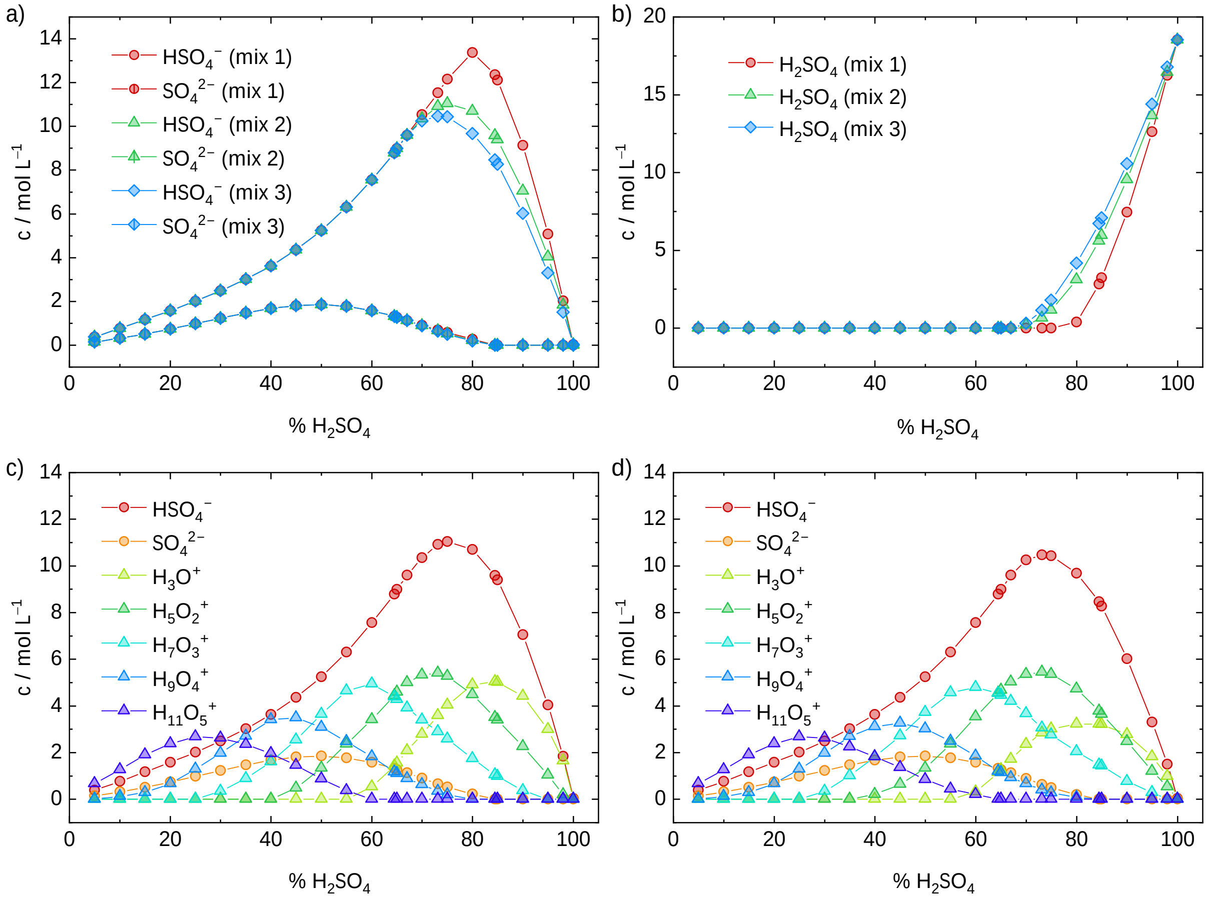

Figure S21 Modifications of the concentration profile of the first mixture. a) Change in the concentrations of HSO_4^- and SO_4^{2-} from the first to the third mixture. b) Change in the concentrations of undissociated H_2SO_4 from the first to the third mixture. c) Concentration profile of the second mixture. d) Concentration profile of the third mixture.

Decreasing the concentration of HSO_4^- naturally reduces the amount of H_3O^+, resulting in a shift towards larger protonated water clusters, since the amount of water remains constant while the number of negatively charged ions decreases. The adjustments ensured that concentration curves for all species remained smooth, without any sudden changes. The width of the concentration ranges of the water clusters increased slightly, with less distinct peaks, which shifted marginally towards lower concentrations of sulfuric acid. The same holds true for the position of the concentration maximum of HSO_4^- (Figure S21a), but not the maximum SO_4^{2-} concentration, as it is situated in the diluted region that was not changed. The concentration of HSO_4^- is noticeably lower in the second mix, while the change in the third mix is minimal in comparison. No additional modifications were made to avoid straying too far from the experimentally determined concentrations. Figure S22 shows an overview of how these changes affect the $pH_{abs}^{H_2O}$ values resulting from cycles 2 and 3b on the left and cycle 1a on the right side (Table S9).

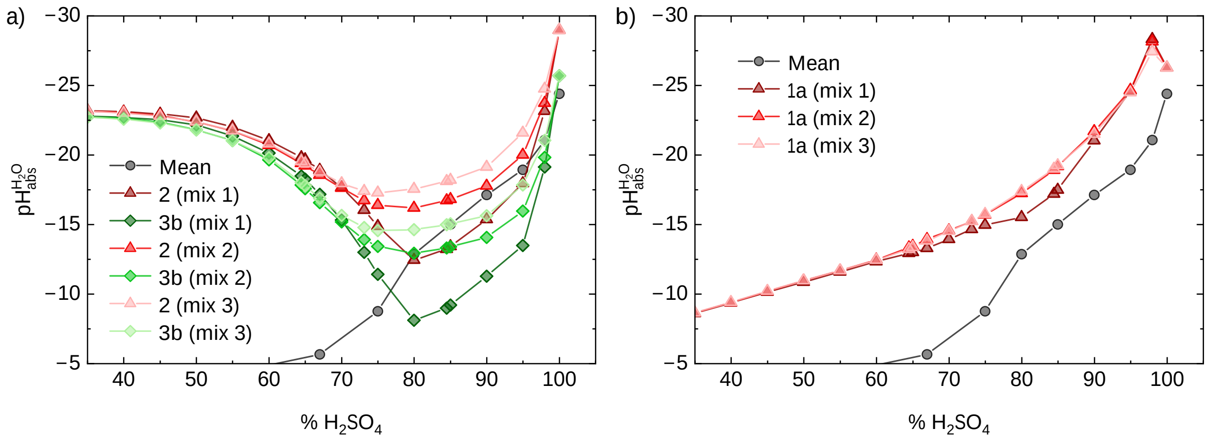

Figure S22 Calculated $pH_{abs}^{H_2O}$ values from all three mixtures, together with the mean of the two experimental procedures. a) Results from cycles 2 and 2b. b) Results from the 1a cycle.

The absolute acidities $pH_{abs}^{H_2O}$ consistently decrease as the concentration of undissociated sulfuric acid increases, as illustrated by both graphs. The change is more pronounced in all cycles with positively charged EH^+ species, as demonstrated by cycles 2 and 3b, compared to cycle 1a containing HSO_4^- and SO_4^{2-}. A slight change in the concentrations between the second and third mixtures leads to a noticeable increase in the $pH_{abs}^{H_2O}$ derived from cycles 2, 2a and 3b. Cycles 1a to 1c are overall less sensitive to these changes in concentration. The variations in the $pH_{abs}^{H_2O}$ values due to the change in concentration remain mostly consistent across cycles, regardless of the specific species EH^+ involved, as long as it maintains the same charge.

Figure S23 shows the absolute acidities $pH_{abs}^{H_2O}$ determined through the same thermodynamic cycles summarized in Table S9, with the second mixture on the left side and the third mixture on the right side. The top section of the diagram displays the ILSB and SSE curves along with their corresponding error bars, while the bottom section presents the mean and only the experimental data that is nearest to the mean.

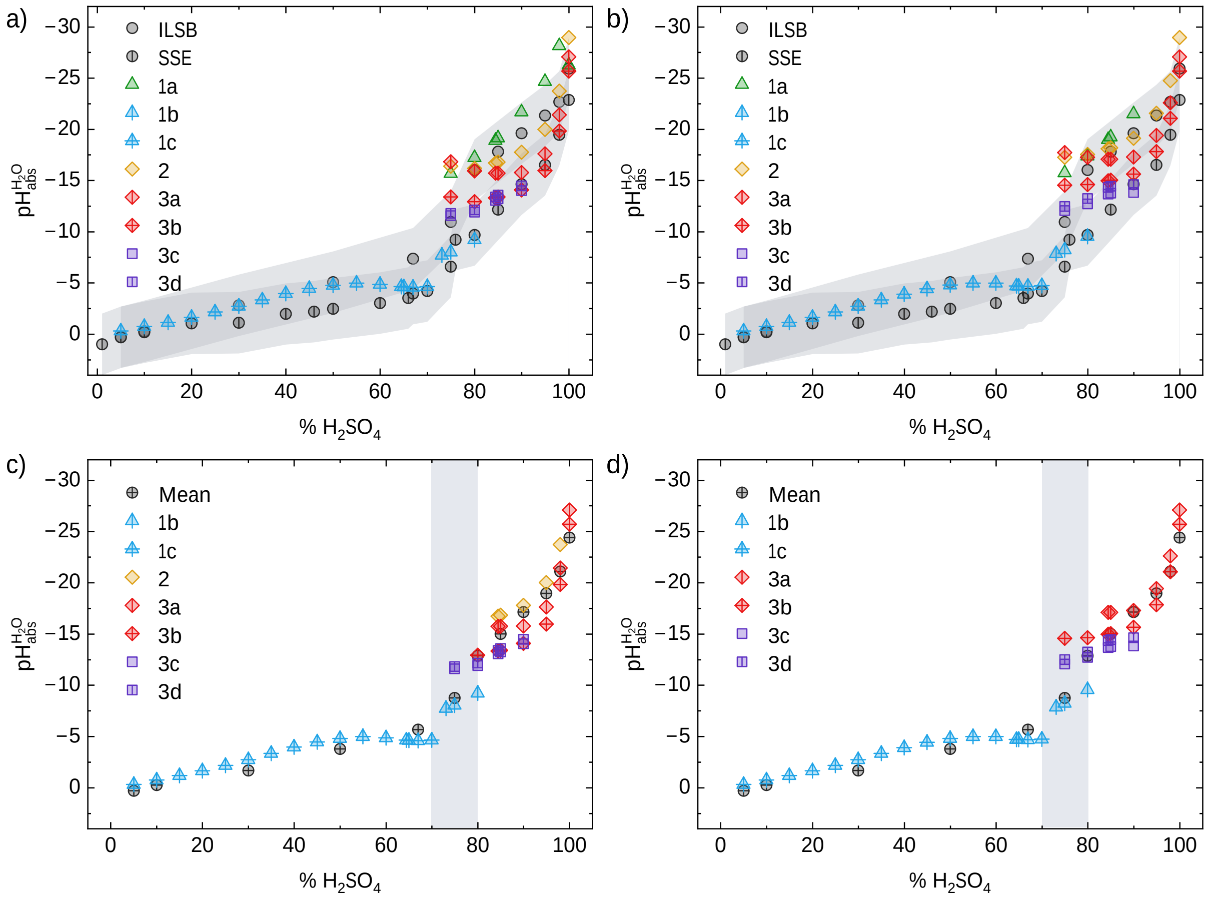

Figure S23 Calculated $pH_{abs}^{H_2O}$ values from the second (a) and the third mixture (b) of the concentrations, along with the SSE and ILSB measurements with its error bars. The two graphs at the bottom contain the calculated $pH_{abs}^{H_2O}$ of the second (c) and the third mixture (d) that are closest to the mean of the two experimental methods.

Apart from the upward shift on the y-axis, the calculated $pH_{abs}^{H_2O}$ values remain within the error bars of both experiments. Therefore, even if there were significant uncertainties in the Raman data used to determine the concentrations of H_2SO_4, HSO_4^-, and SO_4^{2-}, the results would remain consistent. The same principle can be applied to the concentrations of protonated water clusters, which have not been determined through experimental methods but primarily derived from the dissociation equilibrium of sulfuric acid. The ILSB curve is shifted towards higher $pH_{abs}^{H_2O}$ compared to the SSE curve, indicating that the second and especially the third approximation provide a more accurate description of the ILSB results.

The approximation of the slope at 75% through cycle 1b does slightly improve in the second and the third mixture, suggesting that the sudden change in the $pH_{abs}^{H_2O}$ values may be partly due to an increasing concentration of HSO_4^- with decreasing availability of free water for its solvation. This coincides with the change of the potential determining species that now seems to be a little shifted towards approximately 80% rather than the 75% that the ILSB curve suggests. However, since only one measurement was taken at 75% sulfuric acid using the ILSB method, the uncertainty at this specific point is higher compared to the other concentrations where multiple measurements were conducted. [34] Moreover, the increase in the chemical potential of the proton, as measured by the SSE method, is not as pronounced in comparison. Thus, the precise location of this abrupt increase in acidity in the experimental data, although clearly present, is associated with a greater uncertainty. [34] The comparison of the $pH_{abs}^{H_2O}$ values from various cycles to the mean reinforces the previous interpretation, which is most prevalent in the third mixture. The calculations suggest that a transition from cycles 1b and 1c to cycles 3c and 3d occurs when the sulfuric acid concentration reaches 75% and an additional structural change represented by a shift to cycles 3a and 3b when the sulfuric acid concentration reaches 90%.

Returning to the discussion of pure sulfuric acid in Section 2.2 and taking the whole curve progression leading into the pure sulfuric acid into account, it appears that the two cycles with the $H_5S_2O_8^+$ (3a and 3b) are the best representation of this region. The thermodynamic cycles listed in Table Table S6 exhibit considerable variation in their predicted $pH_{abs}^{H_2O}$ values, but once the values from the aqueous mixtures of sulfuric acid are included, a clear pattern emerges. As mentioned earlier, this agrees with the high viscosity of pure sulfuric acid, but it also agrees with its progression as a function of the sulfuric acid concentration. The viscosity of sulfuric acid does not increase steadily with its concentration. Instead, a maximum is observed at 83–85%, followed by a decrease to a minimum viscosity in the range of 92–94% sulfuric acid.[33] Afterwards, the viscosity continues to increase until it reaches its highest point at 100% sulfuric acid. These extremes are often attributed to the formation of a sulfuric acid monohydrate at the viscosity maximum, based on stoichiometric considerations, and a eutectic mixture at the viscosity minimum.[44] Figure S24 illustrates the relationship between the calculated absolute acidities $pH_{abs}^{H_2O}$ from the concentrations of the third mixture, the viscosities and the conductivities as a function of sulfuric acid concentration. The kinematic viscosities at 25°C were obtained from Rhodes and Barbour[33] and the conductivities at 26.7°C were taken from Darling.[45] The gas-phase structures of the four potential determining species are depicted at the DSD-PBEP86/def2-TZVPP level of theory. These calculations suggest that the highest viscosity occurs when HSO_4^- is no longer attached to free water and forms hydrogen bonds to the protonated water clusters and other sulfuric acid species, as the H_2SO_4 concentrations increases. Following the sudden decrease of the proton's chemical potential at 75%, the viscosity reaches its maximum, which coincides with a local minimum in conductivity. The viscosity maximum is slightly past the point where Young's Raman measurements show a maximum concentration of hydrogensulfate at 79.4% sulfuric acid, which is the concentration that was used for the first mixture. This HSO_4^- concentration maximum was shifted to 73% to create the third mixture, which corresponds to the stoichiometric composition $(H_2O)_2(H_2SO_4)$.

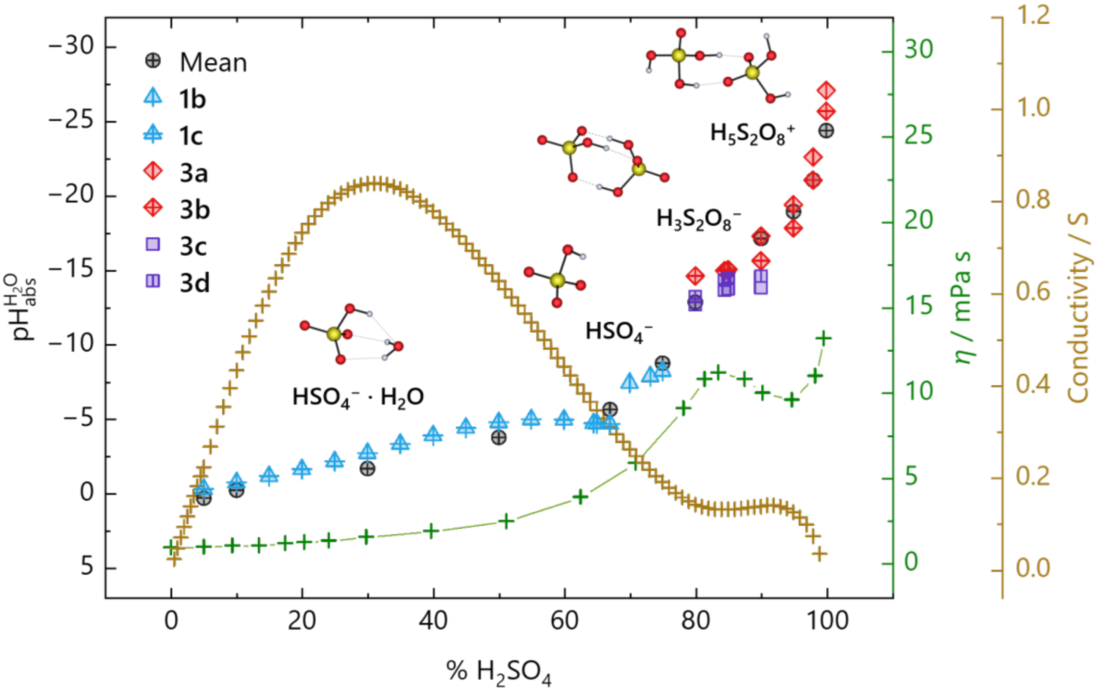

Figure S24 Calculated $pH_{abs}^{H_2O}$ values from the concentrations of the third mixture, along with the mean of the two experimental methods, the kinematic viscosity and the conductivity plotted against the sulfuric acid concentration in mass percent. The potential determining species as defined by the respective thermodynamic cycles is highlighted at each point, including its gas-phase structure at the DSD-PBEP86/def2-TZVPP level of theory. The kinematic viscosities in mPa s at 25°C was taken from Rhodes and Barbour[33] and the conductivity in Siemens at 26.7°C was taken from Darling.[45]

HSO_4^- can form three hydrogen bonds by accepting protons to three of its oxygen atoms and donating a hydrogen atom to the oxygen of another species in the mixture. This creates a strong hydrogen bond network with the $H_5O_2^+$ and the H_3O^+ ions, possibly causing the observed viscosity maximum. In their extensive work comparing the extrema in the conductivity, density, and viscosity data of sulfuric acid, Walrafen *et al*. suggest that the maximum viscosity can be attributed to the $(HSO_4^-)(H_3O^+)$ ion pair,[46] but concluded in a later Raman study concentrations of 8.2 mol L^{-1} and 5.4 mol L^{-1} for H_3O^+ and $H_5O_2^+$, respectively.[47] The former study also suggested that the viscosity minimum, which is also the conductivity maximum, occurs due to the presence of the hydrogen bonded species $(H_2SO_4)_2(H_3O^+)(HSO_4^-)$ derived from stoichiometric considerations. In this area, the calculations indicate that the potential is determined by the hydrogensulfate adduct with sulfuric acid, $H_3S_2O_8^-$. Therefore, the main network is defined by $(H_2SO_4)_k(H_3O^+)_n(H_3S_2O_8^-)_m$ with increasing k and decreasing m from the first to the third mixture and small amounts of $H_5O_2^+$. Once the viscosity reaches its maximum, the concentration of the $H_3S_2O_8^-$ adduct increases, lowering the viscosity and leading to the minimum at 83% sulfuric acid. Although certainly bound by intermolecular hydrogen bonds, $H_3S_2O_8^-$ itself does not have free hydrogen atoms, leading to less bonding opportunities. More importantly, it has a relatively compact size in a framework where the intermolecular bonding situation is overall very similar. Furthermore, the $H_5O_2^+$ ion is slowly replaced by the H_3O^+ ion, which is both smaller and more mobile, contributing to the viscosity minimum and conductivity maximum. Near 90% sulfuric acid, the potential determining species transitions to $H_5S_2O_8^+$, which increases the viscosity once again. Besides the simultaneous decrease in the H_3O^+ concentration, which leads to increased viscosity and reduced conductivity, $H_5S_2O_8^+$ also contains additional free hydrogen atoms that are not present in the $H_3S_2O_8^-$ ion. This leads to a larger size of $H_5S_2O_8^+$, which has additional free hydrogen bonds to contribute to the network. The viscosity of a system cannot be accurately determined solely through consideration of hydrogen bonds, as multiple factors can impact the viscosity, but it allows for some rough estimates.

Both the $H_3SO_4^+$ and specifically the $H_5S_2O_8^+$ cycles match the experiment from 80 to 100% sulfuric acid. The $H_3SO_4^+$ cycle matches the mean of the experiments in the initial mixture, whereas the cycle of its sulfuric acid adduct provides a more accurate representation of the mean in the second and third approximations. There is no experimental data supporting the presence of $H_3SO_4^+$ or $H_5S_2O_8^+$ at any point, besides the pure sulfuric acid through its autoprotolysis. Nevertheless, even negligible amounts are enough to determine the potential, since the species that determines the proton's chemical potential is not necessarily linked to the most abundant species in the mixture. However, it is important to note that this is a purely theoretical and conceptual model that uses specific compounds to approximate the absolute acidities $pH_{abs}^{H_2O}$ through different thermodynamic cycles. Thus, the $H_5S_2O_8^+$ ion could determine the potential of the proton even if its concentration is negligible. Its existence before the viscosity minimum or in its vicinity could additionally explain the conductivity maximum, as the $H_5S_2O_8^+$ ion allows for a Grotthuss-type mechanism, while $H_3S_2O_8^-$ lowers the viscosity. The calculations presented here provide a simplified and approximate understanding of sulfuric acid, so there may be additional chemical components and larger adducts of the species investigated in this study. Nevertheless, these simple compounds allow for the application of thermodynamic cycles to determine the absolute acidities. These calculations are in agreement with the experimental results[34] and show that the H_0 scale underestimates the acidity of not only pure sulfuric acid but also the concentrated region above 70% sulfuric acid.

The $pH_{abs}^{H_2O}$ values in aqueous sulfuric acid cannot be determined through the indicator bases used for pure sulfuric acid in Section 2.2.1. Currently, the $pK_{a,H_2O}(BH^+, aq.\,H_2SO_4)$ values for each base at every concentration, which are necessary to extend this approach to the aqueous mixtures, are not available. It is therefore necessary to identify the species that can determine the chemical potential of the proton, which makes this method more complicated as it requires a fundamental understanding of the composition.

3 Computation Results

Raw data from the calculations are summarized here, including frequencies, thermochemical data, and structure files in ASCII format with Cartesian coordinates, are available at 10.5281/zenodo.14536737.

3.1 Polynomial Regression to the Raman Data

Table S10 Details of the polynomial regression analysis conducted on the experimental concentrations of HSO_4^- and SO_4^{2-} from Young et. *al.*[39] that are visualized in Figure S15 .

Parameters	$c(SO_4^{2-})$ mol L^{-1}	$c(HSO_4^-)$ mol L^{-1}
Equation	$y = m + B_1x + B_2x^2 + B_3x^3 + B_4x^4 + B_5x^5 + B_6x^6 + B_7x^7 + B_8x^8$	
m	0	$-5.6318177 \cdot 10^{-2}$
B_1	$-5.0136669 \cdot 10^{-6}$	$-3.9625261 \cdot 10^{-8}$
B_2	$2.0187653 \cdot 10^{-4}$	$5.9070934 \cdot 10^{-6}$
B_3	$-2.5273436 \cdot 10^{-3}$	$-2.5199464 \cdot 10^{-4}$
B_4	$7.1487496 \cdot 10^{-3}$	$4.7668073 \cdot 10^{-3}$
B_5	$1.6416849 \cdot 10^{-2}$	$-4.5443552 \cdot 10^{-2}$
B_6	$2.6579967 \cdot 10^{-1}$	$2.2428126 \cdot 10^{-1}$
B_7	0	$-5.2335404 \cdot 10^{-1}$
B_8	0	1.1268884
R^2	0.99994	0.99917

3.2 Concentrations

The mole fractions with 4 significant figures that are provided in this section were used as input for the COSMOtherm calculations.

Table S11 Concentrations of the first mixture used to calculate the solvation energies of all species with COSMOtherm expressed in mol L^{-1} for each concentration of sulfuric acid in mass percent %. Species not listed were included at infinite dilution.

H_2SO_4 %	HSO_4^-	SO_4^{2-}	$H_3SO_4^+$	$HS_2O_7^-$	H_3O^+	$H_5O_2^+$	$H_7O_3^+$	$H_9O_4^+$	$H_{11}O_5^+$	H_2SO_4	H_2O
5	0.380	0.145	0.000	0.000	0.000	0.000	0.000	0.000	0.670	0.000	50.966
10	0.771	0.314	0.000	0.000	0.000	0.000	0.000	0.000	1.398	0.000	46.164
15	1.172	0.510	0.000	0.000	0.000	0.000	0.000	0.000	2.191	0.000	40.917
20	1.585	0.733	0.000	0.000	0.000	0.000	0.000	0.305	2.745	0.000	35.522
25	2.018	0.977	0.000	0.000	0.000	0.000	0.000	0.874	3.098	0.000	29.931
30	2.487	1.229	0.000	0.000	0.000	0.000	0.000	1.780	3.165	0.000	24.263
35	3.013	1.470	0.000	0.000	0.000	0.000	0.000	3.036	2.917	0.000	18.600
40	3.625	1.673	0.000	0.000	0.000	0.000	0.837	3.987	2.147	0.000	14.073
45	4.356	1.809	0.000	0.000	0.000	0.000	2.233	4.306	1.435	0.000	9.923
50	5.241	1.851	0.000	0.000	0.000	0.000	4.069	4.045	0.829	0.000	6.078
55	6.307	1.775	0.000	0.000	0.000	1.084	5.527	3.019	0.227	0.000	4.040
60	7.564	1.575	0.000	0.000	0.000	3.000	5.825	1.890	0.000	0.000	2.137
64[a]	8.791	1.318	0.000	0.000	0.000	5.713	4.799	0.914	0.000	0.000	0.846
65	8.994	1.272	0.000	0.000	0.000	6.115	4.609	0.813	0.000	0.000	0.783
67	9.603	1.132	0.000	0.000	0.356	7.252	3.876	0.383	0.000	0.000	0.765
70	10.546	0.916	0.000	0.000	1.361	8.152	2.864	0.000	0.000	0.000	0.486
73[a]	11.537	0.698	0.000	0.000	3.104	8.124	1.705	0.000	0.000	0.000	0.002
75	12.155	0.573	0.000	0.000	4.553	7.698	1.050	0.000	0.000	0.000	0.000
80	13.373	0.278	0.000	0.000	8.742	5.187	0.000	0.000	0.000	0.395	0.002
84[a]	12.368	0.000	0.000	0.000	9.542	2.826	0.000	0.000	0.000	2.826	0.000
85	12.122	0.000	0.000	0.000	9.479	2.643	0.000	0.000	0.000	3.246	0.000
90	9.138	0.000	0.000	0.000	8.235	0.904	0.000	0.000	0.000	7.462	0.000
95	5.075	0.000	0.000	0.000	5.075	0.000	0.000	0.000	0.000	12.637	0.000
98	2.033	0.000	0.000	0.000	2.033	0.000	0.000	0.000	0.000	16.263	0.000
100	0.028	0.000	0.028	0.012	0.012	0.000	0.000	0.000	0.000	18.533	0.000

[a] Precise ratios of water to sulfuric acid are 3:1 (64.47), 2:1 (73.13), and 1:1 (84.48)

Table S12 Concentrations of the first mixture used to calculate the solvation energies of all species with COSMOtherm expressed in mole fractions for each concentration of sulfuric acid in mass percent %. Species not listed were included at infinite dilution.

H_2SO_4 %	HSO_4^-	SO_4^{2-}	$H_3SO_4^+$	$HS_2O_7^-$	H_3O^+	$H_5O_2^+$	$H_7O_3^+$	$H_9O_4^+$	$H_{11}O_5^+$	H_2SO_4	H_2O
5	0.0073	0.0028	0.0000	0.0000	0.0000	0.0000	0.0000	0.0000	0.0128	0.0000	0.9771
10	0.0159	0.0064	0.0000	0.0000	0.0000	0.0000	0.0000	0.0000	0.0287	0.0000	0.9490
15	0.0262	0.0114	0.0000	0.0000	0.0000	0.0000	0.0000	0.0000	0.0489	0.0000	0.9135
20	0.0388	0.0179	0.0000	0.0000	0.0000	0.0000	0.0000	0.0075	0.0671	0.0000	0.8687
25	0.0547	0.0265	0.0000	0.0000	0.0000	0.0000	0.0000	0.0237	0.0840	0.0000	0.8112
30	0.0755	0.0373	0.0000	0.0000	0.0000	0.0000	0.0000	0.0541	0.0961	0.0000	0.7369
35	0.1038	0.0506	0.0000	0.0000	0.0000	0.0000	0.0000	0.1046	0.1005	0.0000	0.6406
40	0.1376	0.0635	0.0000	0.0000	0.0000	0.0000	0.0318	0.1514	0.0815	0.0000	0.5342
45	0.1810	0.0752	0.0000	0.0000	0.0000	0.0000	0.0928	0.1790	0.0597	0.0000	0.4124
50	0.2370	0.0837	0.0000	0.0000	0.0000	0.0000	0.1840	0.1829	0.0375	0.0000	0.2749
55	0.2869	0.0808	0.0000	0.0000	0.0000	0.0493	0.2515	0.1373	0.0103	0.0000	0.1838
60	0.3439	0.0716	0.0000	0.0000	0.0000	0.1364	0.2649	0.0859	0.0000	0.0000	0.0972
64	0.3928	0.0589	0.0000	0.0000	0.0000	0.2553	0.2144	0.0408	0.0000	0.0000	0.0378
65	0.3982	0.0563	0.0000	0.0000	0.0000	0.2707	0.2041	0.0360	0.0000	0.0000	0.0347
67	0.4109	0.0485	0.0000	0.0000	0.0152	0.3104	0.1659	0.0164	0.0000	0.0000	0.0327
70	0.4335	0.0376	0.0000	0.0000	0.0560	0.3351	0.1177	0.0000	0.0000	0.0000	0.0200
73	0.4583	0.0277	0.0000	0.0000	0.1233	0.3228	0.0678	0.0000	0.0000	0.0000	0.0001
75	0.4670	0.0220	0.0000	0.0000	0.1749	0.2958	0.0403	0.0000	0.0000	0.0000	0.0000
80	0.4780	0.0099	0.0000	0.0000	0.3125	0.1854	0.0000	0.0000	0.0000	0.0141	0.0001
84	0.4487	0.0000	0.0000	0.0000	0.3462	0.1025	0.0000	0.0000	0.0000	0.1025	0.0000
85	0.4410	0.0000	0.0000	0.0000	0.3448	0.0961	0.0000	0.0000	0.0000	0.1181	0.0000
90	0.3550	0.0000	0.0000	0.0000	0.3199	0.0351	0.0000	0.0000	0.0000	0.2899	0.0000
95	0.2227	0.0000	0.0000	0.0000	0.2227	0.0000	0.0000	0.0000	0.0000	0.5546	0.0000
98	0.1000	0.0000	0.0000	0.0000	0.1000	0.0000	0.0000	0.0000	0.0000	0.8000	0.0000
100	0.0015	0.0000	0.0015	0.0006	0.0006	0.0000	0.0000	0.0000	0.0000	0.9957	0.0000

Table S13 Concentrations of the second mixture used to calculate the solvation energies of all species with COSMOtherm expressed in mol L^{-1} for each concentration of sulfuric acid in mass percent %. Species not listed were included at infinite dilution.

H_2SO_4 %	HSO_4^-	SO_4^{2-}	$H_3SO_4^+$	$HS_2O_7^-$	H_3O^+	$H_5O_2^+$	$H_7O_3^+$	$H_9O_4^+$	$H_{11}O_5^+$	H_2SO_4	H_2O
5	0.380	0.145	0.000	0.000	0.000	0.000	0.000	0.000	0.670	0.000	50.966
10	0.771	0.314	0.000	0.000	0.000	0.000	0.000	0.126	1.273	0.000	46.289
15	1.172	0.510	0.000	0.000	0.000	0.000	0.000	0.285	1.906	0.000	41.202
20	1.585	0.733	0.000	0.000	0.000	0.000	0.000	0.671	2.379	0.000	35.888
25	2.018	0.977	0.000	0.000	0.000	0.000	0.000	1.291	2.681	0.000	30.348
30	2.487	1.229	0.000	0.000	0.000	0.000	0.346	1.978	2.622	0.000	25.152
35	3.013	1.470	0.000	0.000	0.000	0.000	0.893	2.707	2.353	0.000	20.057
40	3.625	1.673	0.000	0.000	0.000	0.000	1.603	3.408	1.959	0.000	15.028
45	4.356	1.809	0.000	0.000	0.000	0.478	2.549	3.488	1.459	0.000	11.172
50	5.241	1.851	0.000	0.000	0.000	1.341	3.649	3.083	0.870	0.000	8.300
55	6.307	1.775	0.000	0.000	0.000	2.366	4.645	2.477	0.370	0.000	5.577
60	7.564	1.575	0.000	0.000	0.536	3.410	4.942	1.828	0.000	0.000	3.681
64	8.791	1.318	0.000	0.000	1.428	4.449	4.384	1.165	0.000	0.000	2.187
65	8.994	1.272	0.000	0.000	1.558	4.591	4.284	1.105	0.000	0.000	2.082
67	9.603	1.132	0.000	0.000	2.077	4.993	3.910	0.888	0.000	0.000	1.444
70	10.353	0.897	0.000	0.000	2.794	5.332	3.399	0.623	0.000	0.211	0.596
73	10.915	0.658	0.000	0.000	3.584	5.405	2.902	0.341	0.000	0.662	0.008
75	11.045	0.518	0.000	0.000	4.035	5.270	2.581	0.194	0.000	1.165	0.001
80	10.704	0.215	0.000	0.000	4.899	4.489	1.746	0.000	0.000	3.127	0.003
84	9.589	0.000	0.000	0.000	5.034	3.507	1.048	0.000	0.000	5.605	0.003
85	9.400	0.000	0.000	0.000	5.020	3.396	0.984	0.000	0.000	5.967	0.000
90	7.046	0.000	0.000	0.000	4.419	2.259	0.368	0.000	0.000	9.555	0.000
95	4.035	0.000	0.000	0.000	2.994	1.040	0.000	0.000	0.000	13.677	0.000
98	1.840	0.000	0.000	0.000	1.647	0.193	0.000	0.000	0.000	16.456	0.000
100	0.028	0.000	0.028	0.012	0.012	0.000	0.000	0.000	0.000	18.533	0.000

Table S14 Concentrations of the second mixture used to calculate the solvation energies of all species with COSMOtherm expressed in mole fractions for each concentration of sulfuric acid in mass percent %. Species not listed were included at infinite dilution.

H_2SO_4 %	HSO_4^-	SO_4^{2-}	$H_3SO_4^+$	$HS_2O_7^-$	H_3O^+	$H_5O_2^+$	$H_7O_3^+$	$H_9O_4^+$	$H_{11}O_5^+$	H_2SO_4	H_2O
5	0.0073	0.0028	0.0000	0.0000	0.0000	0.0000	0.0000	0.0000	0.0128	0.0000	0.9771
10	0.0158	0.0064	0.0000	0.0000	0.0000	0.0000	0.0000	0.0026	0.0261	0.0000	0.9491
15	0.0260	0.0113	0.0000	0.0000	0.0000	0.0000	0.0000	0.0063	0.0423	0.0000	0.9141
20	0.0384	0.0178	0.0000	0.0000	0.0000	0.0000	0.0000	0.0163	0.0577	0.0000	0.8699
25	0.0541	0.0262	0.0000	0.0000	0.0000	0.0000	0.0000	0.0346	0.0719	0.0000	0.8133
30	0.0736	0.0364	0.0000	0.0000	0.0000	0.0000	0.0102	0.0585	0.0775	0.0000	0.7438
35	0.0988	0.0482	0.0000	0.0000	0.0000	0.0000	0.0293	0.0888	0.0772	0.0000	0.6578
40	0.1328	0.0613	0.0000	0.0000	0.0000	0.0000	0.0587	0.1249	0.0718	0.0000	0.5505
45	0.1721	0.0715	0.0000	0.0000	0.0000	0.0189	0.1007	0.1378	0.0577	0.0000	0.4414
50	0.2154	0.0761	0.0000	0.0000	0.0000	0.0551	0.1499	0.1267	0.0357	0.0000	0.3411
55	0.2682	0.0755	0.0000	0.0000	0.0000	0.1006	0.1975	0.1053	0.0157	0.0000	0.2372
60	0.3214	0.0669	0.0000	0.0000	0.0228	0.1449	0.2100	0.0777	0.0000	0.0000	0.1564
64	0.3706	0.0555	0.0000	0.0000	0.0602	0.1876	0.1848	0.0491	0.0000	0.0000	0.0922
65	0.3765	0.0533	0.0000	0.0000	0.0652	0.1922	0.1794	0.0463	0.0000	0.0000	0.0872
67	0.3994	0.0471	0.0000	0.0000	0.0864	0.2077	0.1626	0.0369	0.0000	0.0000	0.0600
70	0.4277	0.0371	0.0000	0.0000	0.1154	0.2203	0.1404	0.0258	0.0000	0.0087	0.0246
73	0.4460	0.0269	0.0000	0.0000	0.1464	0.2208	0.1186	0.0139	0.0000	0.0271	0.0003
75	0.4452	0.0209	0.0000	0.0000	0.1626	0.2124	0.1041	0.0078	0.0000	0.0469	0.0000
80	0.4250	0.0085	0.0000	0.0000	0.1945	0.1783	0.0693	0.0000	0.0000	0.1242	0.0001
84	0.3869	0.0000	0.0000	0.0000	0.2031	0.1415	0.0423	0.0000	0.0000	0.2261	0.0001
85	0.3795	0.0000	0.0000	0.0000	0.2027	0.1371	0.0397	0.0000	0.0000	0.2409	0.0000
90	0.2980	0.0000	0.0000	0.0000	0.1869	0.0956	0.0156	0.0000	0.0000	0.4040	0.0000
95	0.1855	0.0000	0.0000	0.0000	0.1377	0.0478	0.0000	0.0000	0.0000	0.6289	0.0000
98	0.0914	0.0000	0.0000	0.0000	0.0818	0.0096	0.0000	0.0000	0.0000	0.8173	0.0000
100	0.0015	0.0000	0.0015	0.0006	0.0006	0.0000	0.0000	0.0000	0.0000	0.9957	0.0000

Table S15 Concentrations of the third mixture used to calculate the solvation energies of all species with COSMOtherm expressed in mol L^{-1} for each concentration of sulfuric acid in mass percent %. Species not listed were included at infinite dilution.

H_2SO_4 %	HSO_4^-	SO_4^{2-}	$H_3SO_4^+$	$HS_2O_7^-$	H_3O^+	$H_5O_2^+$	$H_7O_3^+$	$H_9O_4^+$	$H_{11}O_5^+$	H_2SO_4	H_2O
5	0.380	0.145	0.000	0.000	0.000	0.000	0.000	0.000	0.670	0.000	50.966
10	0.771	0.314	0.000	0.000	0.000	0.000	0.000	0.126	1.273	0.000	46.289
15	1.172	0.510	0.000	0.000	0.000	0.000	0.000	0.285	1.906	0.000	41.202
20	1.585	0.733	0.000	0.000	0.000	0.000	0.000	0.671	2.379	0.000	35.888
25	2.018	0.977	0.000	0.000	0.000	0.000	0.000	1.291	2.681	0.000	30.348
30	2.487	1.229	0.000	0.000	0.000	0.000	0.346	1.978	2.622	0.000	25.152
35	3.013	1.470	0.000	0.000	0.000	0.000	1.012	2.693	2.248	0.000	20.281
40	3.625	1.673	0.000	0.000	0.000	0.209	1.826	3.110	1.826	0.000	15.801
45	4.356	1.809	0.000	0.000	0.000	0.638	2.715	3.259	1.363	0.000	11.752
50	5.241	1.851	0.000	0.000	0.000	1.341	3.725	3.024	0.853	0.000	8.393
55	6.307	1.775	0.000	0.000	0.000	2.366	4.570	2.483	0.438	0.000	5.434
60	7.564	1.575	0.000	0.000	0.321	3.534	4.802	1.852	0.206	0.000	2.940
64	8.791	1.318	0.000	0.000	1.143	4.546	4.533	1.205	0.000	0.000	1.673
65	8.994	1.272	0.000	0.000	1.269	4.672	4.455	1.142	0.000	0.000	1.550
67	9.603	1.132	0.000	0.000	1.721	5.023	4.202	0.922	0.000	0.000	0.726
70	10.256	0.888	0.000	0.000	2.346	5.347	3.667	0.673	0.000	0.317	0.012
73	10.470	0.630	0.000	0.000	2.839	5.442	3.053	0.397	0.000	1.135	0.002
75	10.434	0.488	0.000	0.000	3.024	5.359	2.755	0.272	0.000	1.805	0.000
80	9.677	0.194	0.000	0.000	3.221	4.723	2.037	0.085	0.000	4.175	0.001
84	8.467	0.000	0.000	0.000	3.218	3.775	1.475	0.000	0.000	6.726	0.001
85	8.271	0.000	0.000	0.000	3.204	3.639	1.427	0.000	0.000	7.097	0.000
90	6.022	0.000	0.000	0.000	2.772	2.480	0.770	0.000	0.000	10.579	0.000
95	3.307	0.000	0.000	0.000	1.827	1.193	0.288	0.000	0.000	14.405	0.000
98	1.504	0.000	0.000	0.000	0.976	0.529	0.000	0.000	0.000	16.791	0.000
100	0.028	0.000	0.028	0.012	0.012	0.000	0.000	0.000	0.000	18.533	0.000

Table S16 Concentrations of the third mixture used to calculate the solvation energies of all species with COSMOtherm expressed in mole fractions for each concentration of sulfuric acid in mass percent %. Species not listed were included at infinite dilution.

H_2SO_4 %	HSO_4^-	SO_4^{2-}	$H_3SO_4^+$	$HS_2O_7^-$	H_3O^+	$H_5O_2^+$	$H_7O_3^+$	$H_9O_4^+$	$H_{11}O_5^+$	H_2SO_4	H_2O
5	0.0073	0.0028	0.0000	0.0000	0.0000	0.0000	0.0000	0.0000	0.0128	0.0000	0.9771
10	0.0158	0.0064	0.0000	0.0000	0.0000	0.0000	0.0000	0.0026	0.0261	0.0000	0.9491
15	0.0260	0.0113	0.0000	0.0000	0.0000	0.0000	0.0000	0.0063	0.0423	0.0000	0.9141
20	0.0384	0.0178	0.0000	0.0000	0.0000	0.0000	0.0000	0.0163	0.0577	0.0000	0.8699
25	0.0541	0.0262	0.0000	0.0000	0.0000	0.0000	0.0000	0.0346	0.0719	0.0000	0.8133
30	0.0736	0.0364	0.0000	0.0000	0.0000	0.0000	0.0102	0.0585	0.0775	0.0000	0.7438
35	0.0981	0.0478	0.0000	0.0000	0.0000	0.0000	0.0329	0.0877	0.0732	0.0000	0.6603
40	0.1292	0.0596	0.0000	0.0000	0.0000	0.0075	0.0650	0.1108	0.0651	0.0000	0.5629
45	0.1682	0.0699	0.0000	0.0000	0.0000	0.0246	0.1048	0.1259	0.0527	0.0000	0.4539
50	0.2145	0.0758	0.0000	0.0000	0.0000	0.0549	0.1525	0.1238	0.0349	0.0000	0.3436
55	0.2698	0.0760	0.0000	0.0000	0.0000	0.1012	0.1955	0.1062	0.0187	0.0000	0.2325
60	0.3318	0.0691	0.0000	0.0000	0.0141	0.1550	0.2107	0.0813	0.0090	0.0000	0.1290
64	0.3788	0.0568	0.0000	0.0000	0.0492	0.1959	0.1953	0.0519	0.0000	0.0000	0.0721
65	0.3851	0.0545	0.0000	0.0000	0.0543	0.2001	0.1908	0.0489	0.0000	0.0000	0.0664
67	0.4116	0.0485	0.0000	0.0000	0.0738	0.2153	0.1801	0.0395	0.0000	0.0000	0.0311
70	0.4363	0.0378	0.0000	0.0000	0.0998	0.2275	0.1560	0.0286	0.0000	0.0135	0.0005
73	0.4368	0.0263	0.0000	0.0000	0.1184	0.2270	0.1274	0.0166	0.0000	0.0474	0.0001
75	0.4323	0.0202	0.0000	0.0000	0.1253	0.2220	0.1141	0.0113	0.0000	0.0748	0.0000
80	0.4013	0.0081	0.0000	0.0000	0.1336	0.1959	0.0845	0.0035	0.0000	0.1731	0.0000
84	0.3578	0.0000	0.0000	0.0000	0.1360	0.1595	0.0623	0.0000	0.0000	0.2843	0.0001
85	0.3499	0.0000	0.0000	0.0000	0.1355	0.1540	0.0604	0.0000	0.0000	0.3002	0.0000
90	0.2662	0.0000	0.0000	0.0000	0.1225	0.1096	0.0340	0.0000	0.0000	0.4676	0.0000
95	0.1573	0.0000	0.0000	0.0000	0.0869	0.0567	0.0137	0.0000	0.0000	0.6853	0.0000
98	0.0760	0.0000	0.0000	0.0000	0.0493	0.0267	0.0000	0.0000	0.0000	0.8481	0.0000
100	0.0015	0.0000	0.0015	0.0006	0.0006	0.0000	0.0000	0.0000	0.0000	0.9957	0.0000

3.3 Gas-phase Enthalpy and Entropy of the Proton

The calculations require the Gibbs energy of the proton, which can be determined from principles of statistical thermodynamics. The proton gas is an ideal monoatomic gas, enabling the calculation of its gas-phase entropy using the Sackur-Tetrode equation.

$$S(\mathrm{H}^{+}) = R \ln\left(\frac{\exp\left(\frac{5}{2}\right)\ \mathrm{k_B} T}{p\lambda^{3}}\right) = 0.109\ \mathrm{kJ\,mol^{-1}}. \qquad \text{S8}$$

Here, R represents the ideal gas constant, k_B is the Boltzmann constant, λ is de Broglie wavelength and p symbolized the pressure.

The gas-phase enthalpy can be obtained from the ideal gas expression and amounts to

$$H(\mathrm{H}^{+}) = U + pV = \frac{5}{2}RT = 6.201\ \mathrm{kJ\,mol^{-1}}. \qquad \text{S9}$$

By using the Gibbs-Helmholtz equation, the combined contributions result in a free energy of −26.3 kJ mol^{-1} at 298.15 K.

3.4 Point Groups and Symmetry Number

For every geometry optimization, the point group determined by ORCA was verified and adjusted if necessary. The point group defines the symmetry number (sn), which affects the rotational entropy S_{rot} Adjustments were made to the rotational entropy based on the symmetry number of the selected point group. Table S17 shows the classification of symmetry numbers and point groups, following the work of Herzberg.[48]

Table S17 Point groups and their associated symmetry numbers sn used to assign the rotational entropy.

sn	Point groups[a]	
1	C_1, C_i, C_s	$C_{\infty v}$
2	C_2, C_{2v}, C_{2h}	$D_{\infty h}$, S_4
3	C_3, C_{3v}, C_{3h}	S_6
4	C_4, C_{4v}, C_{4h}	D_2, D_{2d}, $D_{2h} = V_h$
6	C_6, C_{6v}, C_{6h}	D_3, D_{3d}, D_{3h}
8	D_4, D_{4d}, D_{4h}	
12	D_6, D_{6d}, D_{6h}	T, T_d
24	O_h	

[a] Allocation of point groups to symmetry numbers was taken from the ORCA manual.[49]

3.5 Calculated Gas-Phase Energies

Table S18 Calculated gas-phase basicities at 1 bar and 298.15 K obtained through geometry optimization with DSD-BLYP/def2-TZVPP and DLPNO-CCSD(T)/CBS single-points on the optimized structures. All energies are given in kJ mol^{-1}.

Base	DSD-BLYP/ def2-TZVPP	DLPNO-CCSD(T)/ CBS	Experiment[50]
2,4,6-trinitroaniline	747.11	742.43	
2,4-dinitrotoluene	753.56	745.30	
m-nitrochlorobenzene	761.03	753.74	
m-nitrotoluene	786.20	778.48	
nitrobenzene	777.29	769.63	769.5
p-nitrochlorobenzene	775.57	764.01	
p-nitrofluorobenzene	773.10	763.32	
p-nitrotoluene	795.19	785.23	782.7

Table S19 Calculated gas-phase basicities at 1 bar and 298.15 K obtained through geometry optimization with DSD-PBEP86/def2-TZVPP and subsequent DLPNO-CCSD(T)/CBS single-points as well as CCSD(T)/CBS single-points on the optimized structures, along with the experimental values. All energies are provided in kJ mol^{-1}.

EH^+	→	$E + H^+$	DSD-PBEP86/ def2-TZVPP	DLPNO-CCSD(T)/CBS	CCSD(T)/CBS	Experiment
H_3O^+	→	$H_2O + H^+$	665.62	656.90	656.54	660.0[50]
$H_5O_2^+$	→	$2\ H_2O + H^+$	781.51	762.61	762.38	
$H_7O_3^+$	→	$3\ H_2O + H^+$	841.80	818.11	817.99	
$H_9O_4^+$	→	$4\ H_2O + H^+$	881.96	854.29		
$H_{11}O_5^+$	→	$5\ H_2O + H^+$	902.41	870.02		
H_2SO_4	→	$HSO_4^- + H^+$	1286.39	1270.52		1265.2[32]
HSO_4^-	→	$SO_4^- + H^+$	1895.25	1860.13		
$HSO_4^- + H_2O$	→	$SO_4^- \cdot H_2O + H^+$	1801.92	1778.11		
$HSO_4^- \cdot H_2O + H_2O$	→	$SO_4^- \cdot 2\ H_2O + H^+$	1747.86	1725.84		
$H_3SO_4^+$	→	$H_2SO_4 + H^+$	692.59	684.71		683 ± 3[31]
$H_5S_2O_8^+$	→	$H_4S_2O_8 + H^+$	741.37	732.71		
$H_5S_2O_8^+$	→	$2\ H_2SO_4 + H^+$	764.89	753.79		
$H_4S_2O_8$	→	$H_3S_2O_8^- + H^+$	1163.94	1154.47		
$H_2S_2O_7$	→	$HS_2O_7^- + H^+$	1182.89	1172.16		
$H_3S_2O_8^-$	→	$H_2S_2O_8^{2-} + H^+$	1646.56	1622.63		
$H_3S_2O_8^-$	→	$2\ HSO_4^- + H^+$	1432.36	1407.63		

Table S20 Calculated DSD-BLYP/def2-TZVPP gas-phase energies in Hartree (1 H = 2625.4996 kJ mol^{-1}) of the protonated water clusters labeled with their solvent number *n* and the isomer number in brackets (*i*). E_{tot}. refers to the total electronic energy. The enthalpy *H*, the Gibbs energy *G*, the entropy (already multiplied with the temperature $T{\cdot}S$) and the different contributions to the entropy (translational S_{trans}, vibrational S_{vib}, and rotational entropy S_{rot}) are calculated at 298.15 K and 1 bar pressure. The rotational entropy was selected according to the symmetry number of the determined point group (pg).

Species	pg	E_{tot}	H	S_{trans}	S_{vib}	S_{rot}	$T{\cdot}S$	G
H_2O	C_{2v}	-76.348251	-76.322912	0.016444	0.000003	0.004968	0.021415	-76.344327
H_3O^+	C_{3v}	-76.624573	-76.585932	0.016521	0.000081	0.005330	0.021932	-76.607864
$H_5O_2^+$	C_2	-153.030266	-152.966843	0.017465	0.002841	0.009184	0.029490	-152.996333
$H_7O_3^+$	C_1	-229.417594	-229.324942	0.018026	0.008690	0.011963	0.038679	-229.363621
$H_9O_4^+$	C_3	-305.798631	-305.677354	0.018427	0.015297	0.012168	0.045892	-305.723245
$H_{11}O_5^+$	C_1	-382.169772	-382.020974	0.018739	0.021643	0.014005	0.054387	-382.075361
H_2SO_4	C_2	-699.692165	-699.646357	0.018844	0.003337	0.011395	0.033575	-699.679932
$H_3SO_4^+$	C_{3v}	-699.976467	-699.919533	0.018858	0.004304	0.011047	0.034210	-699.953743
HSO_4^-	C_1	-699.179017	-699.145721	0.018829	0.003388	0.012014	0.034232	-699.179953
SO_4^{2-}	T_d	-698.438942	-698.418094	0.018814	0.001544	0.009620	0.029978	-698.448073
H_4SO_5	C_2	-775.987923	-775.913062	0.019083	0.005457	0.011924	0.036463	-775.949526
$H_5SO_5^+$	C_s	-776.308032	-776.220951	0.019095	0.005810	0.012593	0.037497	-776.258448
$H_4S_2O_8$	C_1	-1399.414592	-1399.321096	0.019825	0.013505	0.014393	0.047724	-1399.368820
$H_5S_2O_8^+$	C_1	-1399.713848	-1399.610066	0.019833	0.016793	0.014520	0.051146	-1399.661212
$H_3S_2O_8^-$	C_s	-1398.949684	-1398.869539	0.019818	0.011775	0.014349	0.045942	-1398.915480
$H_2S_2O_8^{2-}$	C_2	-1398.300007	-1398.231117	0.019811	0.013441	0.013954	0.047206	-1398.278323
$H_2S_2O_7$	C_2	-1323.030246	-1322.964969	0.019689	0.008687	0.013237	0.041614	-1323.006583
$HS_2O_7^-$	C_1	-1322.556457	-1322.503799	0.019681	0.008624	0.013923	0.042228	-1322.546027
$HSO_4^-{\cdot}\ H_2O$	C_s	-775.555154	-775.493192	0.019070	0.007072	0.012837	0.038979	-775.532170
$SO_4^{2-}{\cdot}\ H_2O$	C_{2v}	-774.839155	-774.790521	0.019058	0.006119	0.012249	0.037426	-774.827947
$SO_4^{2-}{\cdot}\ 2\ H_2O$	C_s	-851.233101	-851.156539	0.019265	0.011308	0.013644	0.044217	-851.200756

Table S21 DLPNO-CCSD(T)/CBS and CCSD(T)/CBS gas-phase energies obtained through single-point calculations on the optimized DSD-PBEP86/def2-TZVPP structures. in Hartree (1 H = 2625.4996 kJ mol^{-1}). The Gibbs energies G at 1 bar and 298.15 K were obtained from the total electronic energies E_{tot} of the respective coupled cluster calculations and the enthalpic and entropic contributions acquired through the geometry optimizations with DSD-PBEP86/def2-TZVPP.

Species	DLPNO-CCSD(T)/CBS		CCSD(T)/CBS	
	E_{tot}	G	E_{tot}	G
H_2O	-76.376540	-76.372616	-76.376667	-76.372743
H_2O^+	-76.649542	-76.632834	-76.649522	-76.632813
$H_5O_2^+$	-153.079643	-153.045711	-153.079802	-153.045870
$H_7O_3^+$	-229.493440	-229.439467	-229.493766	-229.439793
$H_9O_4^+$	-305.901247	-305.825861		
$H_{11}O_5^+$	-382.298879	-382.204469		
H_2SO_4	-699.655621	-699.643388		
$H_3SO_4^+$	-699.936921	-699.914197	-699.939377	-699.916653
HSO_4^-	-699.148521	-699.149457		
SO_4^{2-}	-698.421823	-698.430953		
H_4SO_5	-775.977075	-775.938677		
$H_5SO_5^+$	-776.294513	-776.244928		
$H_4S_2O_8$	-1399.340574	-1399.294802		
$H_5S_2O_8^+$	-1399.636532	-1399.583896		
$H_3S_2O_8^-$	-1398.879274	-1398.845070		
$H_2S_2O_8^{2-}$	-1398.238709	-1398.217025		
$H_2S_2O_7$	-1322.928343	-1322.904679		
$HS_2O_7^-$	-1322.458639	-1322.448209		
$HSO_4^- \cdot H_2O$	-775.550154	-775.527170		
$HSO_4^- \cdot 2\ H_2O$	-851.947837	-851.904041		
$SO_4^{2-} \cdot H_2O$	-774.846018	-774.834810		
$SO_4^{2-} \cdot 2\ H_2O$	-851.264776	-851.232431		

Table S22 Calculated DSD-BLYP/def2-TZVPP gas-phase energies in Hartree (1 H = 2625.4996 kJ mol^{-1}) of the protonated water clusters labeled with their solvent number *n* and the isomer number in brackets (*i*). E_{tot}. refers to the total electronic energy. The enthalpy *H*, the Gibbs energy *G*, the entropy (already multiplied with the temperature $T{\cdot}S$) and the different contributions to the entropy (translational S_{trans}, vibrational S_{vib}, and rotational entropy S_{rot}) are calculated at 298.15 K and 1 bar pressure. The rotational entropy was selected according to the symmetry number of the determined point group (pg).

Species	pg	E_{tot}	H	S_{trans}	S_{vib}	S_{rot}	$T{\cdot}S$	G
2-4-6-trinitroaniline	C_{2v}	-900.508483	-900.369691	0.020039	0.018476	0.014859	0.053374	-900.423065
2-4-6-trinitroanilineH$^+$	C_s	-900.814750	-900.663759	0.020045	0.018310	0.015526	0.053882	-900.717641
2-4-dinitrotoluene	C_1	-680.070842	-679.925072	0.019720	0.014118	0.014837	0.048676	-679.973748
2-4-dinitrotolueneH$^+$	C_1	-680.379418	-680.221652	0.019728	0.014548	0.014851	0.049128	-680.270780
m-nitrochlorobenzene	C_s	-895.796729	-895.693932	0.019515	0.008653	0.014364	0.042533	-895.736464
m-nitrochlorobenzeneH$^+$	C_s	-896.108533	-895.993406	0.019524	0.009032	0.014380	0.042936	-896.036342
m-nitrotoluene	C_1	-475.686428	-475.545602	0.019319	0.010192	0.014020	0.043530	-475.589132
m-nitrotolueneH$^+$	C_s	-476.008285	-475.854989	0.019329	0.010238	0.014040	0.043607	-475.898596
nitrobenzene	C_{2v}	-436.409645	-436.298315	0.019166	0.006533	0.012909	0.038608	-436.336923
nitrobenzeneH$^+$	C_s	-436.727198	-436.603341	0.019177	0.006887	0.013589	0.039654	-436.642994
p-nitrochlorobenzene	C_{2v}	-895.797826	-895.694990	0.019515	0.008638	0.013616	0.041769	-895.736759
p-nitrochlorobenzeneH$^+$	C_s	-896.114678	-895.999385	0.019524	0.008978	0.014287	0.042790	-896.042174
p-nitrofluorobenzene	C_{2v}	-535.610980	-535.507090	0.019359	0.007859	0.013311	0.040530	-535.547619
p-nitrofluorobenzeneH$^+$	C_s	-535.926982	-535.810511	0.019369	0.008228	0.013986	0.041583	-535.852093
p-nitrotoluene	C_1	-475.686720	-475.545916	0.019319	0.010332	0.013959	0.043610	-475.589526
p-nitrotolueneH$^+$	C_1	-476.011556	-475.858338	0.019329	0.010768	0.013981	0.044078	-475.902416

3.6 Calculated Solvation Energies

The following section presents the solvation energies of the proton $\Delta_{solv}G(H^+)$ obtained from different thermodynamic cycles, along with the solvation energies of all the species used for those cycles. Certain proton solvation energies are given at the default state $\Delta_{solv}G°(H^+)$ (pH = 0), while others are provided at specific pH values of pure sulfuric acid or the respective mixtures of sulfuric acid and water $\Delta_{solv}G(H^+)$. The states can be distinguished by the superscript circle. It is crucial to pay attention to this superscript when studying these tables in order to differentiate between the states. However, the solvation energies of all the species (indicator bases, sulfuric acid species, protonated water clusters) are provided at the default state of $\Delta_{solv}G°$.

Table S23 Standard solvation energies $\Delta_{solv}G°$ (transition of 1 bar gas into ideal 1 mol L^{-1}) of the indicator bases obtained with the CPCM. Total electronic energy E_{tot} of the CPCM optimization at the DSD-BLYP/def2-TZVPP level of theory and the subsequent CPCM DLPNO-CCSD(T)/CBS single-point in Hartree (1 H = 2625.4996 kJ mol^{-1}). Solvation energies $\Delta_{solv}G°$ of the indicator bases in kJ mol^{-1} are obtained as the difference of the respective total electronic energies in solution E_{tot} and in the total electronic energy in the gas-phase E_{tot}.

Base	DSD-BLYP/def2-TZVPP		DLPNO-CCSD(T)/CBS	
	E_{tot} H	$\Delta_{solv}G°$ kJ mol^{-1}	E_{tot} H	$\Delta_{solv}G°$ kJ mol^{-1}
2-4-6-trinitroaniline	-900.526784	-40.09	-900.218719	-39.12
2-4-6-trinitroanilineH$^+$	-900.921526	-272.38	-900.610948	-269.50
2-4-dinitrotoluene	-680.084930	-29.03	-679.839034	-29.22
2-4-dinitrotolueneH$^+$	-680.476555	-247.07	-680.228551	-249.99
m-nitrochlorobenzene	-895.805668	-15.51	-895.419405	-15.20
m-nitrochlorobenzeneH$^+$	-896.198527	-228.32	-895.810452	-230.54
m-nitrotoluene	-475.695901	-16.91	-475.510099	-16.89
m-nitrotolueneH$^+$	-476.093266	-215.16	-475.904951	-216.25
nitrobenzene	-436.418890	-16.31	-436.248462	-16.45
nitrobenzeneH$^+$	-436.815170	-223.01	-436.642465	-224.83
p-nitrochlorobenzene	-895.806959	-16.02	-895.420614	-15.93
p-nitrochlorobenzeneH$^+$	-896.202400	-222.35	-895.813232	-226.41
p-nitrofluorobenzene	-535.620551	-17.17	-535.431552	-17.43
p-nitrofluorobenzeneH$^+$	-536.016759	-227.75	-535.824741	-229.86
p-nitrotoluene	-475.696551	-17.85	-475.510574	-17.47
p-nitrotolueneH$^+$	-476.095971	-213.67	-475.906618	-214.38

3.6.1 Solvation energies obtained from the concentrations of the first mixture

Table S24 Gibbs energies of solvation of the proton $\Delta_{solv}G(H^+)$ at 298.15 K and 1 bar obtained from the first mixture through the respective thermodynamic cycles. Listed are the main thermodynamic cycles with their identified by their numbers and the mass percentage of sulfuric acid %. All energies are given in kJ mol^{-1}.

H_2SO_4 %	1a	1b	1c	2	3a	3b	3c	3d
5	-1077.36	-1100.86	-1102.74	-966.49	-941.58	-970.47	-1015.75	-1016.39
10	-1073.88	-1097.93	-1100.40	-968.55	-943.36	-971.95	-1015.73	-1016.22
15	-1070.62	-1095.04	-1098.01	-970.09	-944.79	-973.08	-1015.87	-1016.11
20	-1067.10	-1091.81	-1095.22	-970.97	-945.72	-973.68	-1015.81	-1015.70
25	-1063.46	-1088.38	-1092.17	-971.59	-946.52	-974.09	-1015.75	-1015.18
30	-1059.55	-1084.63	-1088.75	-971.91	-947.21	-974.26	-1015.56	-1014.40
35	-1055.45	-1080.67	-1085.09	-972.13	-948.06	-974.40	-1015.35	-1013.47
40	-1051.06	-1076.71	-1081.60	-972.59	-949.39	-974.92	-1015.43	-1012.76
45	-1046.69	-1072.93	-1078.44	-973.49	-951.55	-975.99	-1015.90	-1012.29
50	-1042.57	-1069.63	-1075.91	-975.10	-954.92	-977.92	-1016.77	-1012.12
55	-1038.39	-1067.41	-1075.12	-978.87	-960.93	-982.56	-1019.37	-1014.35
60	-1034.18	-1065.77	-1075.25	-984.33	-969.29	-989.59	-1022.71	-1017.80
64	-1030.74	-1065.14	-1076.43	-991.32	-979.27	-998.98	-1026.52	-1022.33
65	-1030.39	-1065.16	-1076.68	-992.23	-980.57	-1000.29	-1026.96	-1022.92
67	-1028.70	-1065.34	-1078.02	-996.60	-986.52	-1006.54	-1029.50	-1026.22
70	-1024.99	-1064.78	-1079.53	-1003.70	-996.43	-1017.08	-1033.50	-1031.45
73	-1020.91	-1063.69	-1080.35	-1013.02	-1008.81	-1030.31	-1038.08	-1037.55
75	-1019.11	-1063.65	-1081.46	-1019.61	-1017.05	-1039.34	-1041.17	-1041.77
80	-1015.98	-1063.77	-1084.03	-1033.58	-1034.51	-1058.24	-1048.34	-1051.55
84	-1006.45	-1054.92	-1076.76	-1029.06	-1033.05	-1053.32	-1043.67	-1046.05
85	-1004.72	-1053.33	-1075.43	-1027.97	-1032.45	-1052.01	-1042.45	-1044.62
90	-984.52	-1036.18	-1062.08	-1016.87	-1028.18	-1040.13	-1025.11	-1025.77
95	-963.97	-1024.43	-1060.47	-1002.07	-1017.70	-1027.56	-1033.47	-1032.95
98	-942.86	-1006.05	-1045.46	-972.41	-986.01	-995.30	-1026.19	-1022.37
100	-954.48	-1012.19	-1045.98	-939.16	-949.90	-957.91	-1017.25	-1006.22

The proton solvation energies $\Delta_{solv}G(H^+)$ calculated through the thermodynamic cycles that were not considered as potential determining species in the aqueous solutions of sulfuric acid are presented in Table S25.

Table S25 Gibbs energies of solvation of the proton $\Delta_{solv}G(H^+)$ at 298.15 K and 1 bar obtained from the first mixture through the thermodynamic cycles of different sulfuric acid derivatives $EH^+ \rightarrow E + H^+$, which are listed here as EH^+/E. All energies are given in kJ mol^{-1} in relation to the mass percentage of sulfuric acid %.

H_2SO_4 %	H_2SO_4 / HSO_4^-	$H_4S_2O_8$ / $H_3S_2O_8^-$	$H_2S_2O_7$ / $HS_2O_7^-$	$H_5SO_5^+$ / H_4SO_5
5	-1030.39	-1016.14	-1007.82	-1045.06
10	-1030.49	-1016.65	-1008.41	-1045.41
15	-1030.65	-1017.14	-1009.01	-1045.79
20	-1030.53	-1017.28	-1009.26	-1045.89
25	-1030.32	-1017.31	-1009.44	-1045.92
30	-1029.88	-1017.14	-1009.44	-1045.77
35	-1029.32	-1016.94	-1009.48	-1045.57
40	-1029.04	-1017.11	-1009.81	-1045.74
45	-1029.05	-1017.77	-1010.66	-1046.31
50	-1029.44	-1019.10	-1012.25	-1047.48
55	-1032.10	-1023.20	-1016.33	-1051.32
60	-1036.10	-1029.19	-1022.35	-1057.21
64	-1041.54	-1036.84	-1030.03	-1065.36
65	-1042.28	-1037.88	-1031.08	-1066.51
67	-1046.23	-1042.93	-1036.19	-1072.17
70	-1052.76	-1051.36	-1045.13	-1081.67
73	-1060.55	-1061.52	-1056.57	-1092.93
75	-1066.00	-1068.55	-1064.62	-1100.57
80	-1078.28	-1084.49	-1083.73	-1116.80
84	-1072.12	-1080.29	-1081.98	-1112.21
85	-1070.46	-1078.92	-1080.92	-1110.86
90	-1048.72	-1060.38	-1065.71	-1094.64
95	-1036.21	-1029.09	-1035.79	-1070.34
98	-1017.88	-1000.29	-1010.44	-1039.29
100	-1001.81	-978.36	-988.08	-1007.71

Table S26 Gibbs energies of solvation of the proton $\Delta_{solv}G(H^+)$ at 298.15 K and 1 bar obtained from the first mixture through the thermodynamic cycles of the protonated water clusters $EH^+ \rightarrow E + H^+$, which are listed here as EH^+/E. All energies are given in kJ mol^{-1} in relation to the mass percentage of sulfuric acid %.

H_2SO_4 %	H_3O^+ / H_2O	$H_5O_2^+$ / 2 H_2O	$H_7O_3^+$ / 3 H_2O	$H_9O_4^+$ / 4 H_2O	$H_{11}O_5^+$ / 5 H_2O
5	-1043.58	-1053.64	-1049.45	-1041.47	-1027.49
10	-1046.16	-1055.09	-1050.41	-1042.27	-1028.29
15	-1048.33	-1056.49	-1051.38	-1043.16	-1029.21
20	-1049.82	-1057.51	-1052.03	-1043.78	-1029.87
25	-1051.01	-1058.40	-1052.60	-1044.36	-1030.49
30	-1051.75	-1059.00	-1052.91	-1044.71	-1030.89
35	-1052.14	-1059.40	-1053.05	-1044.92	-1031.11
40	-1052.27	-1059.84	-1053.35	-1045.28	-1031.40
45	-1052.22	-1060.32	-1053.77	-1045.78	-1031.72
50	-1052.09	-1060.92	-1054.39	-1046.48	-1032.14
55	-1052.77	-1063.30	-1057.42	-1049.76	-1035.11
60	-1053.82	-1066.78	-1061.99	-1054.72	-1039.74
64	-1055.76	-1071.73	-1068.29	-1061.41	-1046.09
65	-1055.95	-1072.38	-1069.14	-1062.31	-1046.94
67	-1057.40	-1075.98	-1073.54	-1066.91	-1051.37
70	-1059.57	-1081.89	-1080.54	-1074.12	-1058.23
73	-1063.29	-1088.77	-1088.36	-1082.08	-1065.72
75	-1066.42	-1093.48	-1093.62	-1087.39	-1070.72
80	-1072.94	-1102.99	-1104.13	-1098.01	-1080.68
84	-1065.19	-1095.51	-1096.35	-1090.24	-1072.97
85	-1063.65	-1093.85	-1094.59	-1088.48	-1071.24
90	-1044.49	-1072.94	-1072.21	-1065.13	-1047.57
95	-1023.64	-1053.49	-1050.50	-1041.80	-1024.85
98	-997.41	-1024.28	-1019.52	-1010.59	-994.83
100	-962.51	-986.83	-986.15	-979.31	-964.15

Table S27 Solvation energies $\Delta_{solv}G$ of all species included in the first mixture with the concentrations given in Section 3.2 for each concentration of sulfuric acid in mass percent %. All energies are given in kJ mol^{-1} at 298.15 K and a pressure of 1 bar.

%	H_2SO_4	$H_3SO_4^+$	HSO_4^-	H_2O	H_3O^+	$H_5O_2^+$	$H_7O_3^+$	$H_9O_4^+$	$H_{11}O_5^+$	SO_4^{2-}	$HS_2O_7^-$
5	-60.16	-341.94	-300.29	-20.89	-407.57	-332.82	-294.02	-270.76	-261.94	-1083.06	-223.08
10	-60.38	-344.22	-300.41	-20.70	-409.96	-333.88	-294.40	-270.80	-261.78	-1086.66	-223.32
15	-60.52	-345.91	-300.38	-20.47	-411.89	-334.81	-294.67	-270.74	-261.52	-1089.90	-223.41
20	-60.56	-346.82	-300.55	-20.20	-413.11	-335.29	-294.51	-270.29	-260.84	-1093.58	-223.64
25	-60.47	-347.35	-300.67	-19.89	-414.00	-335.58	-294.15	-269.63	-259.92	-1097.34	-223.76
30	-60.18	-347.38	-300.82	-19.55	-414.40	-335.50	-293.46	-268.64	-258.64	-1101.40	-223.77
35	-59.63	-347.05	-300.83	-19.21	-414.45	-335.21	-292.57	-267.47	-257.14	-1105.51	-223.41
40	-58.67	-346.56	-300.15	-18.94	-414.31	-335.11	-292.06	-266.76	-256.08	-1109.23	-222.02
45	-57.26	-346.04	-298.72	-18.76	-414.08	-335.23	-291.93	-266.53	-255.50	-1112.16	-219.44
50	-55.32	-345.71	-296.40	-18.72	-413.91	-335.76	-292.45	-267.09	-255.74	-1113.96	-215.35
55	-52.75	-346.91	-291.17	-18.83	-414.69	-338.35	-295.79	-270.78	-259.23	-1112.91	-207.41
60	-49.75	-349.37	-284.16	-19.09	-416.00	-342.35	-301.14	-276.77	-265.15	-1110.11	-196.57
64	-46.96	-353.57	-275.94	-19.48	-418.33	-348.08	-308.61	-285.04	-273.47	-1105.33	-183.81
65	-46.63	-354.16	-274.87	-19.56	-418.61	-348.89	-309.69	-286.24	-274.70	-1104.62	-182.11
67	-45.47	-357.36	-269.76	-19.84	-420.34	-353.06	-314.96	-292.00	-280.57	-1101.18	-174.42
70	-43.96	-362.95	-261.72	-20.44	-423.10	-360.15	-323.73	-301.57	-290.39	-1096.86	-162.05
73	-42.90	-371.21	-252.87	-21.30	-427.68	-368.76	-334.15	-312.99	-302.20	-1092.09	-148.14
75	-42.64	-377.55	-247.16	-21.97	-431.49	-374.81	-341.42	-320.98	-310.55	-1088.18	-139.11
80	-43.74	-392.62	-235.98	-23.88	-439.92	-388.15	-357.66	-339.26	-330.07	-1080.14	-121.24
84	-43.79	-388.14	-242.19	-24.90	-433.18	-382.70	-352.93	-335.55	-327.44	-1095.87	-125.98
85	-43.57	-386.83	-243.62	-25.01	-431.75	-381.26	-351.50	-334.22	-326.26	-1099.04	-127.21
90	-39.79	-371.94	-261.58	-27.52	-415.10	-365.37	-336.65	-320.91	-315.14	-1137.19	-141.31
95	-40.13	-357.49	-274.43	-31.90	-398.64	-354.69	-328.10	-315.13	-314.35	-1170.59	-172.17
98	-41.26	-328.96	-293.89	-32.24	-372.75	-326.15	-298.13	-285.27	-286.02	-1211.17	-198.39
100	-41.59	-296.05	-310.30	-32.11	-337.72	-288.45	-264.38	-253.47	-254.70	-1215.95	-220.69

Table S28 Solvation energies $\Delta_{solv}G$ of the first mixture of all species included at infinite dilution relative to the mass percentage of sulfuric acid %. All energies are given in kJ mol^{-1} at 298.15 K and a pressure of 1 bar.

%	$H_4S_2O_8$	$H_5S_2O_8^+$	$H_3S_2O_8^-$	$H_2S_2O_8^{2-}$	$SO_4^{2-} \cdot H_2O$	$SO_4^{2-} \cdot 2\ H_2O$	$HSO_4^- \cdot H_2O$	$H_5SO_5^+$	H_4SO_5	$H_2S_2O_7$
5	-70.37	-308.12	-208.70	-814.94	-998.43	-923.28	-279.29	-322.81	-55.52	-58.74
10	-71.09	-310.33	-208.91	-815.32	-1001.29	-925.55	-279.41	-322.61	-54.97	-59.56
15	-71.68	-312.05	-209.01	-815.53	-1003.92	-927.69	-279.40	-322.31	-54.28	-60.25
20	-72.08	-313.04	-209.27	-816.21	-1007.05	-930.38	-279.56	-321.58	-53.45	-60.74
25	-72.29	-313.67	-209.45	-816.90	-1010.29	-933.24	-279.67	-320.59	-52.43	-61.03
30	-72.23	-313.78	-209.56	-817.79	-1013.85	-936.46	-279.82	-319.19	-51.18	-61.05
35	-71.84	-313.53	-209.37	-818.53	-1017.47	-939.77	-279.82	-317.44	-49.63	-60.72
40	-70.74	-312.95	-208.10	-817.97	-1020.49	-942.32	-279.15	-315.88	-47.90	-59.67
45	-69.01	-312.29	-205.71	-816.05	-1022.66	-943.94	-277.78	-314.41	-45.87	-57.94
50	-66.56	-311.77	-201.93	-812.43	-1023.60	-944.26	-275.60	-313.22	-43.50	-55.44
55	-62.80	-312.65	-194.08	-802.36	-1020.70	-939.86	-270.32	-314.94	-41.39	-51.57
60	-58.13	-315.00	-183.41	-788.25	-1015.59	-932.86	-263.19	-318.72	-39.27	-46.76
64	-53.12	-319.39	-170.76	-771.06	-1008.39	-923.66	-254.77	-325.20	-37.61	-41.68
65	-52.48	-320.05	-169.07	-768.78	-1007.38	-922.40	-253.69	-326.23	-37.48	-41.02
67	-49.84	-323.67	-161.39	-757.80	-1002.37	-916.16	-248.50	-331.51	-37.10	-38.44
70	-46.20	-330.57	-149.31	-740.49	-995.48	-907.31	-240.57	-340.46	-36.55	-35.02
73	-43.23	-340.83	-136.19	-721.27	-988.59	-899.04	-232.26	-351.36	-36.19	-32.54
75	-41.93	-348.55	-127.86	-708.72	-983.59	-893.38	-227.03	-359.17	-36.37	-31.56
80	-42.69	-368.21	-112.67	-683.75	-974.21	-883.32	-217.63	-376.77	-37.73	-32.81
84	-46.23	-366.84	-120.41	-696.99	-990.28	-900.29	-226.31	-371.27	-36.82	-35.80
85	-46.51	-365.81	-122.06	-700.07	-993.41	-903.49	-228.08	-369.60	-36.50	-35.97
90	-46.55	-353.96	-140.64	-737.50	-1031.03	-941.71	-250.43	-350.36	-33.48	-34.86
95	-49.32	-344.17	-174.70	-764.38	-1060.01	-968.99	-271.72	-332.24	-39.66	-35.80
98	-52.16	-314.74	-206.35	-806.61	-1098.19	-1005.40	-292.78	-304.20	-42.67	-36.66
100	-54.10	-279.30	-230.22	-846.63	-1108.33	-1022.16	-310.20	-274.93	-44.98	-36.61

3.6.2 Solvation energies obtained from the concentrations of the second mixture

Table S29 Gibbs energies of solvation of the proton $\Delta_{solv}G(H^+)$ at 298.15 K and 1 bar obtained from the second mixture through the respective thermodynamic cycles. Listed are the main thermodynamic cycles with their identified by their numbers and the mass percentage of sulfuric acid %. All energies are given in kJ mol^{-1}.

%	1a	1b	1c	2	3a	3b	3c	3d
5	-1077.36	-1100.86	-1102.74	-966.49	-941.58	-970.47	-1015.75	-1016.39
10	-1073.74	-1097.83	-1100.30	-968.46	-943.26	-971.86	-1015.62	-1016.11
15	-1070.47	-1094.96	-1097.93	-970.00	-944.68	-973.00	-1015.74	-1016.00
20	-1067.02	-1091.80	-1095.21	-970.97	-945.68	-973.69	-1015.72	-1015.65
25	-1063.42	-1088.41	-1092.20	-971.60	-946.49	-974.12	-1015.66	-1015.15
30	-1059.53	-1084.82	-1089.03	-972.16	-947.41	-974.58	-1015.76	-1014.70
35	-1055.23	-1080.81	-1085.44	-972.36	-948.20	-974.74	-1015.63	-1013.87
40	-1051.02	-1076.92	-1082.00	-972.88	-949.62	-975.30	-1015.84	-1013.24
45	-1046.40	-1073.20	-1079.09	-974.18	-952.25	-976.86	-1016.83	-1013.44
50	-1042.00	-1070.24	-1077.26	-976.77	-956.62	-980.01	-1018.78	-1014.83
55	-1037.84	-1067.80	-1076.10	-980.37	-962.49	-984.48	-1021.04	-1016.67
60	-1033.46	-1066.48	-1076.81	-986.33	-971.55	-992.36	-1025.38	-1021.26
64	-1028.43	-1065.16	-1078.01	-993.74	-982.38	-1002.75	-1030.56	-1027.25
65	-1027.75	-1064.98	-1078.18	-994.74	-983.84	-1004.24	-1031.22	-1028.08
67	-1024.99	-1063.95	-1078.35	-998.62	-989.45	-1009.93	-1033.46	-1030.85
70	-1021.42	-1062.37	-1078.11	-1004.00	-997.43	-1017.81	-1035.94	-1033.96
73	-1017.67	-1060.48	-1077.54	-1009.02	-1005.02	-1025.08	-1037.85	-1036.39
75	-1014.91	-1058.72	-1076.60	-1010.92	-1008.36	-1027.98	-1038.42	-1037.15
80	-1006.10	-1051.96	-1071.88	-1012.15	-1013.47	-1030.73	-1036.60	-1035.23
84	-996.56	-1044.02	-1065.75	-1009.12	-1014.70	-1028.51	-1030.01	-1028.05
85	-995.12	-1042.90	-1064.97	-1008.60	-1014.73	-1028.06	-1029.08	-1027.03
90	-980.67	-1033.34	-1060.46	-1003.06	-1014.47	-1024.23	-1024.50	-1021.94
95	-963.66	-1022.58	-1057.32	-990.33	-1003.93	-1013.44	-1031.63	-1028.17
98	-943.80	-1006.30	-1044.79	-969.09	-982.24	-991.32	-1023.43	-1019.04
100	-954.48	-1012.19	-1045.98	-939.16	-949.90	-957.91	-1017.25	-1006.22

The proton solvation energies $\Delta_{solv}G(H^+)$ calculated through the thermodynamic cycles that were not considered as potential determining species in the aqueous solutions of sulfuric acid are presented in Table S30.

Table S30 Gibbs energies of solvation of the proton $\Delta_{solv}G(H^+)$ at 298.15 K and 1 bar obtained from the second mixture through the thermodynamic cycles of different sulfuric acid derivatives $EH^+ \rightarrow E + H^+$, which are listed here as EH^+/E. All energies are given in kJ mol^{-1} in relation to the mass percentage of sulfuric acid %.

%	H_2SO_4 / HSO_4^-	$H_4S_2O_8$ / $H_3S_2O_8^-$	$H_2S_2O_7$ / $HS_2O_7^-$	$H_5SO_5^+$ / H_4SO_5
5	-1030.39	-1016.14	-1007.82	-1045.06
10	-1030.39	-1016.55	-1008.30	-1045.32
15	-1030.55	-1017.05	-1008.89	-1045.72
20	-1030.50	-1017.26	-1009.21	-1045.90
25	-1030.30	-1017.31	-1009.39	-1045.95
30	-1030.19	-1017.46	-1009.66	-1046.11
35	-1029.74	-1017.31	-1009.66	-1045.99
40	-1029.53	-1017.53	-1010.09	-1046.18
45	-1030.14	-1018.84	-1011.51	-1047.31
50	-1031.92	-1021.66	-1014.35	-1049.87
55	-1034.23	-1025.43	-1018.23	-1053.47
60	-1039.23	-1032.27	-1025.17	-1060.26
64	-1045.94	-1040.94	-1034.09	-1069.51
65	-1046.89	-1042.17	-1035.36	-1070.86
67	-1050.29	-1046.64	-1040.13	-1075.91
70	-1054.48	-1052.63	-1046.92	-1082.61
73	-1058.07	-1058.22	-1053.66	-1088.65
75	-1059.32	-1060.60	-1056.88	-1091.07
80	-1058.06	-1062.27	-1061.00	-1092.67
84	-1050.45	-1057.08	-1058.11	-1088.21
85	-1049.17	-1055.94	-1057.22	-1087.27
90	-1037.66	-1041.07	-1044.29	-1075.88
95	-1030.16	-1019.18	-1026.33	-1057.81
98	-1015.20	-997.90	-1007.88	-1035.89
100	-1001.81	-978.36	-988.08	-1007.71

Table S31 Gibbs energies of solvation of the proton $\Delta_{solv}G(H^+)$ at 298.15 K and 1 bar obtained from the second mixture through the thermodynamic cycles of the protonated water clusters $EH^+ \rightarrow E + H^+$, which are listed here as EH^+/E. All energies are given in kJ mol^{-1} in relation to the mass percentage of sulfuric acid %.

%	H_3O^+ / H_2O	$H_5O_2^+$ / 2 H_2O	$H_7O_3^+$ / 3 H_2O	$H_9O_4^+$ / 4 H_2O	$H_{11}O_5^+$ / 5 H_2O
5	-1043.58	-1053.64	-1049.45	-1041.47	-1027.49
10	-1046.07	-1055.00	-1050.32	-1042.18	-1028.19
15	-1048.21	-1056.40	-1051.30	-1043.07	-1029.10
20	-1049.81	-1057.51	-1052.04	-1043.77	-1029.84
25	-1051.01	-1058.42	-1052.61	-1044.35	-1030.47
30	-1051.91	-1059.29	-1053.24	-1045.02	-1031.15
35	-1052.24	-1059.72	-1053.46	-1045.30	-1031.43
40	-1052.46	-1060.22	-1053.81	-1045.73	-1031.81
45	-1052.62	-1061.16	-1054.85	-1046.92	-1032.86
50	-1053.15	-1062.94	-1057.02	-1049.30	-1035.03
55	-1053.72	-1065.08	-1059.75	-1052.29	-1037.74
60	-1054.98	-1069.35	-1065.26	-1058.23	-1043.37
64	-1056.80	-1075.15	-1072.50	-1065.89	-1050.66
65	-1057.01	-1075.95	-1073.50	-1066.94	-1051.65
67	-1057.99	-1078.95	-1077.11	-1070.66	-1055.15
70	-1059.11	-1082.39	-1081.23	-1074.92	-1059.07
73	-1059.82	-1084.99	-1084.39	-1078.17	-1061.99
75	-1059.38	-1085.47	-1085.11	-1078.95	-1062.65
80	-1054.95	-1082.45	-1082.26	-1076.08	-1059.51
84	-1046.13	-1073.90	-1073.25	-1066.61	-1049.67
85	-1044.84	-1072.53	-1071.77	-1065.05	-1048.05
90	-1031.21	-1058.64	-1056.56	-1048.48	-1031.06
95	-1015.93	-1044.78	-1041.08	-1032.43	-1016.16
98	-994.61	-1020.89	-1016.24	-1007.37	-991.67
100	-962.51	-986.83	-986.15	-979.31	-964.15

Table S32 Solvation energies $\Delta_{solv}G$ of all species included in the second mixture with the concentrations given in Section 3.2 for each concentration of sulfuric acid in mass percent %. All energies are given in kJ mol^{-1} at 298.15 K and a pressure of 1 bar.

%	H_2SO_4	$H_3SO_4^+$	HSO_4^-	H_2O	H_3O^+	$H_5O_2^+$	$H_7O_3^+$	$H_9O_4^+$	$H_{11}O_5^+$	SO_4^{2-}	$HS_2O_7^-$
5	-60.16	-341.94	-300.29	-20.89	-407.57	-332.82	-294.02	-270.76	-261.94	-1083.06	-223.08
10	-60.38	-344.13	-300.51	-20.71	-409.87	-333.81	-294.33	-270.72	-261.71	-1086.90	-223.41
15	-60.51	-345.80	-300.48	-20.48	-411.79	-334.75	-294.62	-270.70	-261.48	-1090.14	-223.48
20	-60.56	-346.82	-300.58	-20.21	-413.13	-335.33	-294.56	-270.34	-260.89	-1093.68	-223.65
25	-60.47	-347.36	-300.68	-19.91	-414.01	-335.63	-294.23	-269.70	-260.00	-1097.40	-223.75
30	-60.14	-347.58	-300.47	-19.60	-414.60	-335.88	-293.92	-269.12	-259.12	-1101.07	-223.37
35	-59.56	-347.21	-300.33	-19.28	-414.61	-335.68	-293.19	-268.14	-257.82	-1105.23	-222.89
40	-58.62	-346.80	-299.61	-18.99	-414.55	-335.58	-292.66	-267.39	-256.72	-1108.72	-221.50
45	-57.11	-346.58	-297.49	-18.79	-414.51	-336.12	-293.10	-267.78	-256.78	-1111.22	-218.11
50	-55.07	-347.14	-293.67	-18.71	-414.96	-337.76	-295.05	-269.86	-258.57	-1111.80	-212.36
55	-52.59	-348.25	-288.87	-18.79	-415.61	-340.05	-298.00	-273.15	-261.65	-1111.17	-204.83
60	-49.63	-351.25	-280.91	-19.07	-417.15	-344.88	-304.35	-280.21	-268.69	-1107.59	-193.02
64	-46.94	-355.97	-271.52	-19.55	-419.45	-351.65	-313.05	-289.81	-278.41	-1103.22	-179.11
65	-46.65	-356.68	-270.27	-19.64	-419.75	-352.63	-314.32	-291.22	-279.85	-1102.66	-177.24
67	-45.59	-359.50	-265.82	-19.98	-421.07	-356.31	-318.94	-296.30	-285.04	-1100.95	-170.36
70	-44.41	-363.70	-260.45	-20.63	-422.84	-361.04	-325.01	-303.15	-292.20	-1099.15	-161.41
73	-43.64	-367.95	-256.09	-21.44	-424.36	-365.27	-330.61	-309.65	-299.19	-1098.56	-153.47
75	-43.50	-369.71	-254.70	-21.97	-424.45	-366.80	-332.90	-312.53	-302.46	-1099.92	-150.32
80	-43.04	-370.48	-255.50	-23.42	-421.46	-366.67	-334.39	-315.46	-306.57	-1109.53	-147.20
84	-41.70	-366.11	-261.76	-25.16	-414.39	-361.61	-330.61	-312.96	-305.44	-1125.33	-150.25
85	-41.46	-365.35	-262.80	-25.41	-413.35	-360.75	-329.90	-312.41	-305.09	-1127.82	-151.06
90	-39.76	-358.12	-272.61	-28.77	-403.08	-353.57	-324.76	-309.27	-304.90	-1152.08	-163.25
95	-40.66	-346.29	-281.02	-31.02	-390.05	-344.22	-316.04	-302.24	-301.26	-1177.49	-182.13
98	-41.25	-325.63	-296.57	-32.16	-369.87	-322.61	-294.62	-281.75	-282.47	-1212.89	-200.90
100	-41.59	-296.05	-310.30	-32.11	-337.72	-288.45	-264.38	-253.47	-254.70	-1215.95	-220.69

Table S33 Solvation energies $\Delta_{solv}G$ of the second mixture of all species included at infinite dilution relative to the mass percentage of sulfuric acid %. All energies are given in kJ mol^{-1} at 298.15 K and a pressure of 1 bar.

%	$H_4S_2O_8$	$H_5S_2O_8^+$	$H_3S_2O_8^-$	$H_2S_2O_8^{2-}$	$SO_4^{2-} \cdot H_2O$	$SO_4^{2-} \cdot 2\ H_2O$	$HSO_4^- \cdot H_2O$	$H_5SO_5^+$	H_4SO_5	$H_2S_2O_7$
5	-70.37	-308.12	-208.70	-814.94	-998.43	-923.28	-279.29	-322.81	-55.52	-58.74
10	-71.08	-310.23	-209.00	-815.51	-1001.49	-925.75	-279.51	-322.53	-54.97	-59.55
15	-71.64	-311.92	-209.06	-815.69	-1004.11	-927.87	-279.48	-322.26	-54.30	-60.21
20	-72.03	-313.01	-209.24	-816.22	-1007.09	-930.42	-279.58	-321.63	-53.48	-60.70
25	-72.23	-313.64	-209.40	-816.88	-1010.29	-933.23	-279.68	-320.67	-52.48	-60.98
30	-72.04	-313.90	-209.06	-816.99	-1013.35	-935.85	-279.45	-319.64	-51.29	-60.86
35	-71.50	-313.53	-208.66	-817.42	-1016.91	-939.00	-279.32	-318.08	-49.84	-60.38
40	-70.50	-313.08	-207.44	-816.83	-1019.79	-941.45	-278.62	-316.49	-48.07	-59.42
45	-68.54	-312.69	-204.17	-813.36	-1021.18	-942.01	-276.48	-315.68	-46.14	-57.46
50	-65.68	-312.98	-198.49	-806.30	-1020.25	-939.89	-272.60	-316.24	-44.13	-54.55
55	-62.12	-313.88	-191.15	-797.11	-1017.97	-936.29	-267.76	-317.65	-41.94	-50.89
60	-57.38	-317.02	-179.57	-780.95	-1011.61	-927.81	-259.70	-322.48	-39.99	-46.02
64	-52.43	-322.47	-165.95	-761.33	-1004.01	-917.70	-250.32	-330.16	-38.42	-41.04
65	-51.83	-323.35	-164.13	-758.68	-1003.04	-916.40	-249.10	-331.39	-38.29	-40.44
67	-49.63	-326.84	-157.46	-749.23	-999.96	-912.24	-244.77	-335.81	-37.66	-38.32
70	-47.36	-332.46	-149.20	-737.87	-996.82	-908.24	-239.88	-341.79	-36.94	-36.16
73	-46.15	-338.52	-142.41	-728.65	-995.17	-906.05	-236.31	-347.41	-36.53	-34.96
75	-46.31	-341.58	-140.18	-725.66	-996.06	-906.76	-235.56	-349.84	-36.53	-35.04
80	-47.76	-345.77	-139.96	-727.36	-1005.06	-915.84	-238.46	-351.02	-36.11	-36.04
84	-48.51	-344.31	-145.90	-740.48	-1021.01	-932.56	-247.31	-345.53	-35.08	-36.19
85	-48.53	-343.87	-147.06	-742.66	-1023.43	-935.02	-248.73	-344.47	-34.96	-36.11
90	-48.70	-340.21	-162.10	-762.79	-1046.15	-957.62	-263.47	-334.07	-35.95	-35.38
95	-50.74	-331.47	-186.03	-780.49	-1067.57	-977.01	-277.46	-320.43	-40.38	-36.30
98	-52.35	-310.96	-208.93	-812.52	-1100.54	-1008.22	-295.00	-301.01	-42.88	-36.61
100	-54.10	-279.30	-230.22	-846.63	-1108.33	-1022.16	-310.20	-274.93	-44.98	-36.61

3.6.3 Solvation energies obtained from the concentrations of the third mixture

Table S34 Gibbs energies of solvation of the proton $\Delta_{solv}G(H^+)$ at 298.15 K and 1 bar obtained from the third mixture through the respective thermodynamic cycles. Listed are the main thermodynamic cycles with their identified by their numbers and the mass percentage of sulfuric acid %. All energies are given in kJ mol^{-1}.

%	1a	1b	1c	2	3a	3b	3c	3d
5	-1077.36	-1100.86	-1102.74	-966.49	-941.58	-970.47	-1015.74	-1016.39
10	-1073.74	-1097.83	-1100.30	-968.46	-943.26	-971.86	-1015.62	-1016.11
15	-1070.47	-1094.96	-1097.93	-970.00	-944.68	-973.00	-1015.74	-1016.00
20	-1067.02	-1091.80	-1095.21	-970.97	-945.68	-973.69	-1015.72	-1015.65
25	-1063.42	-1088.41	-1092.20	-971.60	-946.49	-974.12	-1015.66	-1015.15
30	-1059.53	-1084.82	-1089.03	-972.16	-947.41	-974.58	-1015.76	-1014.70
35	-1055.21	-1080.84	-1085.50	-972.40	-948.22	-974.80	-1015.66	-1013.93
40	-1050.80	-1077.00	-1082.27	-973.16	-949.90	-975.66	-1016.19	-1013.70
45	-1046.32	-1073.33	-1079.36	-974.43	-952.49	-977.18	-1017.13	-1013.85
50	-1041.94	-1070.19	-1077.22	-976.72	-956.55	-979.97	-1018.73	-1014.79
55	-1037.84	-1067.77	-1076.06	-980.36	-962.51	-984.46	-1021.04	-1016.64
60	-1033.56	-1066.11	-1076.17	-985.72	-970.93	-991.48	-1024.58	-1020.18
64	-1029.02	-1065.20	-1077.66	-993.04	-981.54	-1001.67	-1029.65	-1026.05
65	-1028.30	-1064.98	-1077.78	-993.92	-982.89	-1003.01	-1030.23	-1026.77
67	-1025.63	-1063.95	-1077.91	-997.37	-988.05	-1008.12	-1032.21	-1029.15
70	-1021.74	-1062.10	-1077.48	-1002.07	-995.40	-1015.20	-1034.61	-1032.08
73	-1017.64	-1059.65	-1076.25	-1005.22	-1001.12	-1020.13	-1035.82	-1033.57
75	-1014.69	-1057.54	-1074.84	-1005.87	-1003.25	-1021.48	-1035.63	-1033.32
80	-1005.28	-1050.05	-1069.25	-1004.39	-1005.91	-1021.10	-1031.99	-1028.95
84	-996.02	-1042.81	-1064.12	-1001.17	-1006.98	-1019.07	-1026.45	-1022.72
85	-994.68	-1041.87	-1063.57	-1000.66	-1006.96	-1018.68	-1025.92	-1022.08
90	-981.83	-1033.99	-1060.87	-995.37	-1005.83	-1015.29	-1025.62	-1021.13
95	-964.71	-1022.05	-1055.17	-981.30	-993.73	-1002.74	-1026.28	-1021.31
98	-947.91	-1008.59	-1044.88	-963.22	-975.53	-984.29	-1018.68	-1013.19
100	-954.48	-1012.19	-1045.98	-939.16	-949.90	-957.91	-1017.25	-1006.22

The proton solvation energies $\Delta_{solv}G(H^+)$ calculated through the thermodynamic cycles that were not considered as potential determining species in the aqueous solutions of sulfuric acid are presented in Table S35.

Table S35 Gibbs energies of solvation of the proton $\Delta_{solv}G(H^+)$ at 298.15 K and 1 bar obtained from the third mixture through the thermodynamic cycles of different sulfuric acid derivatives $EH^+ \rightarrow E + H^+$, which are listed here as EH^+/E. All energies are given in kJ mol^{-1} in relation to the mass percentage of sulfuric acid %.

%	H_2SO_4 / HSO_4^-	$H_4S_2O_8$ / $H_3S_2O_8^-$	$H_2S_2O_7$ / $HS_2O_7^-$	$H_5SO_5^+$ / H_4SO_5
5	-1030.39	-1016.14	-1007.82	-1045.06
10	-1030.39	-1016.55	-1008.30	-1045.32
15	-1030.55	-1017.05	-1008.89	-1045.72
20	-1030.50	-1017.26	-1009.21	-1045.90
25	-1030.30	-1017.31	-1009.39	-1045.95
30	-1030.19	-1017.46	-1009.66	-1046.11
35	-1029.80	-1017.37	-1009.69	-1046.06
40	-1029.97	-1017.98	-1010.42	-1046.60
45	-1030.52	-1019.23	-1011.79	-1047.68
50	-1031.88	-1021.61	-1014.29	-1049.84
55	-1034.20	-1025.41	-1018.23	-1053.43
60	-1038.25	-1031.37	-1024.39	-1059.27
64	-1044.82	-1039.87	-1033.06	-1068.33
65	-1045.67	-1040.98	-1034.22	-1069.55
67	-1048.65	-1045.03	-1038.52	-1074.06
70	-1052.52	-1050.63	-1044.91	-1080.20
73	-1054.73	-1054.61	-1050.09	-1084.33
75	-1054.76	-1055.66	-1051.93	-1085.31
80	-1050.46	-1053.72	-1052.20	-1083.44
84	-1042.91	-1047.28	-1047.71	-1078.17
85	-1041.85	-1046.06	-1046.69	-1077.17
90	-1033.79	-1032.49	-1035.37	-1066.35
95	-1023.35	-1011.40	-1018.81	-1048.16
98	-1010.59	-993.73	-1003.37	-1029.92
100	-1001.81	-978.36	-988.08	-1007.71

Table S36 Gibbs energies of solvation of the proton $\Delta_{solv}G(H^+)$ at 298.15 K and 1 bar obtained from the third mixture through the thermodynamic cycles of the protonated water clusters $EH^+ \rightarrow E + H^+$, which are listed here as EH^+/E. All energies are given in kJ mol^{-1} in relation to the mass percentage of sulfuric acid %.

%	H_3O^+ / H_2O	$H_5O_2^+$ / 2 H_2O	$H_7O_3^+$ / 3 H_2O	$H_9O_4^+$ / 4 H_2O	$H_{11}O_5^+$ / 5 H_2O
5	-1043.58	-1053.64	-1049.45	-1041.47	-1027.49
10	-1046.07	-1055.00	-1050.32	-1042.18	-1028.19
15	-1048.21	-1056.40	-1051.30	-1043.07	-1029.10
20	-1049.81	-1057.51	-1052.04	-1043.77	-1029.84
25	-1051.01	-1058.42	-1052.61	-1044.35	-1030.47
30	-1051.91	-1059.29	-1053.24	-1045.02	-1031.15
35	-1052.25	-1059.77	-1053.52	-1045.36	-1031.47
40	-1052.57	-1060.54	-1054.24	-1046.18	-1032.24
45	-1052.77	-1061.47	-1055.25	-1047.33	-1033.26
50	-1053.11	-1062.90	-1056.99	-1049.26	-1034.99
55	-1053.71	-1065.05	-1059.71	-1052.24	-1037.70
60	-1054.59	-1068.52	-1064.22	-1057.14	-1042.26
64	-1056.44	-1074.23	-1071.38	-1064.71	-1049.46
65	-1056.55	-1074.93	-1072.27	-1065.64	-1050.34
67	-1057.23	-1077.55	-1075.46	-1068.94	-1053.40
70	-1057.71	-1080.51	-1079.12	-1072.75	-1056.91
73	-1056.82	-1081.36	-1080.50	-1074.27	-1058.23
75	-1055.28	-1080.59	-1079.89	-1073.70	-1057.56
80	-1048.06	-1074.46	-1073.69	-1067.31	-1050.87
84	-1038.62	-1065.45	-1064.10	-1057.03	-1040.13
85	-1037.36	-1064.15	-1062.68	-1055.49	-1038.54
90	-1025.46	-1052.59	-1049.89	-1041.66	-1024.67
95	-1008.86	-1036.44	-1032.36	-1023.70	-1007.75
98	-989.78	-1015.02	-1010.70	-1002.07	-986.52
100	-962.51	-986.83	-986.15	-979.31	-964.15

Table S37 Solvation energies $\Delta_{solv}G$ of all species included in the third mixture with the concentrations given in Section 3.2 for each concentration of sulfuric acid in mass percent %. All energies are given in kJ mol^{-1} at 298.15 K and a pressure of 1 bar.

%	H_2SO_4	$H_3SO_4^+$	HSO_4^-	H_2O	H_3O^+	$H_5O_2^+$	$H_7O_3^+$	$H_9O_4^+$	$H_{11}O_5^+$	SO_4^{2-}	$HS_2O_7^-$
5	-60.16	-341.94	-300.29	-20.89	-407.57	-332.82	-294.02	-270.76	-261.94	-1083.06	-223.08
10	-60.38	-344.13	-300.51	-20.71	-409.87	-333.81	-294.33	-270.72	-261.71	-1086.90	-223.41
15	-60.51	-345.80	-300.48	-20.48	-411.79	-334.75	-294.62	-270.70	-261.48	-1090.14	-223.48
20	-60.56	-346.82	-300.58	-20.21	-413.13	-335.33	-294.56	-270.34	-260.89	-1093.68	-223.65
25	-60.47	-347.36	-300.68	-19.91	-414.01	-335.63	-294.23	-269.70	-260.00	-1097.40	-223.75
30	-60.14	-347.58	-300.47	-19.60	-414.60	-335.88	-293.92	-269.12	-259.12	-1101.07	-223.37
35	-59.55	-347.24	-300.26	-19.29	-414.64	-335.74	-293.29	-268.24	-257.92	-1105.18	-222.81
40	-58.54	-346.98	-299.08	-19.02	-414.69	-335.97	-293.18	-267.96	-257.29	-1108.41	-220.90
45	-57.07	-346.79	-297.06	-18.81	-414.68	-336.47	-293.55	-268.27	-257.27	-1110.87	-217.65
50	-55.08	-347.09	-293.71	-18.72	-414.92	-337.72	-295.02	-269.84	-258.55	-1111.91	-212.41
55	-52.58	-348.23	-288.90	-18.78	-415.59	-340.01	-297.95	-273.09	-261.60	-1111.19	-204.84
60	-49.63	-350.65	-281.90	-19.06	-416.75	-344.03	-303.28	-279.08	-267.53	-1108.47	-194.04
64	-46.93	-355.26	-272.63	-19.53	-419.07	-350.69	-311.86	-288.55	-277.10	-1103.74	-180.35
65	-46.63	-355.84	-271.48	-19.62	-419.27	-351.56	-313.02	-289.84	-278.42	-1103.31	-178.59
67	-45.53	-358.19	-267.40	-19.94	-420.27	-354.83	-317.17	-294.42	-283.10	-1101.90	-172.16
70	-44.43	-361.79	-262.42	-20.60	-421.40	-359.09	-322.80	-300.85	-289.87	-1100.81	-163.83
73	-43.93	-364.44	-259.72	-21.42	-421.34	-361.59	-326.64	-305.66	-295.30	-1102.21	-158.28
75	-43.73	-364.89	-259.48	-21.94	-420.32	-361.86	-327.59	-307.16	-297.23	-1104.92	-156.63
80	-42.83	-362.51	-262.89	-23.51	-414.66	-358.87	-326.10	-307.04	-298.38	-1117.74	-156.92
84	-41.41	-357.87	-269.02	-25.55	-407.26	-353.94	-322.63	-304.93	-297.84	-1133.14	-161.02
85	-41.24	-357.19	-269.91	-25.84	-406.30	-353.22	-322.08	-304.55	-297.70	-1135.36	-161.97
90	-40.33	-351.00	-277.06	-28.84	-397.40	-347.66	-318.30	-302.74	-298.86	-1155.37	-172.92
95	-40.77	-337.36	-287.94	-30.72	-382.68	-335.26	-306.40	-292.28	-291.32	-1183.36	-189.77
98	-41.26	-319.78	-301.19	-31.94	-364.82	-316.30	-288.42	-275.56	-276.22	-1213.41	-205.34
100	-41.59	-296.05	-310.30	-32.11	-337.72	-288.45	-264.38	-253.47	-254.70	-1215.95	-220.69

Table S38 Solvation energies $\Delta_{solv}G$ of the third mixture of all species included at infinite dilution relative to the mass percentage of sulfuric acid %. All energies are given in kJ mol^{-1} at 298.15 K and a pressure of 1 bar.

%	$H_4S_2O_8$	$H_5S_2O_8^+$	$H_3S_2O_8^-$	$H_2S_2O_8^{2-}$	$SO_4^{2-} \cdot H_2O$	$SO_4^{2-} \cdot 2\ H_2O$	$HSO_4^- \cdot H_2O$	$H_5SO_5^+$	H_4SO_5	$H_2S_2O_7$
5	-70.37	-308.12	-208.70	-814.94	-998.43	-923.28	-279.29	-322.81	-55.52	-58.74
10	-71.08	-310.23	-209.00	-815.51	-1001.49	-925.75	-279.51	-322.53	-54.97	-59.55
15	-71.64	-311.92	-209.06	-815.69	-1004.11	-927.87	-279.48	-322.26	-54.30	-60.21
20	-72.03	-313.01	-209.24	-816.22	-1007.09	-930.42	-279.58	-321.63	-53.48	-60.70
25	-72.23	-313.64	-209.40	-816.88	-1010.29	-933.23	-279.68	-320.67	-52.48	-60.98
30	-72.04	-313.90	-209.06	-816.99	-1013.35	-935.85	-279.45	-319.64	-51.29	-60.86
35	-71.45	-313.53	-208.55	-817.25	-1016.82	-938.88	-279.24	-318.17	-49.88	-60.33
40	-70.24	-313.18	-206.73	-815.66	-1019.21	-940.65	-278.06	-317.03	-48.20	-59.15
45	-68.36	-312.83	-203.61	-812.39	-1020.64	-941.30	-276.02	-316.18	-46.26	-57.28
50	-65.67	-312.92	-198.53	-806.37	-1020.35	-939.98	-272.65	-316.23	-44.16	-54.53
55	-62.14	-313.88	-191.20	-797.19	-1018.01	-936.35	-267.79	-317.56	-41.89	-50.91
60	-57.64	-316.40	-180.74	-783.19	-1012.95	-929.48	-260.75	-321.19	-39.69	-46.27
64	-52.67	-321.63	-167.27	-763.85	-1005.07	-919.18	-251.46	-328.74	-38.17	-41.25
65	-52.07	-322.37	-165.56	-761.42	-1004.24	-918.02	-250.34	-329.82	-38.03	-40.64
67	-49.92	-325.32	-159.37	-752.85	-1001.50	-914.25	-246.38	-333.58	-37.28	-38.52
70	-47.98	-330.47	-151.82	-742.37	-999.03	-910.86	-241.91	-339.09	-36.66	-36.58
73	-47.77	-335.19	-147.63	-736.70	-999.60	-911.14	-240.13	-343.15	-36.59	-36.21
75	-48.15	-336.92	-146.97	-736.28	-1001.99	-913.51	-240.58	-344.06	-36.51	-36.40
80	-49.40	-337.79	-150.14	-743.82	-1014.46	-926.36	-246.27	-341.57	-35.88	-36.95
84	-49.67	-336.02	-156.86	-756.77	-1029.86	-942.50	-255.24	-335.79	-35.38	-36.56
85	-49.69	-335.65	-158.10	-758.65	-1031.99	-944.64	-256.54	-334.80	-35.39	-36.49
90	-50.14	-332.72	-172.12	-773.62	-1050.03	-962.19	-268.38	-325.94	-37.35	-36.13
95	-51.45	-321.47	-194.52	-795.84	-1074.71	-985.12	-283.74	-311.37	-40.97	-36.42
98	-52.69	-304.27	-213.43	-822.87	-1102.65	-1011.63	-298.72	-295.41	-43.25	-36.54
100	-54.10	-279.30	-230.22	-846.63	-1108.33	-1022.16	-310.20	-274.93	-44.98	-36.61

4 Literature

[1] W. M. Haynes, D. R. Lide, T. J. Bruno, *CRC Handbook of Chemistry and Physics*, CRC Press, 2016.

[2] R. J. Gillespie, J. V. Oubridge, C. Solomons, *J. Chem. Soc.* 1957, *0*, 1804.

[3] G. R. Fulmer, A. J. M. Miller, N. H. Sherden, H. E. Gottlieb, A. Nudelman, B. M. Stoltz, J. E. Bercaw, K. I. Goldberg, *Organometallics* 2010, *29*, 2176.

[4] N. V. Ignat'ev, U. Welz-Biermann, A. Kucheryna, G. Bissky, H. Willner, *J. Fluorine Chem.* 2005, *126*, 1150.

[5] M. Mezger, B. M. Ocko, H. Reichert, M. Deutsch, *Proc. Natl. Acad. Sci. U.S.A.* 2013, *110*, 3733.

[6] I. V. Voroshylova, F. Teixeira, R. Costa, C. M. Pereira, M. N. D. S. Cordeiro, *Phys. Chem. Chem. Phys.* 2016, *18*, 2617.

[7] V. Radtke, N. Gebel, D. Priester, A. Ermantraut, M. Bäuerle, D. Himmel, R. Stroh, T. Koslowski, I. Leito, I. Krossing, *Chem. Eur. J.* 2022, *28*, e202200509.

[8] V. Radtke, D. Priester, A. Heering, C. Müller, T. Koslowski, I. Leito, I. Krossing, *Chem. Eur. J.* 2023, *29*, e202300609.

[9] a) Frank Neese, *Wiley Interdiscip. Rev.: Comput. Mol. Sci.* 2012, *2*, 73; b) Frank Neese, *Wiley Interdiscip. Rev.: Comput. Mol. Sci.* 2018, *8*, e1327; c) F. Neese, F. Wennmohs, U. Becker, C. Riplinger, *J. Chem. Phys.* 2020, *152*, 224108; d) Frank Neese, *Wiley Interdiscip. Rev.: Comput. Mol. Sci.* 2022, e1606.

[10] S. Kozuch, J. M. L. Martin, *Phys. Chem. Chem. Phys.* 2011, *13*, 20104.

[11] F. Weigend, R. Ahlrichs, *Phys. Chem. Chem. Phys.* 2005, *7*, 3297.

[12] F. Weigend, *J. Comput. Chem.* 2008, *29*, 167.

[13] A. Hellweg, C. Hättig, S. Höfener, W. Klopper, *Theor. Chem. Acc.* 2007, *117*, 587.

[14] a) S. Grimme, J. Antony, S. Ehrlich, H. Krieg, *J. Chem. Phys.* 2010, *132*, 154104; b) S. Grimme, S. Ehrlich, L. Goerigk, *J. Comput. Chem.* 2011, *32*, 1456.

[15] S. Kozuch, D. Gruzman, J. M. L. Martin, *J. Phys. Chem. C* 2010, *114*, 20801.

[16] L. Goerigk, A. Hansen, C. Bauer, S. Ehrlich, A. Najibi, S. Grimme, *Phys. Chem. Chem. Phys.* 2017, *19*, 32184.

[17] a) C. Riplinger, F. Neese, *J. Chem. Phys.* 2013, *138*, 34106; b) C. Riplinger, B. Sandhoefer, A. Hansen, F. Neese, *J. Chem. Phys.* 2013, *139*, 134101.

[18] T. H. Dunning, *J. Chem. Phys.* 1989, *90*, 1007.

[19] D. E. Woon, T. H. Dunning, *J. Chem. Phys.* 1993, *98*, 1358.

[20] C. Hättig, *Phys. Chem. Chem. Phys.* 2005, *7*, 59.

[21] a) A. Klamt, *J. Phys. Chem.* 1995, *99*, 2224; b) A. Klamt, V. Jonas, T. Bürger, J. C. W. Lohrenz, *J. Phys. Chem. A* 1998, *102*, 5074; c) F. Eckert and A. Klamt, *AIChE J.* 2002, *48*, 369.

[22] BIOVIA COSMOtherm, *Release 2023*, Dassault Systèmes, http://www.3ds.com.

[23] TURBOMOLE V7.5.1 2021, a development of University of Karlsruhe and Forschungszentrum Karlsruhe GmbH, 1989-2007, TURBOMOLE GmbH, since 2007, available from https://www.turbomole.org.

[24] a) A. D. Becke, *Phys. Rev. A* 1988, *38*, 3098; b) J. P. Perdew, *Phys. Rev. B* 1986, *33*, 8822; c) J. P. Perdew, *Phys. Rev. B* 1986, *34*, 7406.

[25] A. Klamt, M. Diedenhofen, *J. Comput. Chem.* 2018, *39*, 1648.

[26] V. Barone, M. Cossi, *J. Phys. Chem. A* 1998, *102*, 1995.

[27] V. Kazansky, V. Solkan, *Phys. Chem. Chem. Phys.* 2003, *5*, 31.

[28] R. J. Gillespie, E. A. Robinson, C. Solomons, *J. Chem. Soc.* 1960, 4320.

[29] R. J. Gillespie, S. Wasif, *J. Chem. Soc.* 1953, 964.

[30] a) S. Rondinini, P. Longhi, P. R. Mussini, T. Mussini, *Pure Appl. Chem.* 1987, *59*, 1693; b) M. I. Vinnik, R. S. Ryabova, *Zh. Fiz. Khim.* 1964, *38*, 606; c) B. J. Kirkbride, P. A. H. Wyatt, *Trans. Faraday Soc.* 1958, *54*, 483; d) R. H. Flowers, R. J. Gillespie, E. A. Robinson, *Can. J. Chem.* 1960, *38*, 1363.

[31] K. Do, T. P. Klein, C. A. Pommerening, S. M. Bachrach, L. S. Sunderlin, *J. Am. Chem. Soc.* 1998, *120*, 6093.

[32] A. A. Viggiano, M. J. Henchman, F. Dale, C. A. Deakyne, J. F. Paulson, *J. Am. Chem. Soc.* 1992, *114*, 4299.

[33] F. H. Rhodes, C. B. Barbour, *Ind. Eng. Chem.* 1923, *15*, 850.
[34] M. Bäuerle, Investigations into Direct Potential Measurements in Pure Sulfuric Acid Using an Ideal Ionic Liquid Salt Bridge, *Doctoral dissertation*, University of Freiburg, Germany, 2024.
[35] R. J. Gillespie, T. E. Peel, E. A. Robinson, *J. Am. Chem. Soc.* 1971, *93*, 5083.
[36] L. P. Hammett, A. J. Deyrup, *J. Am. Chem. Soc.* 1932, *54*, 2721.
[37] A. Ahmed, S. I. Sandler, *J. Chem. Theory Comput.* 2013, *9*, 2774.
[38] T. F. Young, G. E. Walrafen, *Trans. Faraday Soc.* 1961, *57*, 34.
[39] T. F. Young, L. F. Maranville, and H. M. Smith, *The Structure of Electrolytic Solutions*. W. J. Hamer, ed., Wiley, New York, 1959.
[40] E. B. Robertson, H. B. Dunford, *J. Am. Chem. Soc.* 1964, *86*, 5080.
[41] D. Fraenkel, *New J. Chem.* 2015, *39*, 5124.
[42] F. Murillo, A. Vargas-Caamal, S. Pan, J. L. Cabellos, M. J. Mora-Fonz, A. Muñoz-Castro, A. Restrepo, G. Merino, *Phys. Chem. Chem. Phys.* 2017, *19*, 17088.
[43] V. Radtke, M. Bäuerle, R. Stroh, T. Kienzle, D. Himmel, A. Heering, J. Nerut, E. Lust, I. Leito, and I. Krossing 2024, *About a Trillion Times more Acidic than Expected? On the Determination of the Unified Acidity of Sulfuric Acid*, Manuscript in preparation.
[44] A. Das, S. Dev, H. Shangpliang, K. L. Nonglait, K. Ismail, *J. Phys. Chem. B* 1997, *101*, 4166.
[45] H. E. Darling, *J. Chem. Eng. Data* 1964, *9*, 421.
[46] G. E. Walrafen, W.-H. Yang, Y. C. Chu, M. S. Hokmabadi, *J. Solution Chem.* 2000, *29*, 905.
[47] G. E. Walrafen, W.-H. Yang, Y. C. Chu, *J. Phys. Chem. A* 2002, *106*, 10162.
[48] G. Herzberg, *Molecular Spectra and Molecular Structure, Vol. 2, Infrared and Raman Spectra of Polyatomic Molecules*, Van Nostrand Reinhold, New York, 1945.
[49] Frank Neese, Frank Wennmohs, *ORCA - An ab initio, DFT and semiempirical SCF-MO package*. *Version 5.0.3*, Mulheim a. d. Ruhr, Germany.
[50] E. P. L. Hunter, S. G. Lias, *J. Phys. Chem. Ref. Data* 1998, *27*, 413.
